# DUSP5 expression in left ventricular cardiomyocytes of young hearts regulates thyroid hormone (T3)-induced proliferative ERK1/2 signaling

**DOI:** 10.1038/s41598-020-78825-x

**Published:** 2020-12-14

**Authors:** Nikolay Bogush, Lin Tan, Hussain Naib, Ebrahim Faizullabhoy, John W. Calvert, Siiri E. Iismaa, Ankan Gupta, Ramani Ramchandran, David I. K. Martin, Robert M. Graham, Ahsan Husain, Nawazish Naqvi

**Affiliations:** 1 Department of Medicine (Cardiology), Emory University School of Medicine, 323 WMRB, 101 Woodruff Circle, Atlanta, GA 30322 USA; 2 Department of Surgery, Carlyle Fraser Heart Center, Emory University School of Medicine, Atlanta, GA USA; 3 Victor Chang Cardiac Research Institute, Darlinghurst, NSW Australia; 4 Developmental Vascular Biology Program, Division of Neonatology, Department of Pediatrics, Department of Obstetrics and Gynecology, Children’s Research Institute, Medical College of Wisconsin, Milwaukee, WI USA; 5 Children’s Hospital Oakland Research Institute, Oakland, CA USA

**Keywords:** Cell signalling, Cardiovascular biology, Cell biology

## Abstract

Cardiomyocytes of newborn mice proliferate after injury or exposure to growth factors. However, these responses are diminished after postnatal day-6 (P6), representing a barrier to building new cardiac muscle in adults. We have previously shown that exogenous thyroid hormone (T3) stimulates cardiomyocyte proliferation in P2 cardiomyocytes, by activating insulin-like growth factor-1 receptor (IGF-1R)-mediated ERK1/2 signaling. But whether exogenous T3 functions as a mitogen in post-P6 murine hearts is not known. Here, we show that exogenous T3 increases the cardiomyocyte endowment of P8 hearts, but the proliferative response is confined to cardiomyocytes of the left ventricular (LV) apex. Exogenous T3 stimulates proliferative ERK1/2 signaling in apical cardiomyocytes, but not in those of the LV base, which is inhibited by expression of the nuclear phospho-ERK1/2-specific dual-specificity phosphatase, DUSP5. Developmentally, between P7 and P14, DUSP5 expression increases in the myocardium from the LV base to its apex; after this period, it is uniformly expressed throughout the LV. In young adult hearts, exogenous T3 increases cardiomyocyte numbers after DUSP5 depletion, which might be useful for eliciting cardiac regeneration.

## Introduction

The rodent heart grows rapidly after birth; quadrupling in size over a period of just a few weeks^1^. A key feature of this growth is an increase in stroke volume^1^, commensurate with the rapid expansion of the circulatory system during postnatal development. As the newborn heart grows, its constituent cardiomyocytes mature. The increase in the mass of the left ventricle (LV) together with the fetal-to-adult shift in gene expression patterns (e.g., increased postnatal expression of *Mhy6*, the gene encoding α-myosin heavy chain^2^) increase LV contractile performance^1^. Recent studies suggest that the young rodent heart grows through cardiomyocyte hypertrophy^1^ and proliferation^1,3–5^; estimates suggest that cardiomyocyte proliferation could be as high as 30–60% during the first week of life^1,4,5^ and about 30–40% in the second week of life^1^. The signals that stimulate cardiomyocyte proliferation in the former period are unknown, but it has been proposed that in the second week of life cardiomyocyte proliferation is triggered by a developmental surge in thyroid hormone (T3)^1^. The use of differing methodologies to block the endogenous T3 effect^1,6^, however, has resulted in conflicting views about the utility of T3 in driving in vivo cardiomyocyte proliferation and hence building heart muscle.

Here, we evaluated if exogenous administration of T3 activates cardiomyocyte proliferation in post-postnatal day-6 (P6) hearts. In these studies, we controlled for litter size to minimize any natural variability in body growth, which positively regulates developmental cardiomyocyte proliferation^3^. Additionally, we focused on the developmental period just before endogenous circulating T3 levels increase, which starts at ~ P10–P11^1,7^, so as not to confuse the actions of endogenous and exogenous T3. We show that T3 stimulates P8 cardiomyocytes to proliferate in vivo, albeit in a spatially-restricted manner, with only cells of the LV apex responding, and provide evidence for a mechanism that regulates this heterogeneity.

## Results

### LV apical cardiomyocytes of post-P6 hearts proliferate in response to exogenous T3

**Figure 1:**
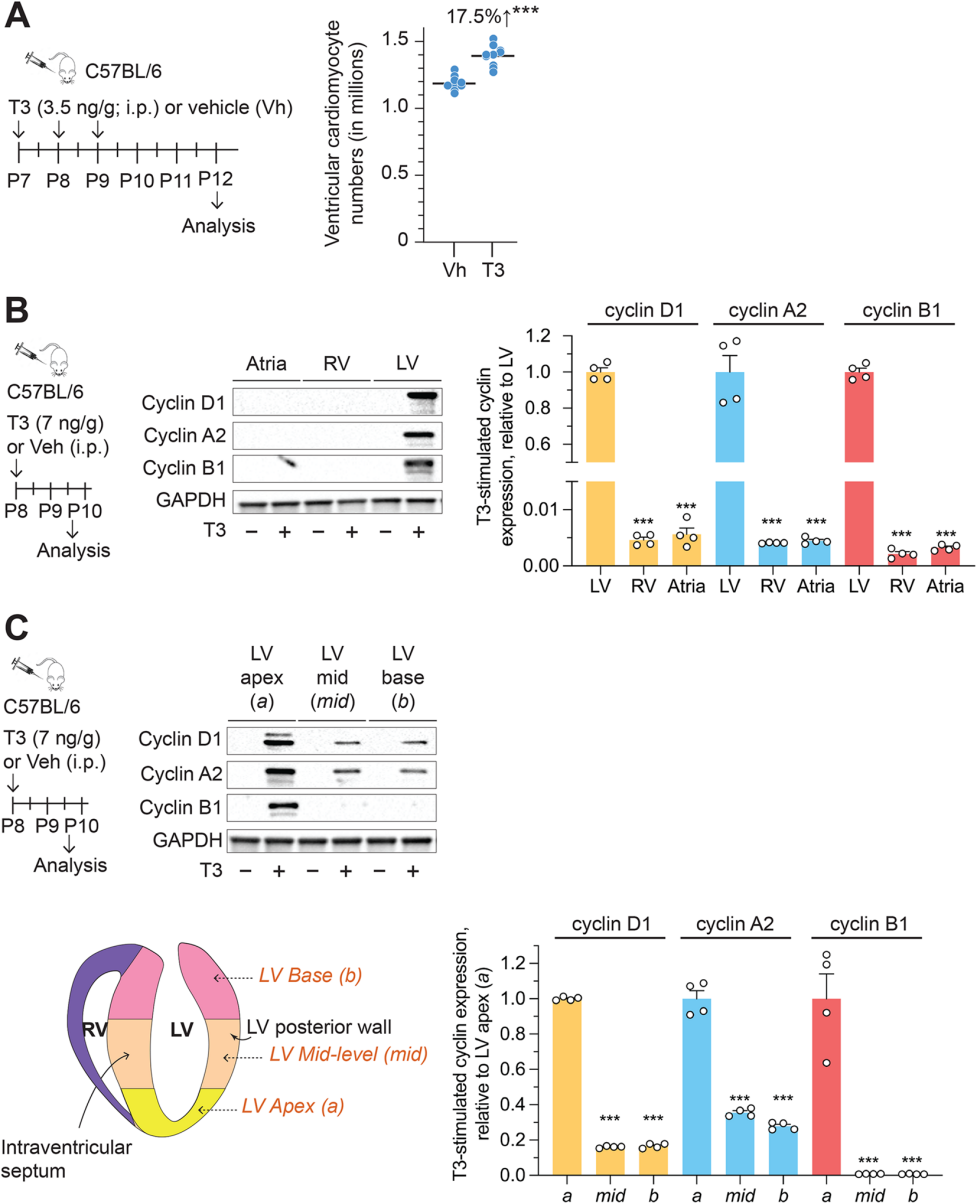
T3 increases ventricular cardiomyocyte proliferation and cyclin expression in the LV myocardium of post-P6 hearts. (**A**) Exogenous T3 administration increases ventricular cardiomyocyte numbers in P7 mice. ***P < 0.001. Schematics illustrate experimental protocol. (**B**,**C**) In vivo T3 administration increases cyclin D1, A2, and B1 expression in the LV, but not atria or right ventricle (RV) (**B**) and, within the LV, preferentially in the apex compared to the mid LV or base (**C**). Histograms show the quantitation of the data in (**B**,**C**). Immunoblots in (**B**,**C**) are representative of 4 biologically independent replicates. Schematics illustrate experimental protocol. Error bars indicate SEM. ***P < 0.001.

To determine if post-P6 ventricular cardiomyocytes retain proliferative capacity, we administered T3 daily (3.5 ng/g, daily, i. p.), from P7 to P9. At P12, hearts were enzymatically disaggregated and cell suspensions created from the cardiac ventricles. Digestion efficiencies were ~ 98% (97.9 ± 1.1% and 98.6 ± 0.21% in vehicle- and T3-treated hearts, respectively; *P* = 0.53; *n* = 4). Cardiomyocytes in these cell suspensions, identified by their size and by rod shape, were counted using a hemocytometer. We found that, in P7 mice, exogenous T3 administration increased ventricular cardiomyocyte numbers by ~ 18% (*P* < 0.001) (Fig. 1A).

We then sought to determine if the proliferative response to T3 occurs uniformly in all LV cardiomyocytes. Initially, we evaluated cyclins D1, A2 and B1 responses to T3; these cyclins promote G1/S phase transition, S phase, and G2/M phase transition, respectively. Forty hours after an in vivo T3 challenge in P8 mice, expression of cyclins D1, A2 and B1 was increased only in the LV (Fig. 1B) and, within the LV, these cyclins were primarily expressed in myocardial tissue of the apex relative to that of the base (*P* < 0.001) (Fig. 1C).

**Figure 2:**
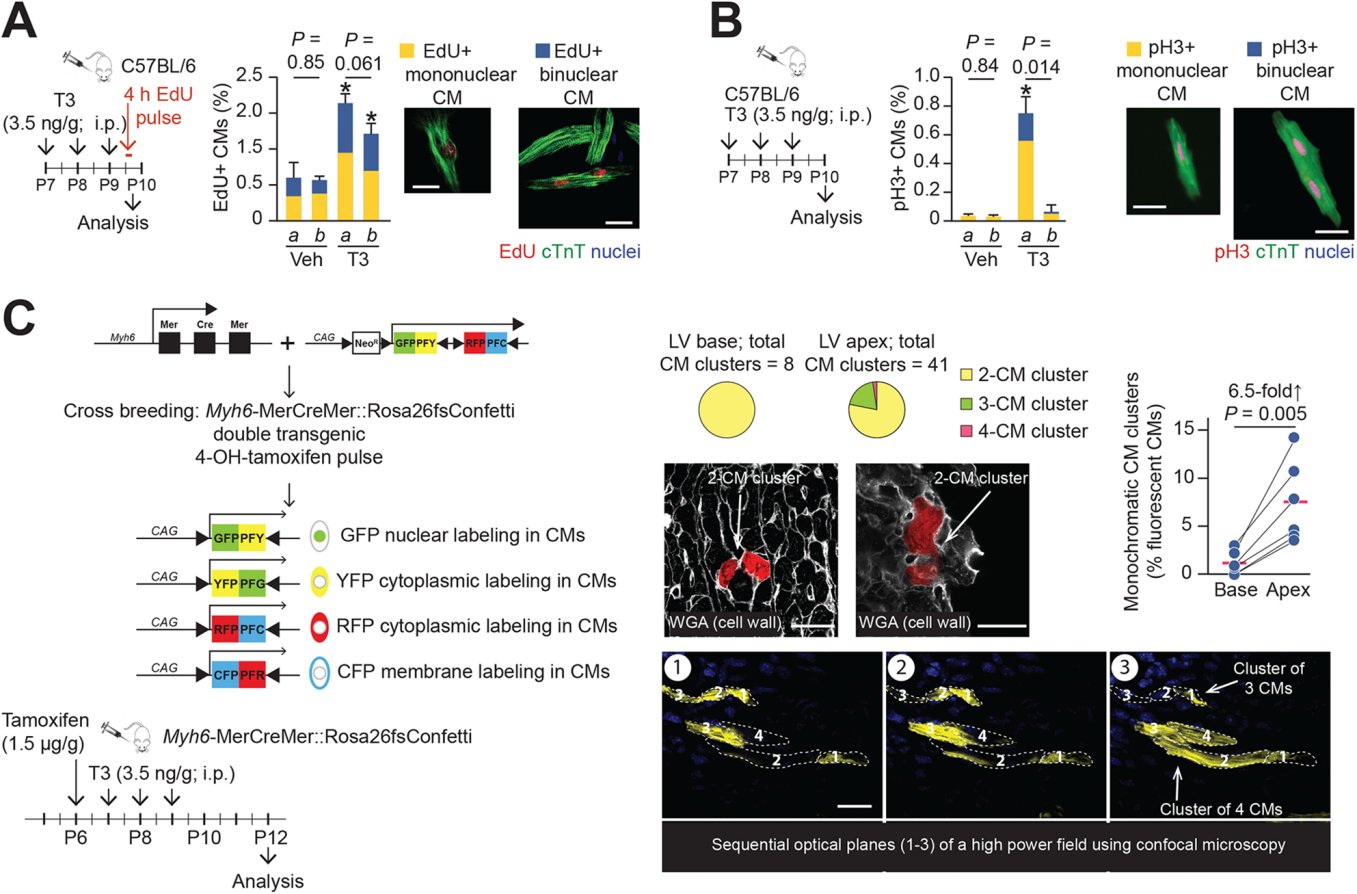
LV apical cardiomyocytes (CM) of post-P6 hearts proliferate in response to exogenous T3 versus vehicle. (**A**,**B**) Effects of T3 on DNA synthesis (EdU labeling) (**A**) and mitosis (pH3 labeling) (**B**) in cardiomyocytes from LV apex (a) or base (b). Cardiomyocytes were identified by cTnT labeling. *P < 0.05 for vehicle (Veh) versus T3 comparisons within each region using an unpaired Student’s *t*-test; *n* = 4. Intra-LV comparisons were made using a paired Student’s *t*-test; *P*-values for these comparisons are indicated. Between 500 and 2000 cardiomyocytes were analyzed for each assessment. (**C**) Multicolor labeling of post-neonatal mouse LV cardiomyocytes. Limited 4-hydroxytamoxifen-induced recombination of paired *loxP* sites (black triangles) leads to expression of green fluorescent protein (GFP), yellow fluorescent protein (YFP), red fluorescent protein (RFP) or cyan fluorescent protein (CFP) in a few cardiomyocytes, randomly positioned within the entire LV. Insets show examples of monochromatic clusters. Intra-LV comparisons were made using a paired Students *t*-test. Schematics illustrate experimental protocol. Error bars indicate SEM. White bars in photomicrographs represent 20 µm.

Next, we studied whether cardiomyocytes of the P8 LV apex and the LV base respond differentially to exogenous T3, as implied by our tissue cyclin expression studies. We examined cell cycle reentry by 5-ethynyl-2′-deoxyuridine (EdU) incorporation. We also assayed mitosis by the presence of phosphorylated histone-3 (pH3). Administration of T3 increased EdU and pH3 labeling in cardiomyocytes of the LV apex (Fig. 2A,B), with cell cycle entry and mitosis being observed in both mono- and binucleated LV apical cardiomyocytes (Fig. 2B). While cell cycle entry was seen in cardiomyocytes derived from both the LV apex and base, T3-stimulated mitosis was evident only in apical cardiomyocytes (Fig. 2A,B).

To confirm that T3-stimulated cardiomyocyte replication is indeed limited to cells in the LV apex, we used a genetic lineage tracing approach. Specifically, we evaluated the proliferative strategies of individual cardiomyocytes of the LV apex relative to those of the base using multicolor clonal analysis of cardiomyocytes in double transgenic *Myh6*-MerCreMer::Rosa26fs-Confetti mice^8^. In this double transgenic model, limited Cre activation (by administration of 10 mg/kg 4-hydroxytamoxifen to P6 mice) resulted in cardiomyocytes being labeled with either RFP, YFP, CFP or GFP. The frequency of RFP and YFP labeling events was < 1.5%, each, while cardiomyocytes labeled with membrane-targeted CFP and nuclear-targeted GFP were much more infrequent. By contrast, at higher 4-hydroxytamoxifen doses, a greater percentage of cardiomyocytes were CFP-labeled and bichromatic cardiomyocytes were also observed (data not shown). In subsequent studies with T3 administration to ~ P8 mice, we relied on quantifying monochromatic cluster frequencies of RFP- and YFP-labeled cardiomyocytes after activation of Cre expression using 10 µg/g 4-hydroxytamoxifen.

In 4-hydroxytamoxifen-pretreated *Myh6*-MerCreMer::Rosa26fs-Confetti mice, T3, given daily over 3 days from P7 to P9, increased the relative frequency of monochromatic cardiomyocyte clusters by ~ 6.5-fold in the LV apex relative to its base (*P* = 0.005) (Fig. 2C). The majority of monochromatic cardiomyocyte clusters in the LV apex consisted of 2 cells (Fig. 2C), and were distributed throughout the tissue section, indicating that cardiomyocyte proliferation resulted from multiple individual cell replication events rather than clonal expansion of a few highly proliferative cells. In addition, monochromatic clusters of > 2 cells were observed in the LV apex, but never in the LV base. These studies indicate that T3-stimulated cardiomyocyte proliferation in early post-P6 LVs is spatially restricted to the apex.

### Spatial heterogeneity of proliferative signaling in the developing postnatal heart

Our earlier studies, using pups from litters of ~ 7/dam showed that a substantial surge in circulating T3 levels that occurs developmentally from P12, is associated with a transient induction of cardiomyocyte proliferation during mid-preadolescence that is at P15^1^. We also show that this developmental phase of cardiomyocyte proliferation can be blocked by inhibiting T3 biosynthesis with propylthiouracil (PTU) given from P7^1^. Thus, we next tested whether the response to endogenous T3 is restricted to cardiomyocytes of the LV apex and is associated with maturation of the heart during this critical developmental window. Mitogenic stimuli elicit sustained increases in phosphorylated (p)-ERK1/2 that in turn activate multiple gene programs, resulting in effector proteins synthesis (such as cyclins D1, A2) and cell proliferation^9,10^. Here, we used p-ERK1/2 and cyclin A2 expression as surrogates for proliferative signaling. Also, we examined if this apex-restricted proliferative signaling phenotype is dependent on the developmental surge in circulating T3 during the second week of life.

**Figure 3:**
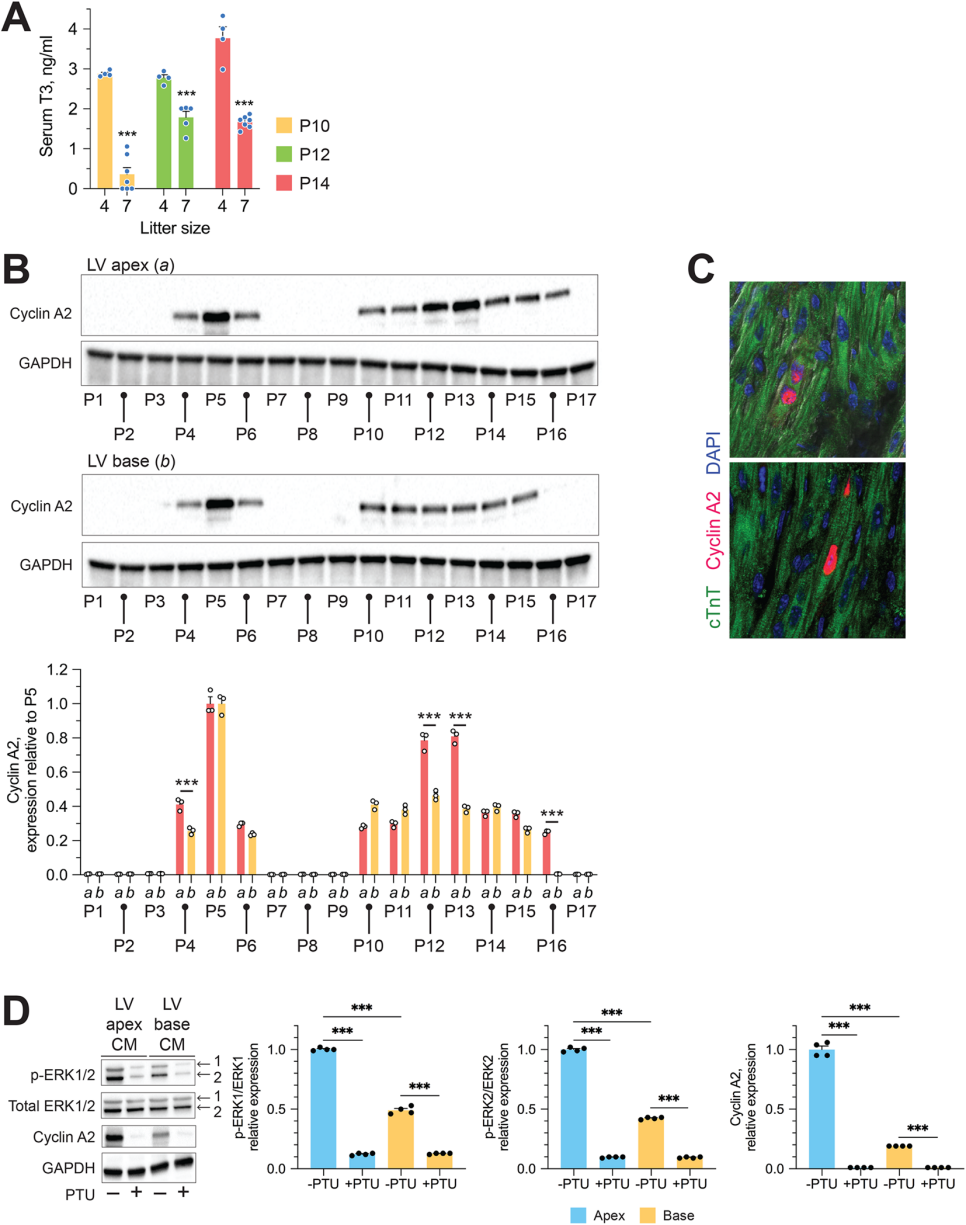
Spatial restriction of cardiomyocytes proliferative signaling to the LV apex during preadolescence. (**A**) Effect of litter size on developmental surge in circulating T3 during early preadolescence. (**B**) Representative immunoblots showing developmental changes in myocardial Cyclin A2 levels in the LV apex (*a*) and base (*b*) of early postnatal hearts. Bar graphs show GAPDH-normalized cyclin A2 levels relative to levels in P5 LV myocardium. (**C**) Two examples of a cyclin A2^+^ cardiomyocyte in a P13 LV apical tissue section. No cyclin A2^+^ cardiomyocytes were seen on sections of P13 LV base. (**D**) Representative immunoblots show expression levels of indicated proteins in whole cardiomyocyte (CM) lysates obtained from CMs of the LV apex and base at P13. Immunoblots show that developmental increases in p-ERK1/2 and Cyclin A2 in LV apical CMs of P13 mice are suppressed by PTU treatment that blocks T3 biosynthesis. Quantitative data is shown next to the immunoblots. ***P < 0.001. Data are mean ± SEM.

Suckling of pups impacts on the timing of the natural surge that occurs in circulating T3 levels during preadolescence^11^. Adequate supply of milk for each mouse and therefore litter size is also known to impact cardiomyocyte number during the first 3-weeks after birth^3^. To minimize natural intra-litter variation in circulating T3 levels and body growth during preadolescence, we adjusted litter sizes to 4 pups/dam at P2 so that every pup could access a teat and have an adequate supply of milk. As shown in Fig. 3A, circulating T3 levels rise from P10 to P14 in pups from litters of 4/dam, as we previously reported in pups from litters of ~ 7/dam^1^, however, in the former, the developmental surge occurred 2-days earlier and the levels of circulating T3 during preadolescence were significantly higher.

Evaluation of cyclin A2 expression in the postnatal period revealed that it is transiently induced during the neonatal period starting at P4, with peak cyclin A2 levels at P5 that then wane by the end of neonatal period (P6) (Fig. 3B). After a brief quiescence, during the early post-P6 period, the expression of cell cycle promoting cyclin A2 increased significantly in the LV apex but not LV base, starting at P12, peaking at P13 and then declining after P15 to undetectable levels (Fig. 3B). While cyclin A2^+^ cardiomyocytes in the P13 LV apex were readily detectible by immunohistochemical staining (Fig. 3C), we could not find any cyclin A2^+^ cardiomyocyte in the P13 LV base (data not shown). To determine if this characteristic LV apex-restricted proliferative signaling of the preadolescent heart is dependent on developmental surge in circulating T3, we inhibited T3 biosynthesis by administrating PTU from P7 to P13, as previously reported^1^. We show that cardiomyocytes from the LV apex but not the LV base of the preadolescent murine heart show increased cardiomyocyte expression of p-ERK1/2 and cyclin A2––effects that are inhibited if we block T3 biosynthesis with PTU (Fig. 3D). Collectively, these studies show that during the second week of life cardiomyocytes in the developing murine LV are inherently heterogeneous in their response to developmental growth cues.

### DUSP5 inhibits T3-stimulated ERK1/2 phosphorylation in postnatal cardiomyocytes

T3-receptor α (TRα) mediates the proliferative action of T3 in murine neonatal (P2) cardiomyocytes by activation of IGF-1/IGF-1R^12^, which involves Ras/Raf/MAPK kinase (MEK)/ERK signaling^9^. MEK1/2 activates ERK1/2 by phosphorylating T202 and Y204^9^, which is required for ERK1/2 translocation to the nucleus^10^. Sustained increases in p-ERK1/2 in the nucleus activate multiple transcription factors, ultimately resulting in effector protein synthesis and cell proliferation^9^. We tested whether the difference in responsiveness between cardiomyocytes of the P8 LV apex and base to exogenous T3 is due to differential TRα expression in these cells or to subsequent T3/IGF-1/IGF-1R/ERK1/2 signaling.

**Figure 4:**
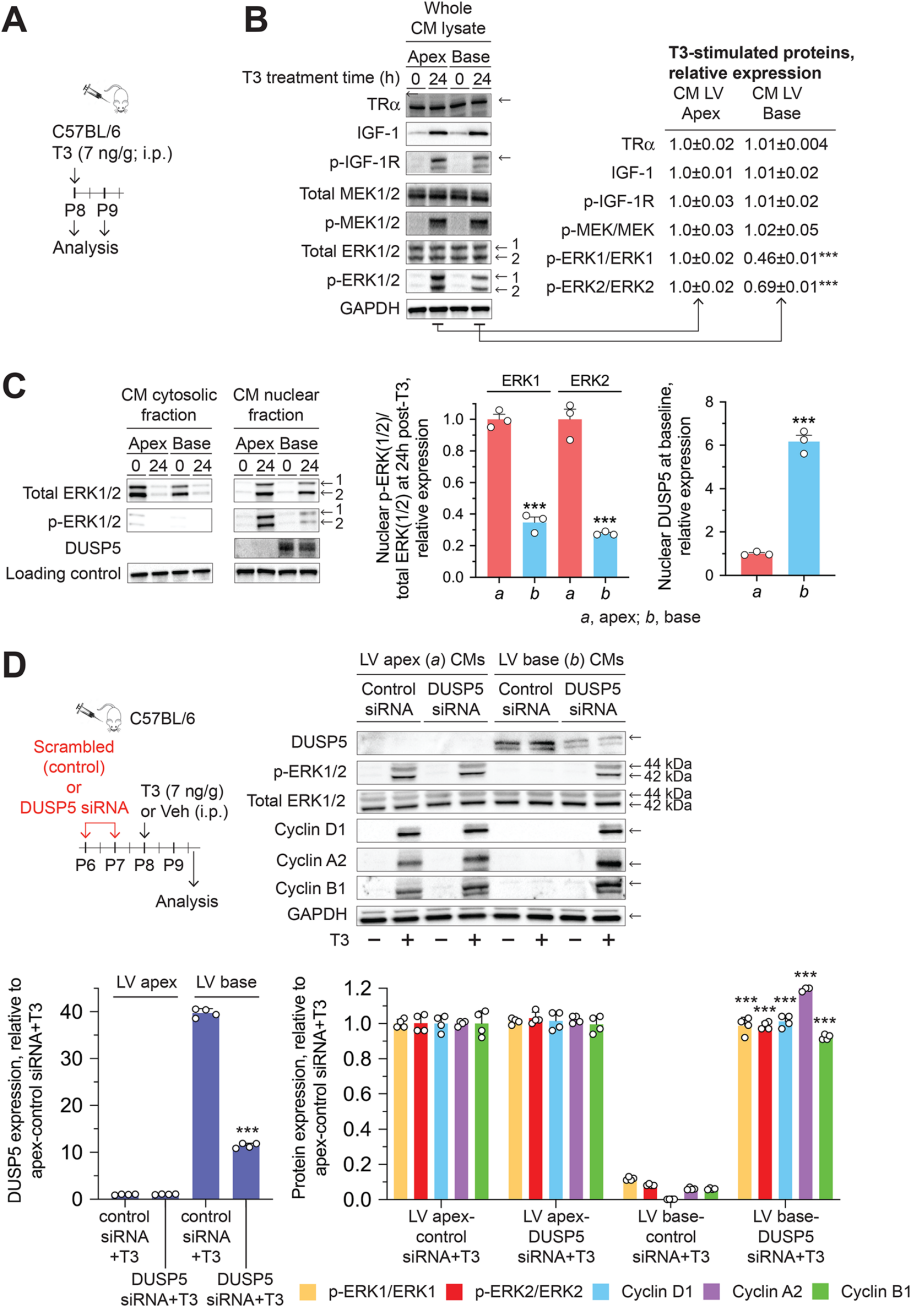
T3-induced ERK1/2 phosphorylation and cyclin expression in cardiomyocytes (CMs) of the P8 LV base is inhibited by DUSP5. (**A**) Schematic showing the protocol for T3 administration and analysis times. (**B**) Representative immunoblots show expression levels of indicated proteins in whole CM lysates obtained from CMs of the LV apex and base at 0 (baseline) and 24 h after T3 administration. Quantitative data relative to that obtained from analysis of CMs of the LV apex, are shown next to the immunoblots. (**C**) Using the same protocol as in A, isolated CMs were resolved into their constituent nuclear and cytosol fractions before immunoblot analysis. Quantitative data are shown in bar graphs adjacent to the immunoblots. In (**B**,**C**) immunoblots are representative of 4 biologically independent replicates. (**D**) DUSP5 or scrambled (control) siRNA was administered intraperitoneally (i.p.) to mice at P6 and P7, followed by T3 ( +) or vehicle (–) treatment at P8. siRNAs were dissolved in the in vivo-jetPEI and 10% glucose mixture. Forty hours later, CMs were isolated and whole cell lysates prepared. Lysates were then subjected to immunoblotting for the indicated proteins. Immunoblots are representative of 4 biologically independent replicates. Quantitative data are presented in the histograms. Data are mean ± SEM. ***P < 0.001 comparisons for intra-LV region derived cardiomyocytes from control versus DUSP5 siRNA treated mice.

Cardiomyocytes of the P8 LV apex and base had similar levels of TRα (Fig. 4A). We administered a single dose of T3 to P8 mice, and isolated cardiomyocytes from the LV apex or base, either immediately after T3 administration (0 h) or after 24 h (Fig. 4A). Furthermore, although T3 increased IGF-1 and IGF-1R (Y1131) and MEK1/2 (S218/S222) phosphorylation similarly, the fraction of total ERK1/2 that was phosphorylated was ~ 50% lower in cardiomyocytes derived from the LV base than in those of the LV apex (Fig. 4B).

Next, we considered the possibility that reduced ERK1/2 phosphorylation in cardiomyocytes of the LV base might impact its cytosol-to-nucleus translocation. We tested this possibility by repeating the experiment in Fig. 4A, but separating the nuclear and cytosol fractions before assessing total ERK1/2 and phosphorylated ERK1/2 levels. T3-stimulated nuclear translocation of ERK1/2 was similar in cardiomyocytes derived from the LV apex and the LV base (Fig. 4C). However, the fraction of nuclear ERK1/2 that was phosphorylated 24 h after T3 treatment was ~ 70% lower in cardiomyocytes of the LV base than in cardiomyocytes of the LV apex (Fig. 4C).

These data suggest differential dephosphorylation of nuclear p-ERK1/2 between LV cardiomyocytes of the apex and base after it is equivalently translocated from the cytoplasm to the nucleus in both regions. We therefore explored the hypothesis that, after exogenous T3 administration, unequal ratios of nuclear p-ERK1/2-to-total ERK1/2 in these LV cardiomyocytes result from differences in p-ERK1/2-targeting phosphatase(s) in the nucleus.

**Figure 5:**
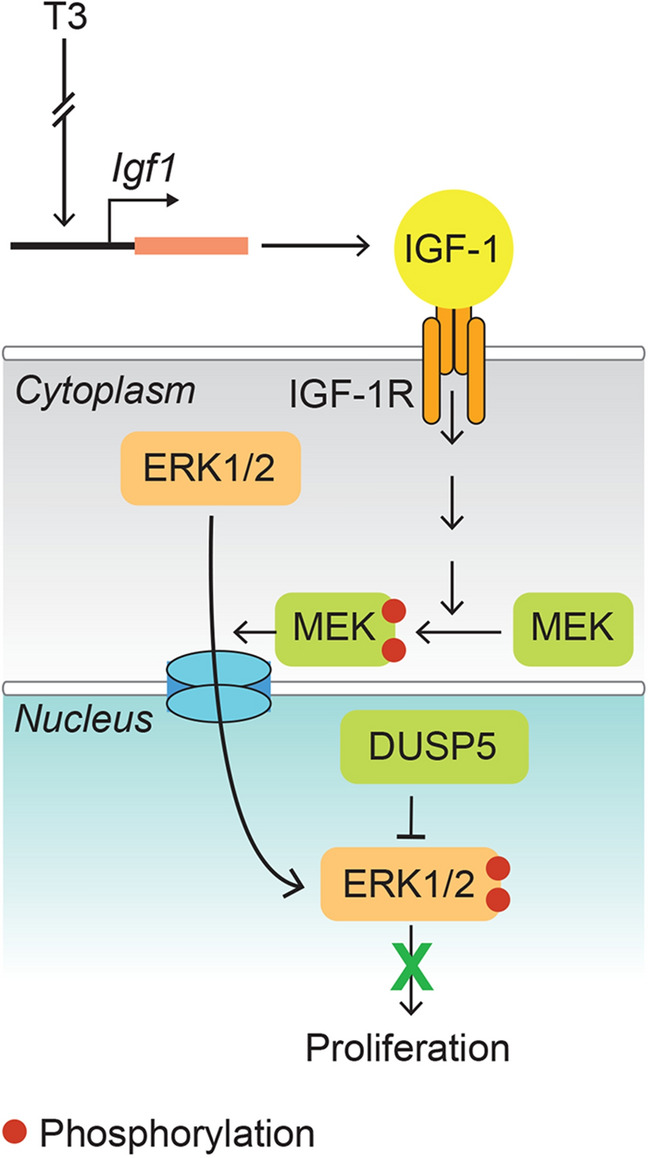
A working model illustrating the role of DUSP5 in inhibiting T3-stimulated increases in nuclear phosphorylated ERK1/2 in cardiomyocytes of the post-P6 LV base.

Dual-specificity phosphatases (DUSPs) are protein tyrosine phosphatases, which dephosphorylate threonine and tyrosine residues on MAPKs. DUSPs are localized to the cytosol, nucleus, or both cell compartments^13^. Of those that are found in the nucleus (DUSPs 1, 2, 4, 5, 8, 10 and 16), DUSP2, 4 and 5 have high reactivity towards p-ERK1/2. Assessment of DUSP2, 4 and 5 mRNA levels in P8 cardiomyocytes derived from the LV base versus the apex revealed a 0.92-fold (*P* = 0.85), 0.85-fold (*P* = 0.39) and 6.3-fold (*P* = 0.008) difference between these LV regions, respectively (*n* = 4/group). Levels of DUSP5 protein were ~ sixfold higher in nuclei of cardiomyocytes derived from the P8 LV base versus the apex (*P* < 0.001) (Fig. 4C). And, in whole cell lysates, DUSP5 levels were ~ fourfold higher in cardiomyocytes than in non-myocytes of the P8 LV base (*P* < 0.01) (Supplementary Fig. S1). We therefore explored the potential involvement of DUSP5, which specifically dephosphorylates nuclear p-ERK1/2^14,15^, in inhibiting T3-stimulated cardiomyocyte proliferative signaling in cells of the LV base of P8 hearts. In vivo treatment of P8 mice with DUSP5-specific siRNA reduced DUSP5 protein expression in cardiomyocytes of the LV base and, in this setting, unmasked T3-mediated stimulation of ERK1/2 phosphorylation and expression of cyclins (Fig. 4D). Collectively, these findings show that DUSP5 inhibits T3-stimulated ERK1/2 phosphorylation in post-P6 cardiomyocytes of the LV base but not apex (Fig. 5).

**Figure 6:**
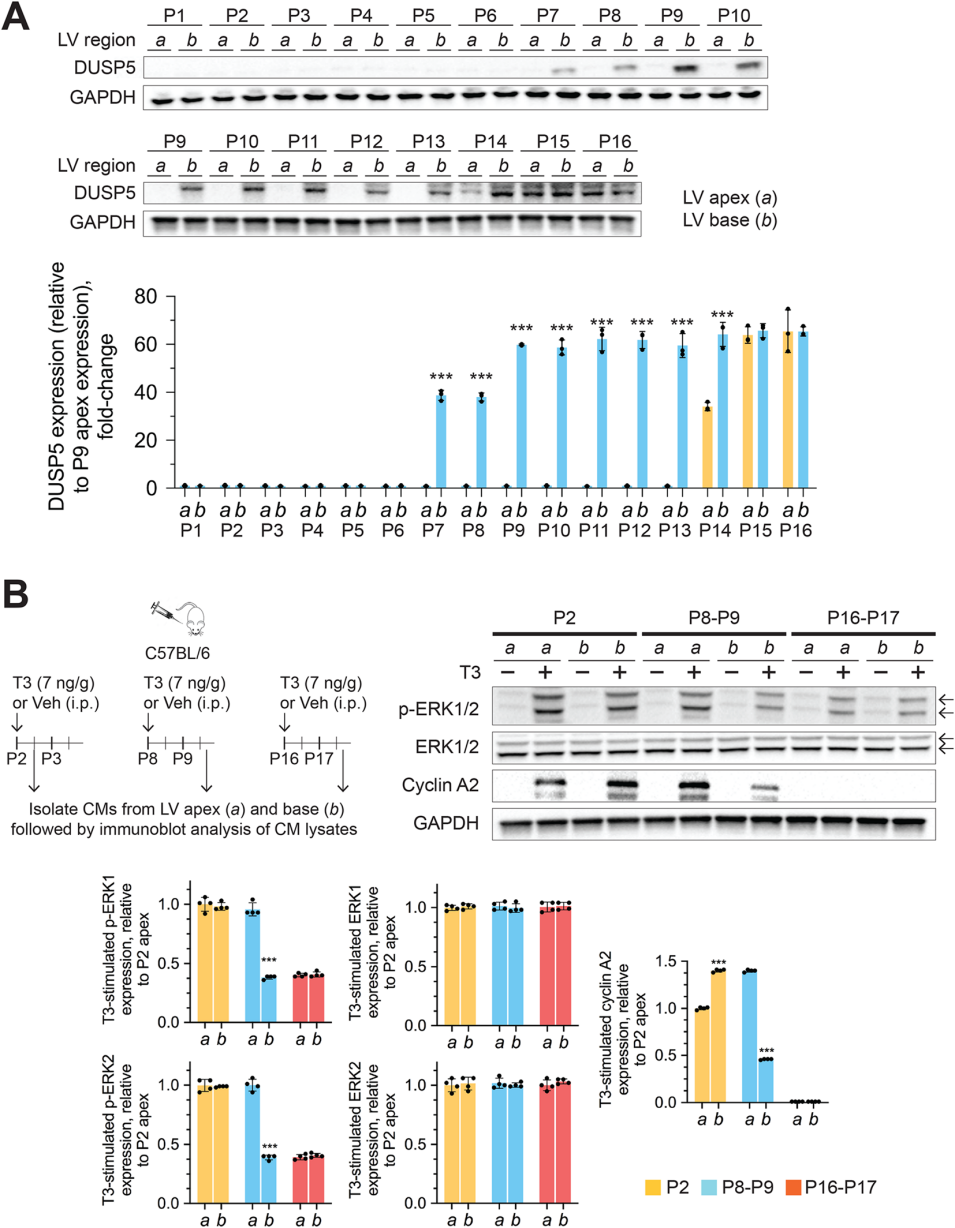
Spatio-temporal changes in LV DUSP5 levels and T3-stimulated ERK1/2 phosphorylation in cardiomyocytes in postnatal mice. (**A**) Representative immunoblots showing developmental changes in myocardial DUSP5 levels in the LV apex (*a*) and base (*b*) of early postnatal hearts. Bar graphs show GAPDH normalized quantitative data relative to DUSP5 levels in apical tissue of the P9 LV myocardium. (**B**) Representative immunoblots showing expression of the indicated proteins in whole cell lysates prepared from cardiomyocytes (CMs) of the LV apex and base of P2 (neonatal), P8 (early preadolescent) and P16 (preadolescent) hearts, both with and without in vivo T3 treatment. The upper and lower arrows indicate the position of ERK1 and 2, respectively. Bar graphs show T3 stimulated, GAPDH normalized, protein expression, relative to expression in P2 LV apex derived CMs. Immunoblots are representative of 3 (A) or 4 (B) biologically independent replicates. Schematics illustrate the experimental protocol. Data are means ± SEM. Comparisons between apex and base LV tissue samples were made using a paired 2-tailed Student’s *t*-test, with *n* = 3 or 4 pairs at each time point.

To determine if the developmental pattern of DUSP5 expression and developmental differences in ERK1/2 activation by exogenous T3 are inversely related, we first examined changes in DUSP5 expression in myocardial tissue derived from the LV apex and base, from birth to P16. DUSP5 was not detectable in the LV apex or base of neonatal animals (Fig. 6A). After P6, DUSP5 expression increased in the LV base and remained high. In the LV apex, DUSP5 expression was first observed at P14; by P15, it was uniformly high throughout the LV. We found that the developmental differences (LV apex versus base), at P2, P8 and P16, in exogenous T3-stimulated ERK1/2 phosphorylation and cyclin A2 expression in cardiomyocytes, (Fig. 6B) were inversely related to developmental expression of DUSP5 in the LV myocardium (Fig. 6A). These findings suggest that before P7 the LV myocardium is uniformly devoid of DUSP5, but after P14 it uniformly expresses DUSP5. Between these developmental ages, DUSP5 levels increase in a spatial and temporal manner, starting at the LV base. This expression is associated with the failure of exogenous T3 to increase p-ERK1/2 and cyclin A2 levels in LV cardiomyocytes.

To determine if ventricular cardiomyocyte proliferation is restrained by DUSP5, we studied the effects of acute DUSP5 depletion in cardiomyocytes on exogenous T3-stimulated cardiomyocyte proliferation. For these experiments, we treated 5-week-old mice with DUSP5 siRNA (2 daily i.p. doses of DUSP5 siRNA) followed by 5 daily doses of either T3 (2 ng/g) or vehicle. Ventricular cardiomyocyte numbers were determined by directly counting ventricular cardiomyocyte numbers after enzymatic disaggregation of cardiac cells. Under these conditions, T3 increased cardiomyocyte numbers by ~ 15% (2.57 ± 0.069 × 10^6^ cardiomyocytes in DUSP5 siRNA + T3-treated mice versus in 2.23 ± 0.029 × 10^6^ cardiomyocytes in DUSP5 siRNA + vehicle-treated mice; *n* = 10/group; *P* < 0.001). But these T3 responses were not observed after scrambled siRNA control therapy (2.16 ± 0.033 × 10^6^ cardiomyocytes in scrambled siRNA + T3-treated mice versus in 2.22 ± 0.041 × 10^6^ cardiomyocytes in scrambled siRNA + vehicle-treated mice; *n* = 10/group; *P* = 0.27). In 5-week-old mice, acute DUSP5 siRNA, but not scrambled siRNA, reduced cardiomyocyte DUSP5 levels by > 99% (*P* < 0.001) (Supplementary Fig. S2). These pharmacological studies suggest that DUSP5 expression in cardiomyocytes inhibits the in vivo proliferative effects of exogenous T3 on these cells.

### DUSP5 regulates hyperplastic heart growth in preadolescent mice

We have previously shown, using propylthiouracil (PTU) to inhibit T3 biosynthesis, that increases in endogenous circulating T3 during early preadolescence stimulate cardiomyocyte proliferation^1^. Could the presence of DUSP5 in these young hearts limit hyperplastic heart growth at this developmental stage? We therefore compared the cardiomyocyte endowment and cardiac morphology of 21-day-old *Dusp5*^*–/–*^ C57BL/6 mice^16^ with age-matched wild-type C57BL/6 mice. *Dusp5*^*–/–*^ mice had similar body weights to wild-type mice, but their ventricular cardiomyocyte numbers were 16% higher (*P* < 0.001) (Table 1). In comparison to wild type mice, *Dusp5*^*–/–*^ mice had 40%–60% thicker LV posterior walls (LVPW), at end-diastole, at the mid-papillary and mid-apical-levels as well as at the apex (*P* < 0.001) (Table 1). The mid-apical level is mid-distance between the mid-papillary level and the LV apex. Values for the mid-papillary and mid-apical levels were derived from short-axis measurements, whereas those for the apex were derived from long-axis measurements. Assessments, at end-diastole, of LV internal dimension (LVID-d) at the mid- papillary level and the mid-apical level indicated that the LV chamber of *Dusp5*^*–/–*^ mice was narrower (by ~ 11% at both the mid papillary and mid-apical levels; the respective *P*-values were 0.005 and 0.011) and longer (LVID-d-long axis) (by 5%, *P* = 0.009) (Table 1). In these P21 *Dusp5*^*–/–*^ hearts, LV fractional shortening was ~ 8 points higher (*P* < 0.001) with respect to the short axis and ~ 5-points higher with respect to the long axis (*P* = 0.011) (Table 1), and ejection fraction (determined by B-mode echocardiography) was 13 points higher (*P* < 0.001), suggesting better LV contractile function relative to wild-type mice. Collectively, these findings suggest that DUSP5 expression restrains hyperplastic LV growth during early preadolescence.

**Table 1:** Cardiomyocyte numbers, body weights, LV mass and dimensions and LV contractile function of 21-day-old C57BL/6 wild-type and *Dusp5*^*–/–*^ Mice.

	Wild-type	*Dusp5*^*–/–*^	*P*-value
Mice, *n* [M/F]	8 [6/2]	8 [4/4]	
Body weight, g	11.4 ± 0.54	10.7 ± 0.36	0.36
LV mass, mg	39.1 ± 1.6	52.5 ± 1.7	< 0.001
**Cardiomyocyte**			
Numbers, (millions)	2.36 ± 0.045	2.73 ± 0.065	< 0.001
**LV short-axis parameters**			
*LV mid-papillary level*			
LVPW-d, mm	0.39 ± 0.018	0.54 ± 0.012	< 0.001
LVPW-s, mm	0.86 ± 0.038	0.92 ± 0.009	0.15
LVID-d, mm	3.22 ± 0.06	2.87 ± 0.087	0.005
LVID-s, mm	2.39 ± 0.049	1.88 ± 0.056	< 0.001
Fractional shortening, %	25.9 ± 1	34.2 ± 1.24	< 0.001
*LV mid-apical level*			
LVPW-d, mm	0.34 ± 0.013	0.53 ± 0.017	< 0.001
LVPW-s, mm	0.85 ± 0.035	0.93 ± 0.016	0.048
LVID-d, mm	2.71 ± 0.062	2.4 ± 0.087	0.011
LVID-s, mm	1.88 ± 0.049	1.47 ± 0.036	< 0.001
Fractional shortening, %	30.5 ± 0.94	38.5 ± 1.8	0.0013
**LV long-axis parameters**			
LVPW-d, mm	0.34 ± 0.014	0.54 ± 0.018	< 0.001
LVPW-s, mm	0.85 ± 0.032	0.93 ± 0.016	0.038
LVID-d, mm	4.29 ± 0.058	4.52 ± 0.052	0.0085
LVID-s, mm	3.49 ± 0.066	3.46 ± 0.047	0.47
Fractional shortening, %	18.5 ± 1.4	23.5 ± 1	0.011
LV volume at diastole, µl	42.1 ± 2.1	34.5 ± 2.2	0.026
LV volume at systole, µl	20 ± 1.1	11.8 ± 0.78	< 0.001
Ejection fraction, %	52.4 ± 1.4	65.5 ± 1.6	< 0.001
Stroke volume, µl	22.1 ± 1.37	22.7 ± 1.7	0.79

*C57BL/6 and *Dusp5*^*–/–*^ mouse litters were adjusted at P2 to 4 pups/dam). Transthoracic echocardiography, M- and B-modes, were performed in 21-day-old mice under light isoflurane anesthesia. Immediately following this procedure, cardiomyocyte numbers were determined in whole-ventricle cell-suspensions of disaggregated hearts using a hemocytometer. Cardiomyocytes were identified by their shape and size. Data were analyzed by unpaired 2-tailed Students *t*-test. Values are mean ± SEM. *LV* left ventricle, *LVPWD-d* LV posterior wall dimension at end-diastole, *LVPWD-s* LV posterior wall dimension at end-systole, *LVID-d* LV internal dimension at end-diastole, *LVID-s* LV internal dimension at end-systole.

## Discussion

Previously, we have shown that exogenous T3 stimulates cardiomyocyte proliferation in P2 murine hearts. These cardiomyocytes are immature and are capable of proliferating in response to growth factors and injury^12,17,18^. After P6, however, cardiomyocytes do not proliferate in response to injury^18^ and are resistant to growth factor-induced cell proliferation^17^. Identification of factors that stimulate proliferation in vivo in these post-neonatal cardiac muscle cells may also allow the regeneration of myocardium in injured adult hearts^19^.

We show here that exogenous T3 stimulates in vivo cardiomyocyte proliferation in P8 hearts. The robustness of the T3 proliferative response is evident from careful direct quantitation of cardiomyocyte numbers, which showed an ~ 18% increase (*P* < 0.001) in these cardiac muscle cells. The proliferative effects are, however, restricted to cardiomyocytes of the LV apex. This conclusion is supported by the findings that, in response to exogenous T3, mitosis (as assayed by pH3-labeling) and cell replication (as assessed by genetic lineage-tracing studies with *Myh6*-MerCreMer::Rosa26fs-Confetti mice) is stimulated only in cardiomyocytes of the LV apex.

We investigated why only a subset of cardiomyocytes, residing at the P8 LV apex, proliferate in response to exogenous T3. Prior studies in neonatal murine cardiomyocytes have shown that T3 proliferative signaling is mediated by increases in IGF-1 and IGF-1R expression^12^. This expression increases growth factor signaling, which phosphorylates and thereby activates the MAP kinases ERK1 and 2. Increases in nuclear p-ERK1/2 are required for growth factor-induced cardiomyocyte proliferation, including that due to T3^12^. The rather restricted action of T3 in P8 LV cardiomyocytes is different from the in vivo actions of T3 in neonatal P2 hearts where T3 increases proliferative ERK1/2 signaling in LV cardiomyocytes irrespective of their regional location (Fig. 6B). We therefore investigated if T3-stimulated ERK1/2 signaling in P8 LV cardiomyocytes is dependent on their spatial location. The T3 proliferative effect is mediated through TRα^12^. We found that expression of this receptor, as well as the ability of T3 to stimulate downstream effectors, viz., IGF-1 expression, IGF-1R activation and MEK1/2 phosphorylation (which are MAP kinases that phosphorylate ERK1/2), occur to a similar extent in cardiomyocytes of both the LV apex and base. However, the ability of T3 to increase levels of p-ERK1/2 was compromised in cardiomyocytes of the LV base; this despite the fact that ERK1/2 almost completely translocates to the nucleus upon T3 stimulation. Because nuclear ERK1/2 phosphorylation levels need to be maintained to activate downstream transcriptional responses, we postulated that p-ERK1/2 dephosphorylation might be enhanced in cardiomyocytes of the P8 LV base. Through systematic analyses, we identified a sixfold increase in the level of a p-ERK1/2-specific nuclear phosphatase, DUSP5, in cardiomyocytes of the P8 LV base versus those of the apex. To establish causality, we studied the effect of DUSP5 suppression on T3 stimulated cardiomyocyte p-ERK1/2. We found that in vivo depletion of DUSP5 in cardiomyocytes of the P8 LV base, using a cocktail of DUSP5-targeting siRNAs, increased T3-stimulated p-ERK1/2 levels to those observed in cardiomyocytes of the LV apex.

A study of the developmental expression of DUSP5 in young murine hearts indicates that the expression of this phosphatase spreads progressively from LV base at P7 to the apex by P16. Before P7, LV cardiomyocytes do not express DUSP5. Consistent with this pattern of expression, in vivo ERK1/2 phosphorylation in P8 LV cardiomyocytes, in response to exogenous T3 administration, was spatially inversely related to DUSP5 expression. These findings, together with our in vivo findings on the administration of exogenous T3 in the presence or absence of DUSP5 siRNA, led us to conclude that spatial differences in T3-stimulated nuclear p-ERK1/2 accumulation in P8 LV cardiomyocytes are caused by asymmetric expression of DUSP5.

Next, we considered the possibility that DUSP5 suppression might be useful in expanding cardiomyocyte numbers in adult heart. We found that exogenous T3 increases ventricular cardiomyocyte numbers in young adult mice after acute in vivo depletion of DUSP5 using DUSP5 siRNA, but not after scrambled siRNA treatment. We were unable to evaluate if this in vivo T3 effect on cardiomyocyte proliferation is direct because of technical limitations in culturing adult cardiomyocytes. In our hands, adult cardiomyocytes required serum-containing media for viability (data not shown). While the T3 proliferative effect is readily seen using neonatal cardiomyocytes cultured in serum-free media^12^, its proliferative effects are diminished when cardiomyocytes are kept in serum-containing media; presumably, this is because the T3 proliferative effect is mediated by growth factor secretion (i.e., IGF-1), the effects of which are likely to be masked by the abundance of growth factors present in serum. While our inability to show that T3 + DUSP5 siRNA combination stimulates cardiomyocyte proliferation in vitro is a limitation, it does not detract from our finding that a combination of these molecules can increase cardiomyocyte numbers in young adult hearts.

Detailed changes in LV morphology, resulting from significant levels of cardiomyocyte hyperplasia, have been reported infrequently. Using transthoracic echocardiography, we show that cardiomyocyte hyperplasia in *Dusp5*^–/–^ mice is associated with an altered cardiac morphology as a result of an increase in LV mass and LV wall thickness that narrows LV chamber dimensions and improve LV contractile function, as evident from increases in LV fractional shortening and LV ejection fraction. Importantly, in contrast to other genetic models of enhanced cardiomyocyte proliferative growth^17,20^, the morphological changes observed here during development in *Dusp5*^*–/–*^ mice do not impact negatively on stroke volume, body weight or survival.

Hirose et al.^6^ propose that endogenous T3 causes cell cycle arrest in postnatal cardiomyocytes, which contrasts with our conclusions. Their conclusion is primarily based on studies with a genetic mouse model in which a TRα mutation is introduced in cardiomyocytes during embryonic development. In this mutant mouse, in which the affinity of TRα for T3 is reduced, postnatal cell cycle exit is delayed. The structure of TR loci and signaling are complex; strikingly opposing phenotypes could result depending on the TR mutation^21^, developmental stage and cellular context^22^. These phenotypes are also highly dependent on the impact of the individual TR mutations on compensatory changes in the expression of other TR subtypes^23–26^ as well as the consequence of the mutation on the resultant apo-receptor, which regulates extensive gene repression in late fetal heart development. These potential phenotypic effects of the TRα mutation could be a source of discrepant conclusions between Hirose et al.^6^ and the current studies.

Our studies provide evidence for inhomogeneity of LV cardiomyocytes during early postnatal heart development that underlies spatial differences in mitogenic responsiveness to T3. Exploiting the mechanism described here may prove useful in expanding the number of cardiomyocytes in injured adult hearts.

## Materials and methods

A detailed description of the experimental procedures related to cardiomyocyte isolation for immunocytochemistry, RT-qPCR and immunoblotting is provided in the Supplementary Information section.

### Animal husbandry and mouse models

All animal studies were approved by the Institutional Animal Care and Use Committee (IACUC) of Emory University. We confirm that all experiments were performed in accordance with IACUC guidelines and regulations. Adult C57BL/6 mice were used for breeding. Conditions for animal husbandry protocol are adapted from our earlier studies^12^. Briefly, the mothers were between 3–8-months-old. Mice were allowed food and water ad libitum. An animal husbandry protocol was developed to minimize variation between litters and between studies. Factors considered were as follows: (1) age of the mother (only dams less than 36 weeks were used); (2) animal chow (we used a 50/50 mix of Purina Lab Diets Rodent diet 5001 (standard diet) and 5015 (breeder diet); (3) minimization of stress on the dams, especially between the first 2 days after delivery and weaning; (4) standardization of litter sizes (only pups from litter sizes of 6–8 (at P2) were used); (5) litter sizes from these births were further adjusted to 4 pups per dam at P2 to minimize inter-experiment variation in growth; (6) to minimize intra-litter variation, drugs were given to about one half of the pups in a litter and vehicle to the rest; and (7) separation of plugged/pregnant dams into individual cages. C57BL/6 wild type (Jackson Laboratory, 000664) male and female mice were used for these studies. Wherever possible we constituted each adjusted litter with equal numbers of male and female mice.

For cardiomyocyte lineage tracing studies, we used Rosa26fs-Confetti [B6.129P2-Gt(ROSA)26Sortm1(CAG-Brainbow2.1)Cle/J, Jackson Laboratory, 017492] and *Myh6*-MerCreMer [B6.FVB(129)-A1cfTg(*Myh6*-cre/Esr1*)1Jmk/J, Jackson Laboratory, 005657] mice. We then generated double-transgenic Myh6-MerCreMer::Rosa26fs-Confetti mice by breeding. In the double transgenic mice, Cre recombinase causes the Brainbow 2.1 construct to recombine, which randomly labels cardiomyocytes either with GFP, CFP, RFP or YFP. We limited the extent of Cre-mediated recombination by adjusting the dose of 4-hydroxytamoxifen (Sigma-Aldrich, H7904-5 mg) to minimize replication-independent occurrences of adjacent cardiomyocytes of the same color. For studying the biological role of DUSP5 in postnatal heart development we used *Dusp5*^*–/–*^ mice on the C57BL/6 background^16^.

Drugs such as T3 and 4-hydroxytamoxifen were administered, intraperitoneally (i.p.), to each mouse at the dose indicated. Propylthiouracil (PTU, 0.15%) was administered in drinking water to inhibit T3 biosynthesis in mice from P7 to P13 using the protocol from our earlier studies^1^. Vehicle was administered by the same route and animals thus treated served as controls. Phosphate buffered saline (PBS) was the vehicle for T3 and soybean oil was the vehicle for 4-hydroxytamoxifen. DUSP5-specific siRNA or scrambled siRNA (control) was administered using in vivo-jetPEI, (VWR, 89129–960). DUSP5 siRNA (100 µg) was dissolved in 1 ml of the in vivo-jetPEI:10% glucose mixture and was injected 100 µl per mouse via i.p. route (10 µg/mouse). DUSP5 siRNA (sc-60555) was a pool of 2 different siRNA duplexes (sc-60555A, sense: CAUGGCUUACCUCAUGAAtt and antisense: UUCAUGAGGUAAGCCAUGCtt; sc-60555B, sense: GACAGCUCCUUCAGUAUGAtt and antisense: UCAUACUGAAGGAG-CUGUCtt). All sequences are provided in 5′ → 3′ orientation. We did not use any litters if one or more mouse in the litter was observed to be sick or stunted. P7 and older mice were first anesthetized with 5% isoflurane and then hearts were harvested for further processing. Hearts were also collected at 0, 24 and 40 h after T3 or vehicle treatment and ventricular cardiomyocytes were prepared from the apex and base for immunoblotting, RT-qPCR or immunocytochemistry. In addition, hearts were collected at various ages after birth for immunoblotting, immunocytochemistry, immunohistochemistry and cardiomyocyte number estimation.

### Echocardiography analysis

Transthoracic echocardiography was performed under light isoflurane anesthesia on either wildtype controls or *Dusp5*^*–/–*^ mice at P21 using the Vevo 3100 imaging platform (VisualSonics) with a MX550D linear array transducer (axial resolution: 40 µm) and the images were analyzed using Vevo Lab Desktop Software. The automated zoom function of the Vevo 3100 imaging platform allows precise quantification. Both B-mode and M-mode measurements were taken to assess the cardiac morphology and function. Parasternal short axis M-mode echocardiographic imaging was used to determine wall thicknesses and internal diameter of the LV at end-diastole and end-systole; fractional shortening was derived from these parameters. IVS function of M-mode (minor axis) of Vevo Lab Desktop Software was used to determine these parameters. Endocardium and epicardium were outlined using the IVS function by sequential click-defining the anterior epicardial, endocardial and then posterior endocardial and epicardial wall. These measurements were made at the mid-apical level (half way between the apex and the LV mid papillary level; ~ 1 mm from the apex) or mid-papillary level (~ 3.5 mm from the apex). Additionally, B-mode parasternal long axis images were acquired to determine LV major axis internal diameters and wall thicknesses. Using B-mode (major axis), length of the LV chamber was determined by measuring distance from the aortic root to the apical endocardial wall at end-systole and end-diastole. We also measured LVPW thickness at the apex using B-mode (long-axis) by sequential click-defining apical wall from the epicardium to endocardium. LV volumes at end-diastole and end-systole were also determined using B-mode parasternal long axis images of the heart. LV ejection fraction measurements were calculated using the LV-trace function of the Vevo Lab Desktop Software in B-mode. LV endocardial borders were traced at end-diastole and end-systole by sequential click-defining the aortic root, apex, anterior wall, posterior wall and repeat click-defining of anterior and posterior wall until the myocardial wall is outlined. The Vevo Lab Desktop Software automatically determines volumes at end-diastole, end-systole, and calculates LV volume, SV and LV ejection fraction. At least three different images were taken for each cardiac parameter and measurements from these three individual images were averaged to acquire final measurement for that cardiac parameter.

### Cardiomyocyte number determination

Protocol for cardiomyocyte number determination is customized based on our earlier studies^12^. Briefly, heparin (100–200 µl, 1000 USP units/ml) was injected intraperitoneally 8 min prior to harvesting. Hearts were harvested under deep anesthesia using 5% isoflurane. Hearts with their atria and aorta attached were washed with PBS and then the aorta cannulated for retrograde perfusion through the coronary circulation. Hearts were immediately perfused with cytofix (BD Biosciences, 554655) for 1 min. Subsequently, hearts were perfused with perfusion buffer (120 mmol/L NaCl, 15 mmol/L KCl, 0.5 mmol/L KH_2_PO_4_, 5 mmol/L NaHCO_3_, 10 mmol/L HEPES, and 5 mmol/L glucose, at pH 7.0) for 2 min and then with perfusion buffer containing collagenase type 2 (Worthington, LS004176) for 8–15 min at 37 °C. Perfusion and digestion buffers were freshly prepared, warmed to 37 °C and aerated with 5% CO_2_. Collagenase concentration was 1 mg/ml for P8 to P12 hearts and 2 mg/ml for P16 or older hearts. After 8–15 min of digestion, the atria were excised and the cardiac ventricles were placed in a 6 cm dish containing 2 ml of digestion buffer; we then added ~ 2 ml of STOP buffer (perfusion buffer plus 10% bovine calf serum and 12.5 mmol/L CaCl_2_). The ventricles were teased apart into small pieces followed by trituration through pipettes of progressively smaller diameters. The digested cardiomyocytes from each heart were collected in a 15 ml falcon tube and more STOP buffer was added to a volume of 10 ml. The final cell suspension was used to count cardiomyocytes using a hemocytometer. To avoid losses, cardiomyocytes were not purified, but could be readily identified by phase contrast microscopy based on their cytoplasmic size and rod shape. Four aliquots were counted per heart and the mean value was used to determine the total number of ventricular cardiomyocytes per heart.

For accurate cardiomyocyte number determination, a critical step is optimal digestion efficiency and operator-specific variability in isolation and counting. We have eliminated operator-specific variability by using the same operator between experiments. Heart digestion is chiefly dependent on collagenase concentration in the perfusion medium, its activity, exposure time and temperature. To optimize digestion efficiencies, these variables were adjusted for each group of mice depending on the age of the mouse. Importantly, collagenase activity was kept uniform between the biological replicates and across experiments by using the same lot of enzyme. Additionally, a brief fixation with cytofix (~ 1 min) before starting perfusion with collagenase helps in protecting individual cardiomyocyte structure and prevents generation of fragmented cardiomyocytes. Cardiomyocytes during the postnatal stages analyzed are much larger than non-myocytes and are readily identifiable due to their cytoplasmic size and rod-shape^12^. Digestion efficiency was calculated [ventricular weight %, determined by (original weight – residual)/original weight] after each change in condition. We found that maximal digestion efficiencies were between ~ 97 and 99%. Upon microscopic examination, the residual tissue was almost entirely undigested cardiac valves and blood vessels. Over-digestion neither improved digestion efficiencies, nor did it increase cardiomyocyte yield. We did not estimate cardiomyocyte numbers from under-digested hearts in which disaggregation of myocardial tissue was incomplete. Suboptimal cannulation of the aorta was the cause of under-digestion but was infrequent.

### Immunofluorescence

Cardiomyocytes were isolated as described above and fixed in Cytofix (BD Biosciences, 554,655) for 5 min. After pre-blocking, cardiomyocytes were stained with anti-cardiac troponin T- (Miltenyi Biotec, 130-119-674), or anti-phospho-histone H3-AlexaFluor 594 conjugate (Cell Signaling Technology, 8481S) in 10% v/v goat serum. EdU positive cardiomyocyte nuclei were detected using Click-iT EdU Alexa Fluor 594 Imaging Kit (Thermo Fisher Scientific, C10339). DAPI was used to stain cardiomyocyte nuclei. Images were acquired on a confocal microscope (Leica SP5).

### Statistical analysis

Statistical significance of data was determined using Prism 8-GraphPad. The Shapiro–Wilk test was used to determine if the data were normally distributed; in this case, we used one-way ANOVA followed by Tukey multiple comparisons test, or unpaired two-tailed Student’s *t*-test for comparisons involving 2 groups, or paired *t*-test for intra-cardiac comparison of the apex with the base of the same heart. For estimation of variance, the F-test was used when comparing 2 groups and the Brown-Forsythe test was used when comparing multiple groups by 1-way ANOVA. *P*-values < 0.05 were considered significant. Results are expressed as mean ± SEM or as Tukey’s box and whisker plots.

## Supplementary Information

Supplementary Information

## Data Availability

The data and resources generated for this manuscript are available upon reasonable request from the corresponding authors.

## References

[1] Naqvi N (2014). A proliferative burst during preadolescence establishes the final cardiomyocyte number. Cell.

[2] Krenz M, Robbins J (2004). Impact of beta-myosin heavy chain expression on cardiac function during stress. J. Am. Coll. Cardiol..

[3] Bai SL, Campbell SE, Moore JA, Morales MC, Gerdes AM (1990). Influence of age, growth, and sex on cardiac myocyte size and number in rats. Anat. Rec..

[4] Li F, Wang X, Capasso JM, Gerdes AM (1996). Rapid transition of cardiac myocytes from hyperplasia to hypertrophy during postnatal development. J. Mol. Cell Cardiol..

[5] Alkass K (2015). No evidence for cardiomyocyte number expansion in preadolescent mice. Cell.

[6] Hirose K (2019). Evidence for hormonal control of heart regenerative capacity during endothermy acquisition. Science.

[7] Xing W (2012). Genetic evidence that thyroid hormone is indispensable for prepubertal insulin-like growth factor-I expression and bone acquisition in mice. J. Bone Miner. Res..

[8] Snippert HJ (2010). Intestinal crypt homeostasis results from neutral competition between symmetrically dividing Lgr5 stem cells. Cell.

[9] Chang L, Karin M (2001). Mammalian MAP kinase signalling cascades. Nature.

[10] Mebratu Y, Tesfaigzi Y (2009). How ERK1/2 activation controls cell proliferation and cell death: Is subcellular localization the answer?. Cell Cycle.

[11] Oberkotter LV, Rasmãoessen KM (1991). Changes in plasma thyroid hormone concentrations in chronically food-restricted female rats and their offspring during suckling. J. Nutr..

[12] Tan L (2019). Redox activation of JNK2α2 mediates thyroid hormone-stimulated proliferation of neonatal murine cardiomyocytes. Sci. Rep..

[13] Jeffrey KL, Camps M, Rommel C, Mackay CR (2007). Targeting dual-specificity phosphatases: Manipulating MAP kinase signalling and immune responses. Nat. Rev. Drug Discov..

[14] Keyse SM (2008). Dual-specificity MAP kinase phosphatases (MKPs) and cancer. Cancer Metastasis Rev..

[15] Caunt CJ, Keyse SM (2013). Dual-specificity MAP kinase phosphatases (MKPs): shaping the outcome of MAP kinase signalling. FEBS J..

[16] Kutty RG (2016). Dual specificity phosphatase 5 is essential for T cell survival. PLoS ONE.

[17] D'Uva G (2015). ERBB2 triggers mammalian heart regeneration by promoting cardiomyocyte dedifferentiation and proliferation. Nat. Cell Biol..

[18] Porrello ER (2011). Transient regenerative potential of the neonatal mouse heart. Science.

[19] Foglia MJ, Poss KD (2016). Building and re-building the heart by cardiomyocyte proliferation. Development.

[20] Chaudhry HW (2004). Cyclin A2 mediates cardiomyocyte mitosis in the postmitotic myocardium. J. Biol. Chem..

[21] Flamant F, Samarut J (2003). Thyroid hormone receptors: Lessons from knockout and knock-in mutant mice. Trends Endocrinol. Metab..

[22] Puzianowska-Kuznicka M, Pietrzak M, Turowska O, Nauman A (2006). Thyroid hormones and their receptors in the regulation of cell proliferation. Acta Biochim. Pol..

[23] Chatonnet F, Guyot R, Benoit G, Flamant F (2013). Genome-wide analysis of thyroid hormone receptors shared and specific functions in neural cells. Proc. Natl. Acad. Sci. USA.

[24] Ortiga-Carvalho TM, Sidhaye AR, Wondisford FE (2014). Thyroid hormone receptors and resistance to thyroid hormone disorders. Nat. Rev. Endocrinol..

[25] Kaneshige M (2001). A targeted dominant negative mutation of the thyroid hormone α1 receptor causes increased mortality, infertility, and dwarfism in mice. Proc. Natl. Acad. Sci. USA.

[26] Forrest D, Vennström B (2000). Functions of thyroid hormone receptors in mice. Thyroid.

