# Supplementary material for: DUSP5 expression in left ventricular cardiomyocytes of young hearts regulates thyroid hormone (T3)-induced proliferative ERK1/2 signaling
Source: Sci Rep. 2020 Dec 14;10:21918. doi: 10.1038/s41598-020-78825-x (PMC7736286; doi:10.1038/s41598-020-78825-x)

DUSP5 expression in left ventricular cardiomyocytes of young hearts regulates thyroid hormone (T3)-induced proliferative ERK1/2 signaling

Nikolay Bogush[1,†], Lin Tan[1,†], Hussain Naib[1], Ebrahim Faizullabhoy[1], John W. Calvert[2], Siiri E. Iismaa[3], Ankan Gupta[4], Ramani Ramchandran[4], David I.K. Martin[5], Robert M. Graham[3], Ahsan Husain[1,]* and Nawazish Naqvi[1,]*

[1]Department of Medicine (Cardiology), Emory University School of Medicine, Atlanta, GA, USA. [2]Department of Surgery, Carlyle Fraser Heart Center, Emory University School of Medicine, Atlanta, GA, USA. [3]Victor Chang Cardiac Research Institute, Darlinghurst, New South Wales, Australia. [4]Developmental Vascular Biology Program, Division of Neonatology, Department of Pediatrics, Department of Obstetrics and Gynecology, Children's Research Institute, Medical College of Wisconsin, Milwaukee, WI, USA. [5]Children's Hospital Oakland Research Institute, Oakland, CA, USA.

[†]These authors contributed equally: Nikolay Bogush, Lin Tan.

*Corresponding Authors: Nawazish Naqvi, PhD, Department of Medicine (Cardiology), Emory University, 323 WMRB, 101 Woodruff Circle, Atlanta Georgia 30322. E-mail nnaqvi@emory.edu or Ahsan Husain, PhD, Department of Medicine (Cardiology), Emory University, 3311 WMRB, 101 Woodruff Circle, Atlanta Georgia 30322. Tel: 404-727-8125; E-mail ahusai2@emory.edu

Short title: Spatial heterogeneity of T3-proliferative signaling in young hearts.

Key words: Thyroid hormone, cell proliferation, DUSP5, ERK1/2 signaling, IGF-1/IGF-1R signaling, genetic lineage tracing.

Supplementary Section includes Supplementary Methods, a Supplementary Table and 2 Supplementary Figures.

Supplementary Methods

Cardiomyocyte isolation for immunocytochemistry and immunoblotting

For immunoblotting and immunocytochemistry, hearts were enzymatically digested as described in *Materials and Methods* using procedures described earlier[12]. Before making single cell suspensions, atria were excised, and the LVs were divided into apex, mid and base regions, as described below. Cardiac cells of the apex and base were separately disaggregated into single cell suspension. Cardiomyocytes were purified with three low speed centrifugations (18 x g for 4 min at room temperature), which pelleted cardiomyocytes. Supernatant fractions, enriched in non-myocytes, were discarded. The resulting cardiomyocyte preparations were >95% pure. Aliquots of cardiomyocytes were snap frozen in liquid nitrogen and stored at –80°C for immunoblotting. Additionally, aliquots were fixed with Cytofix (BD Biosciences, 554655) for five min and spread on glass slides for immunocytochemistry.

RT-qPCR

The RT-qPCR protocol used was customized from our earlier studies[12]. Briefly, cardiomyocytes in tubes containing RNAlater stabilization solution at –80 °C, were thawed on ice and then centrifuged at 21,000 x g for 10 min. The RNAlater supernatant fraction was removed and replaced with 240 µl of lysis binding-buffer from the mirVana miRNA Isolation Kit (Thermo Fisher Scientific, AM1560). RNA was purified according to the manufacturer's guidelines. Purified RNA was reverse transcribed using Transcriptor Reverse Transcriptase (Roche, 03531295001) and random primer (Primer, random p(dN)6, Roche, 11034731001). Quantitative PCR was performed with SYBR Green Supermix (Bio-Rad, 1708882) on a iQ5 Thermal Cycler (Bio-Rad). Respectively, the 5' and 3' PCR primers used were: Dusp2: TTCTTGCGAGGCGGT-TTCAA and AGTTG-CTATTTTCGGCCCCA; Dusp4: GAGAGTCTCCGGGAGGACAG and

GTCCTTTACTGCGTCGATGT; and Dusp5: TCGCCTACAGACCAGCCTAT and GTAGT-GTAGGTGGGTGGTGC.

Immunoblotting

The immunoblotting protocol used was customized from our earlier studies[12]. Briefly, whole cell cardiomyocyte lysates were generated by re-suspending cardiomyocytes in 250 µl of RIPA buffer (Cell Signaling, 9806S) supplemented with phosphatase inhibitor cocktail 2 and 3 (Sigma-Aldrich, P5726-1ML and P0044-1ML), 0.1 mmol/L phenylmethylsulfonyl fluoride (PMSF, Sigma-Aldrich, 93482-50ML-F) and protease inhibitor cocktail (Roche, 11697498001); cardiomyocytes were lysed by sonication and then pelleted by centrifugation at 21,000 x g for 30 min. The resulting supernatant fractions were aliquoted into fresh Eppendorf tubes and then snap-frozen in liquid nitrogen. Aliquots, stored at –80 °C, were allowed to thaw on ice immediately before use. Initially, a 5 to 10 µl aliquot (~20 µg protein) was mixed with an equal volume of 2x Laemmli sample buffer (Bio-Rad, 1610737), heated for 5 min at 95–99 °C and then immediately cooled on ice for 5 min. The samples were then centrifuged briefly before fractionation by SDS–polyacrylamide gel (12–18%) electrophoresis (SDS-PAGE), which was performed at 200 volts for 5 min, and then at 150 volts for 30 min to 2 h. The resolved proteins were then transferred to a PVDF membrane by electroblotting (Turbo Transfer; Bio-Rad). Depending upon the molecular weight of the proteins or protein complexes, the transfer time on Turbo Transfer was varied for high (11 min), average (7 min) and low (5 min) molecular weight proteins. After transfer, all blots were pre-blocked for 30–60 min with Superblock (Thermo Fisher Scientific, 37536). Initially, the samples were probed with a GAPDH antibody. Based on the intensity of GAPDH, the volume of each sample loaded was adjusted to normalize the amount of GAPDH per sample. Membranes were probed with the target protein-specific primary

antibody. For quantitative analysis, the membrane was then stripped and re-probed with GAPDH to ensure that loading was normalize for each sample. For stripping, the membrane was washed twice with

1x Tris-buffered saline (TBS, Thermo Fisher Scientific, BP2471-1) five min each and then incubated with Restore Western Blot Stripping Buffer (Thermo Fisher Scientific, 21059) for 5–15 min and then washed again twice with 1x TBS and pre-blocked with Superblock (Thermo Fisher Scientific, 37536) for 1 h before incubating with GAPDH antibody. Primary antibodies (see below) were also diluted in Superblock and incubated with the membranes for 2 h at 22 °C, or overnight at 4 °C, followed by horseradish peroxidase (HRP)-labeled secondary antibody (1:10,000) for 45 min at 22 °C. The signals were detected using Super Signal West Dura Detection Reagent (Thermo Fisher Scientific, 34075) and images captured on a Bio-Rad GelDoc system equipped with a CCD camera. Quantification was performed by densitometry using the ImageLab program (Bio-Rad).

For studies involving nuclear protein quantification, nuclear and cytoplasmic fractions were separated from cardiomyocytes using NE-PER Nuclear Cytoplasmic Kit (Thermo Fisher Scientific, 78833) as per the manufacturer's protocol. Nuclear and cytoplasmic fractions were then similarly resuspended in RIPA buffer and stored and analyzed as described above.

For studies involving developmental quantification of cardiac specific proteins, we generated tissue lysates from ventricular apical, mid or basal myocardial section at each of the postnatal ages specified.

The husbandry of the mice used in these studies is detailed above. We harvested hearts at each postnatal day between P1 to P17 and snap-froze them in liquid nitrogen. The ventricular apex, mid or base was then separated and after adding 200 µl of RIPA buffer (Cell Signaling

Technology, 9806S) supplemented with phosphatase inhibitor cocktail 2 and 3 (Sigma-Aldrich, P5726-1ML and P0044-1ML), 0.1 mmol/L phenylmethylsulfonyl fluoride (PMSF, Sigma-Aldrich, 93482-50ML-F) and protease inhibitor cocktail (Roche, 11697498001) the samples were homogenized using a Polytron PT 1200E handheld homogenizer (Kinematica). The resulting lysate was centrifuged at 21,000 xg for 30 min, the supernatant fraction harvested and aliquoted into fresh Eppendorf tubes and then snap-frozen in liquid nitrogen. These samples were subsequently fractionated by SDS-PAGE and analyzed as detailed above for cardiomyocyte lysates.

For studies involving different cardiac regions, the heart was divided into atria, RV and LV. In some studies, the LV was further subdivided into apex, mid and base using a sharp scalpel to cut the hearts into pieces of equal width from the apex. On average, the fraction of LV myocardium in LV apex, mid and base was approximately 18%, 38% and 44%, respectively. These regions were then immediately snap frozen in liquid nitrogen and subsequently used to prepare lysates, as above.

Antibodies used for immunoblots are detailed in Supplementary Table S1. Most of these antibodies are profiled in 1DegreeBio and were additionally validated in siRNA knockdown studies.

Supplementary Table S1. Information about the Antibodies Used in this Study

Antibody	Vendor	Catalogue number
c-Jun	Cell Signaling Technology	9165
cTnT	Miltenyi Biotec	130-119-674
Cyclin A2	Abcam	ab181591
Cyclin B1	Abcam	ab32053
Cyclin D1	Abcam	ab134175
DUSP5	Abcam	ab200708
ERK1/2	Cell Signaling Technology	4695
GAPDH	Cell Signaling Technology	2118
IGF-1	Abcam	Ab9572
IGF-1R	Cell Signaling Technology	9750
MEK1/2	Cell Signaling Technology	9126
Phospho-ERK1/2 (T202/Y204)	Cell Signaling Technology	4370
Phospho-histone H3 (S10)	Cell Signaling Technology	8481
Phospho-MEK (S217/221)	Cell Signaling Technology	9121

Most of these antibodies are profiled in 1DegreeBio and were also validated using siRNA.

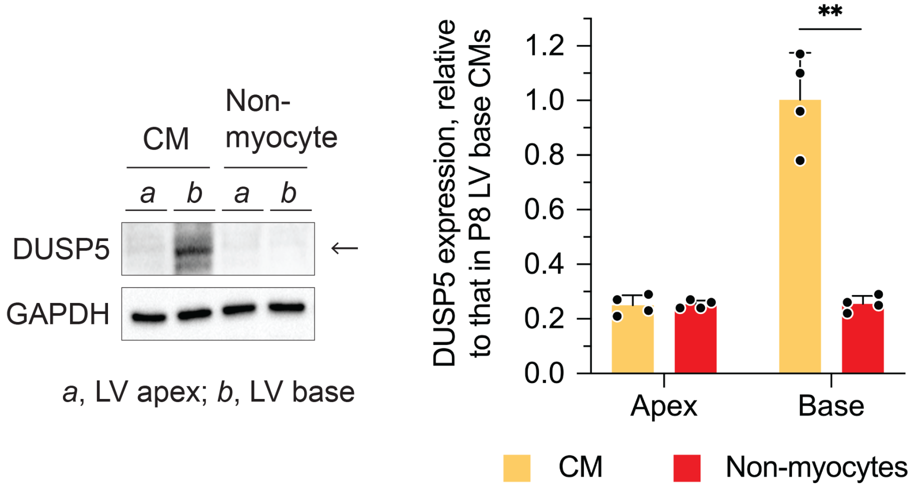

Supplementary Figure 1. DUSP5 levels in cardiomyocytes (CMs) and non-myocytes of the P8 LV apex and base. Representative immunoblots show expression levels of indicated proteins in whole lysates obtained from CMs and non-myocytes of the P8 LV apex and base. Quantitative data is shown next to the immunoblots. Data are mean ± SEM. Comparisons between protein levels in CMs and non-myocytes were made using a 2-sided Student's *t*-test. $^{**}P < 0.01$.

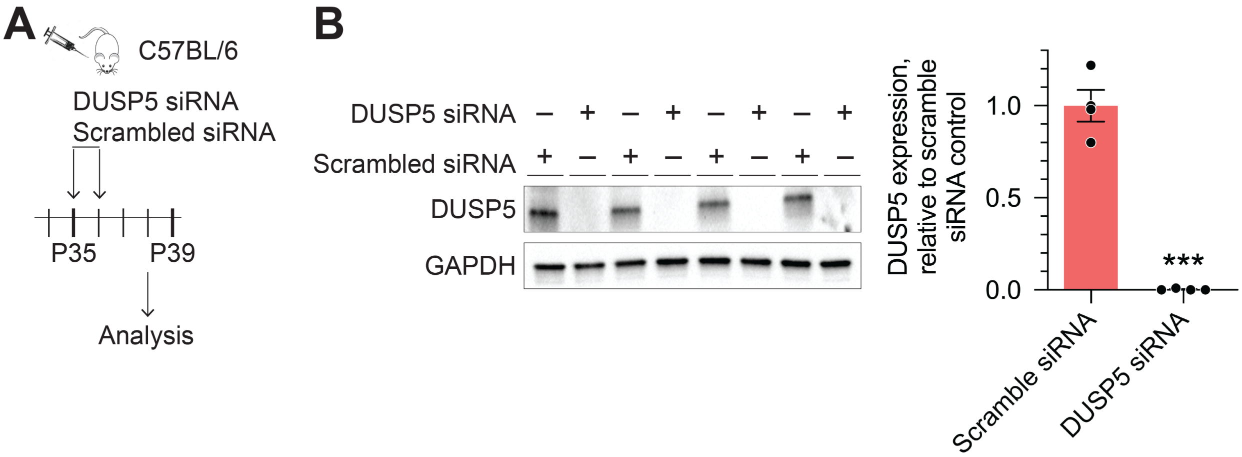

Supplementary Figure 2. DUSP5 levels in ventricular cardiomyocytes (CMs) in mice treated with DUSP5 siRNA or scrambled siRNA control. (**A**) Protocol for *in vivo* siRNA treatment. (**B**) Immunoblot shows expression levels of indicated proteins in whole lysates obtained from ventricular CMs of 5-week-old mice. Quantitative data is shown next to the immunoblots. Data are mean ± SEM. Comparisons between protein levels in CMs and non-myocytes were made using a 2-sided Student's *t*-test. ***$P < 0.001$.

Full unedited gel for Figure 1B

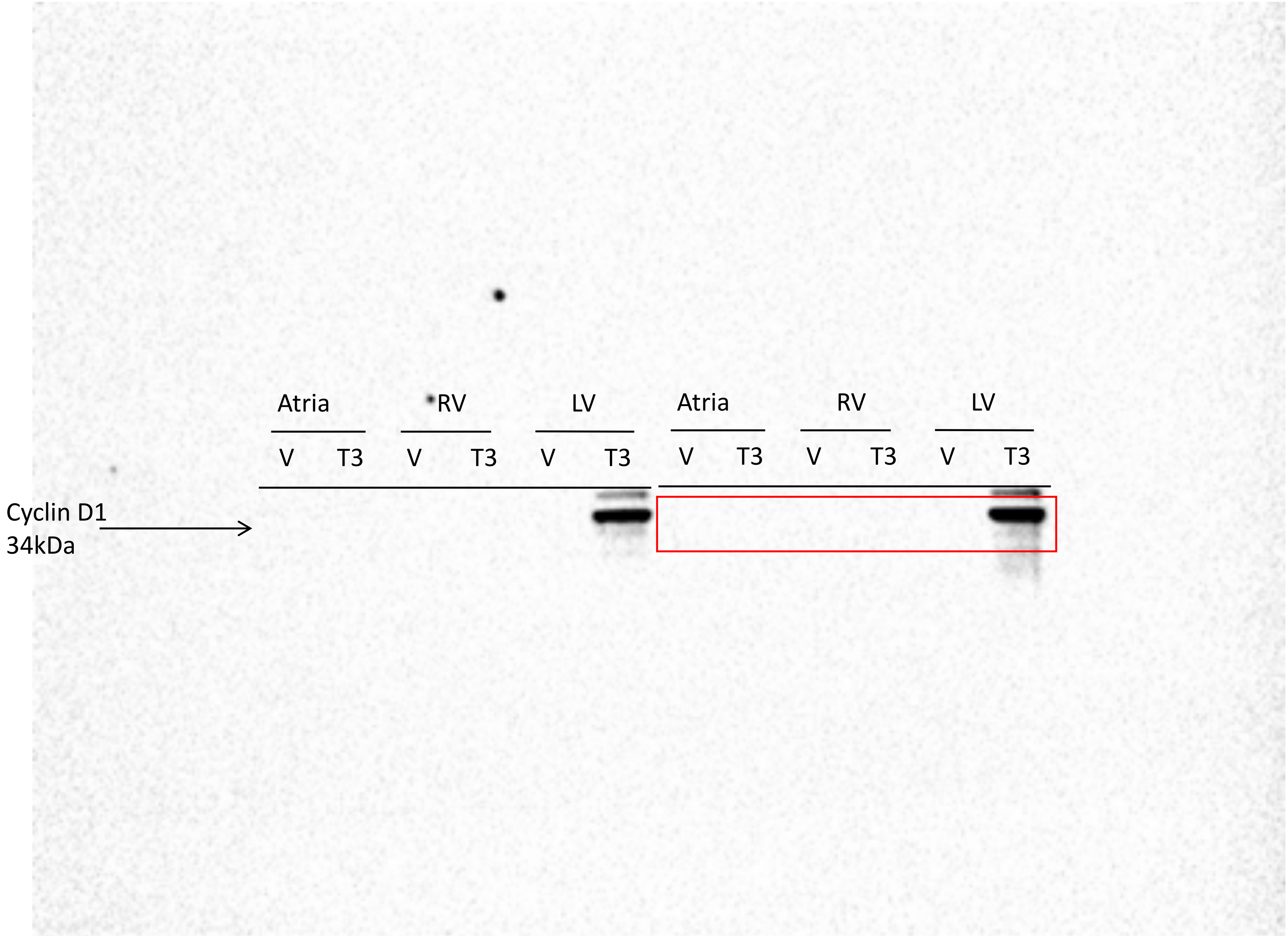

Full unedited gel for Figure 1B

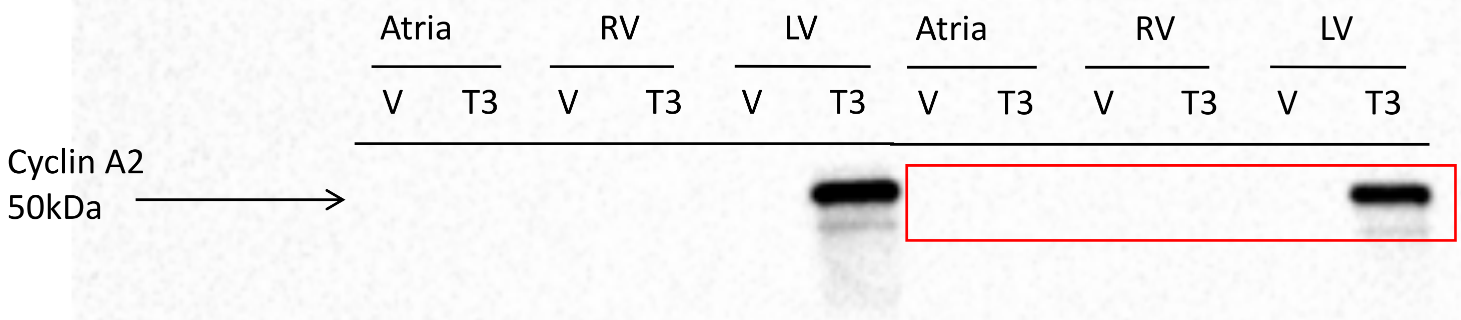

Full unedited gel for Figure 1B

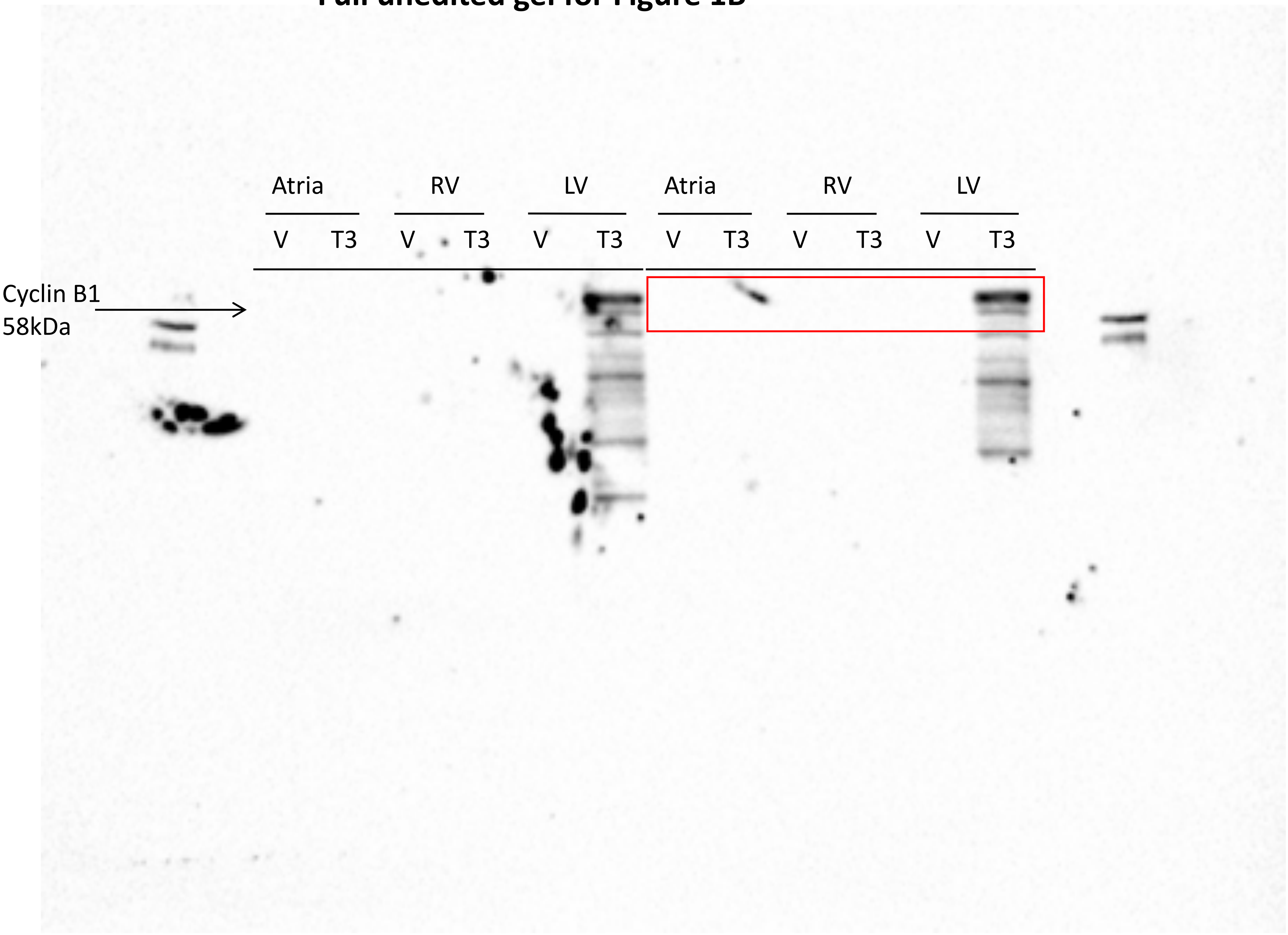

Full unedited gel for Figure 1B (over exposed)

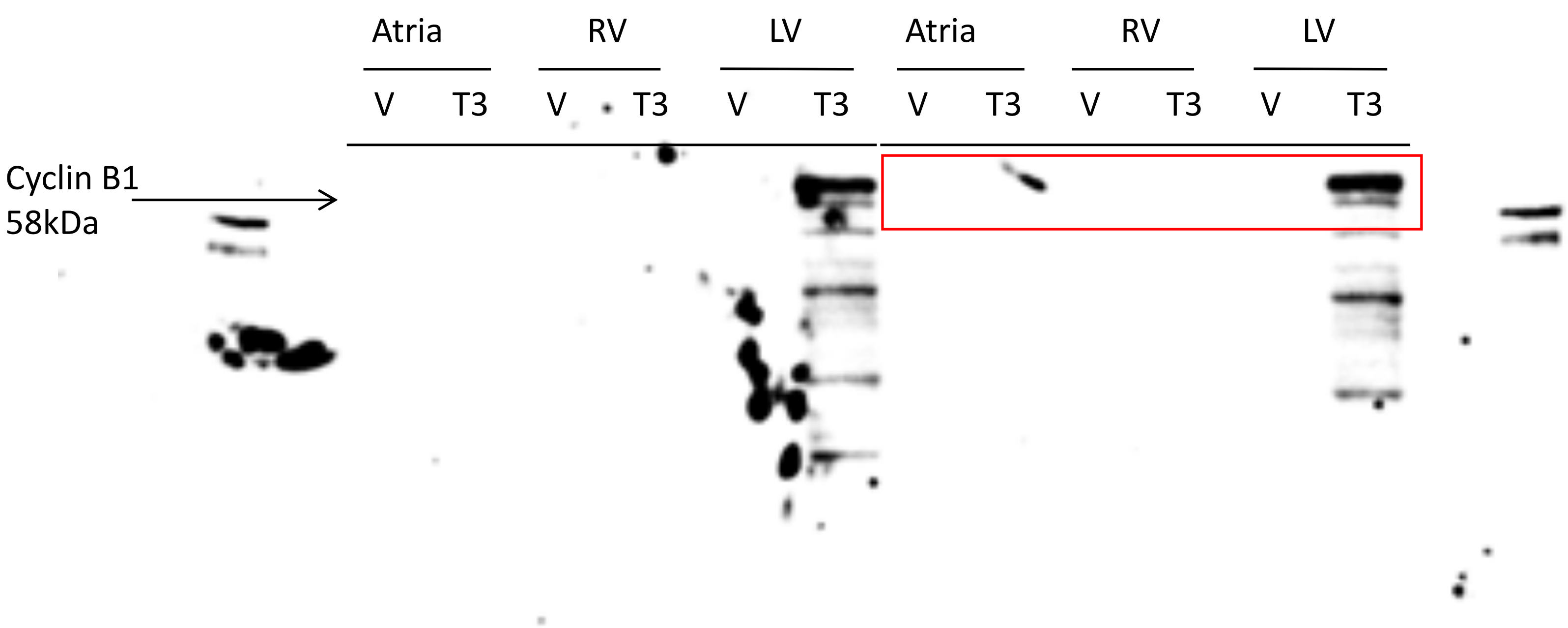

Full unedited gel for Figure 1B (under exposed)

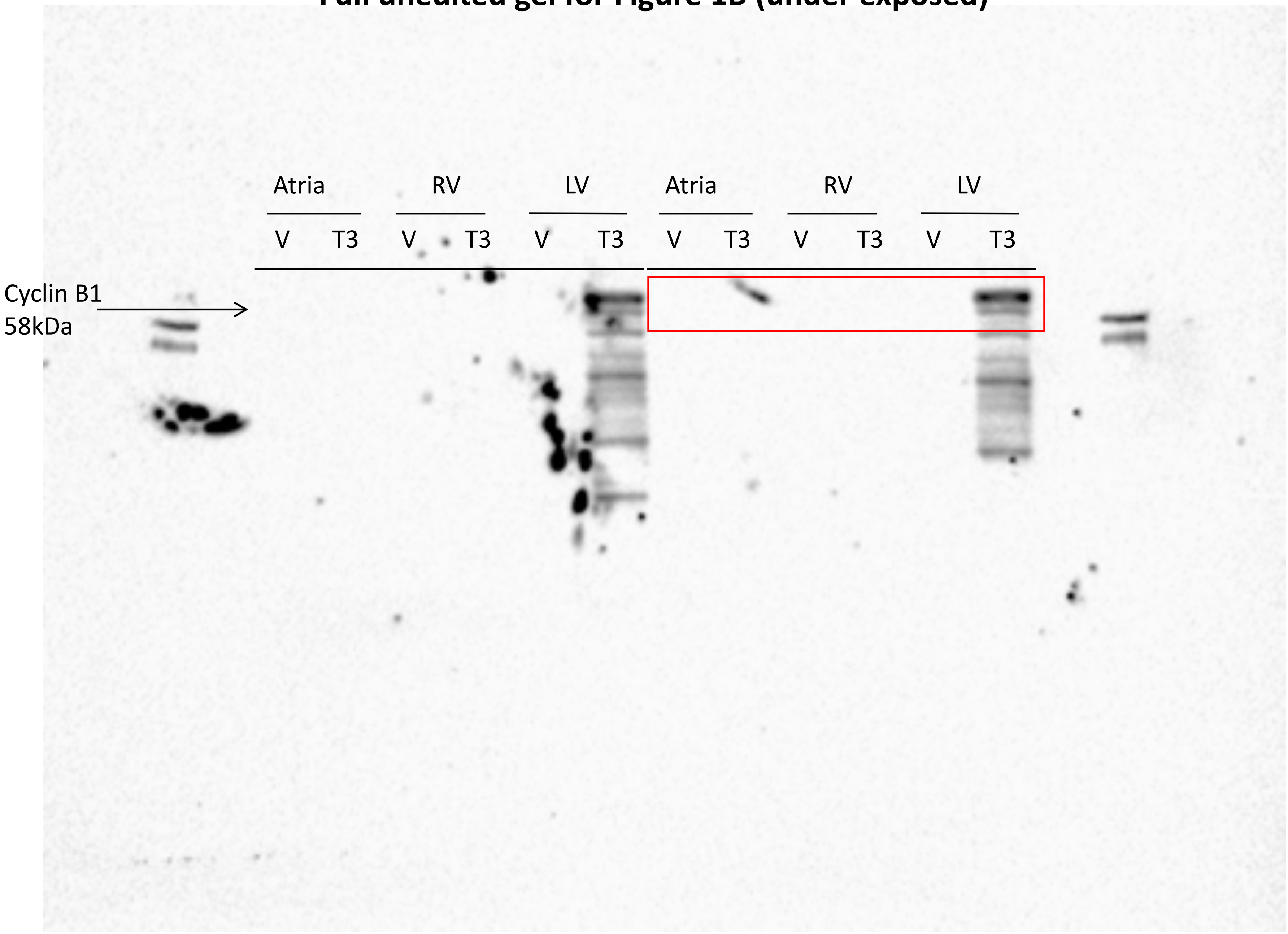

Full unedited gel for Figure 1B

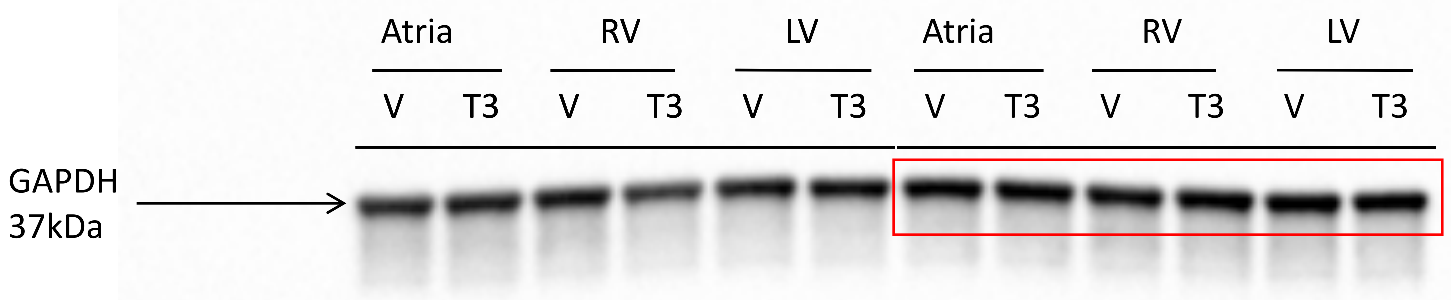

Full unedited gel for Figure 1C

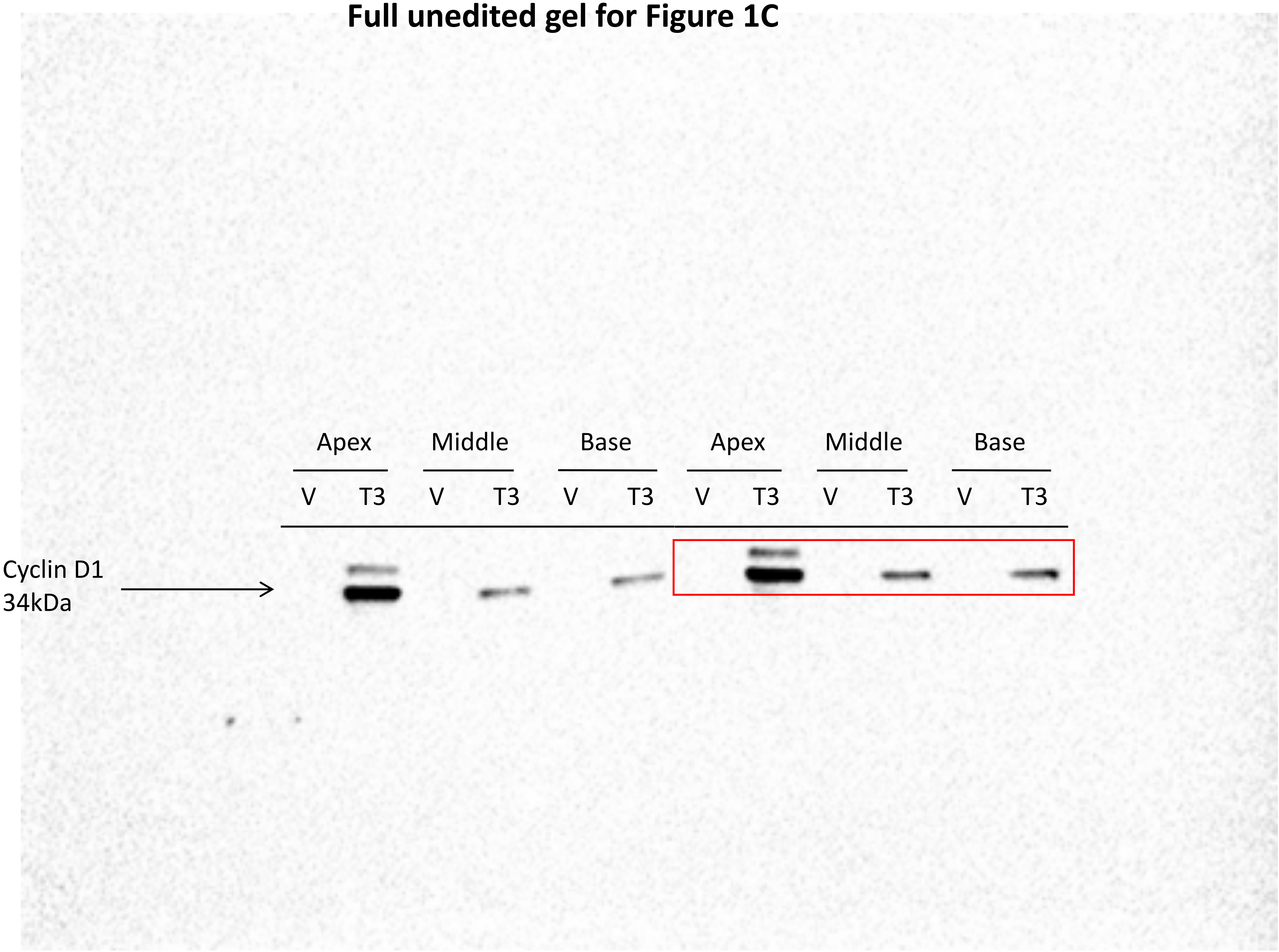

Full unedited gel for Figure 1C

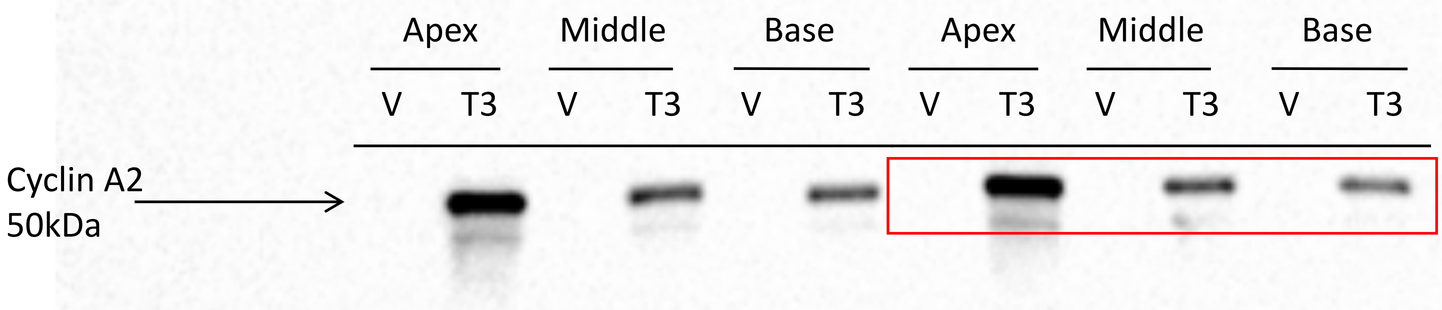

Full unedited gel for Figure 1C

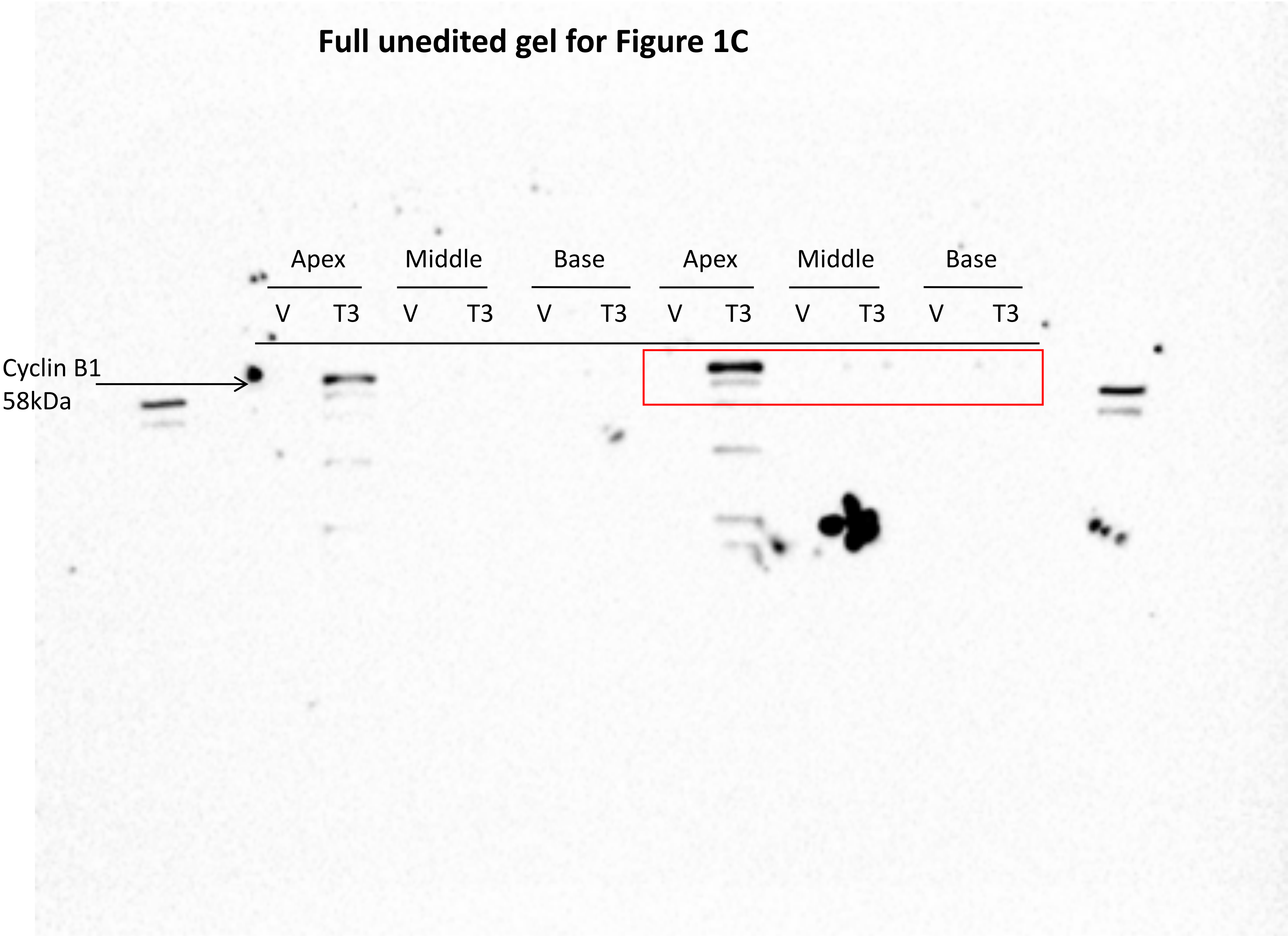

Full unedited gel for Figure 1C

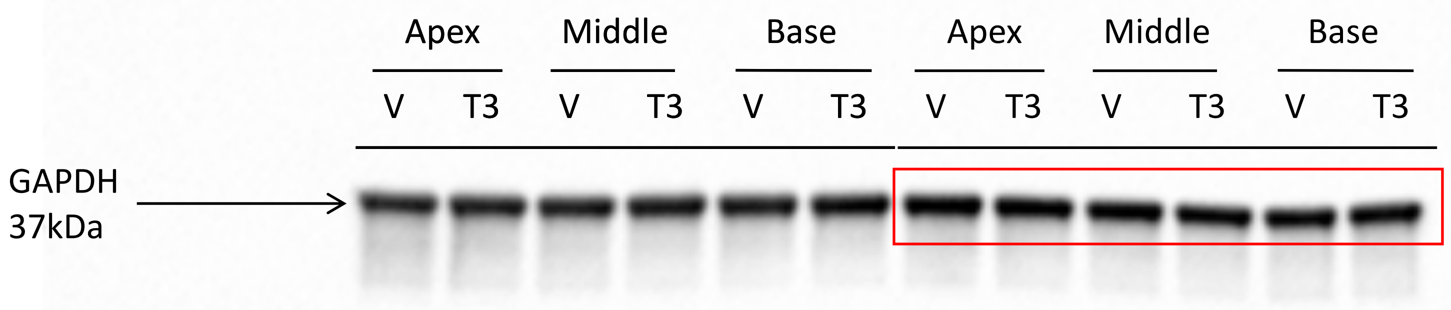

Full unedited gel for Figure 3B-LV apex

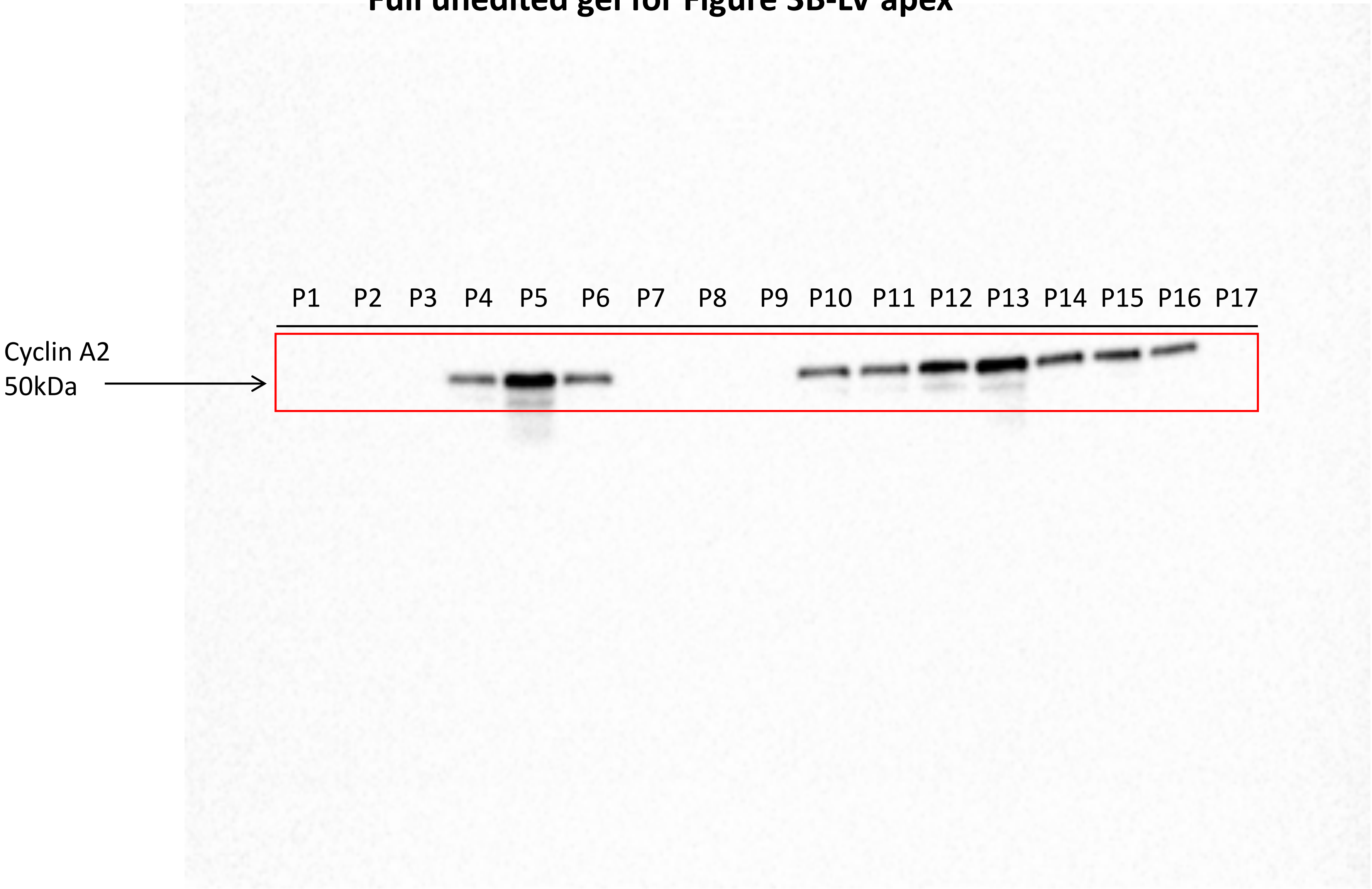

Full unedited gel for Figure 3B-LV apex

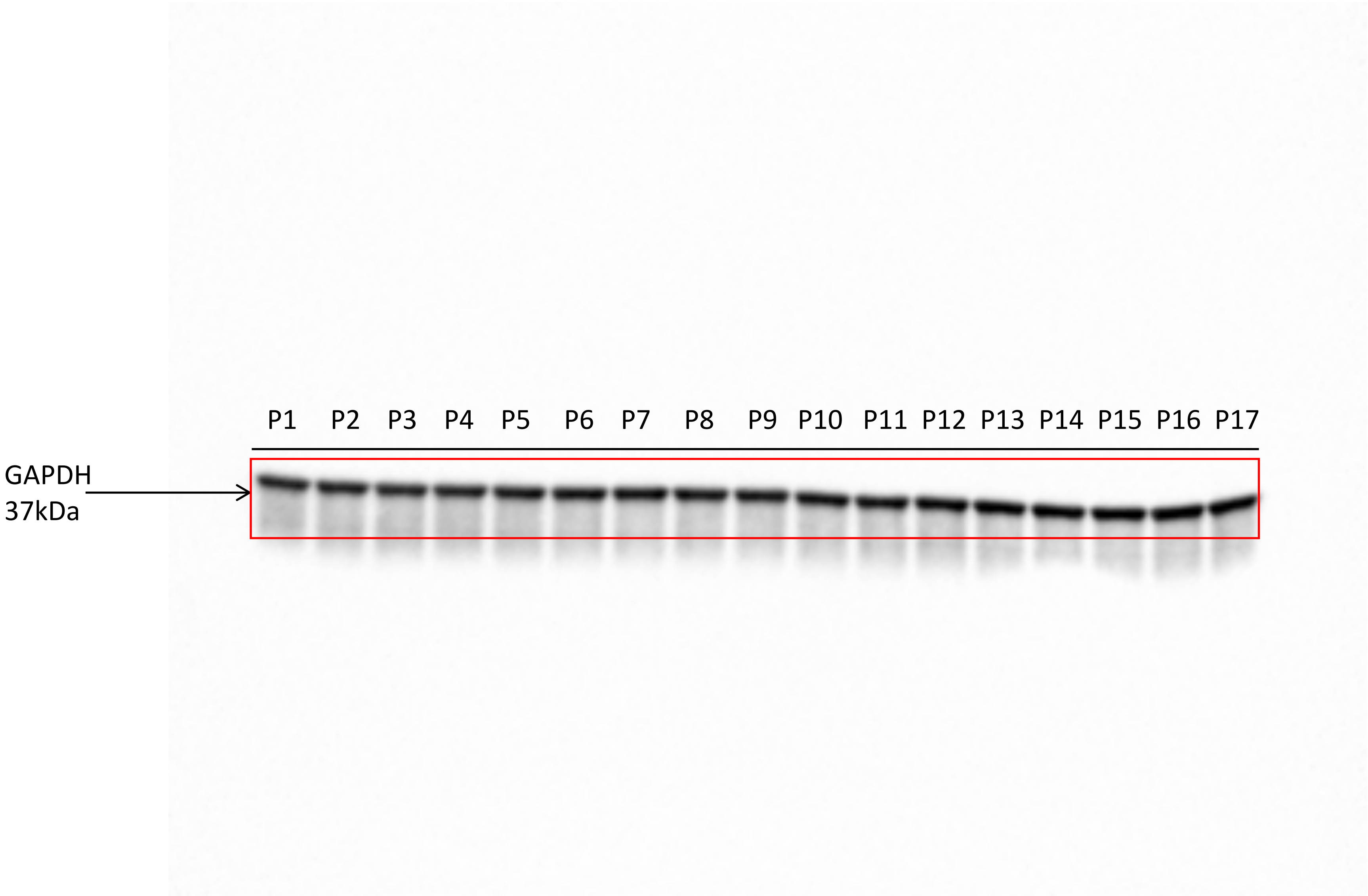

Full unedited gel for Figure 3B-LV base

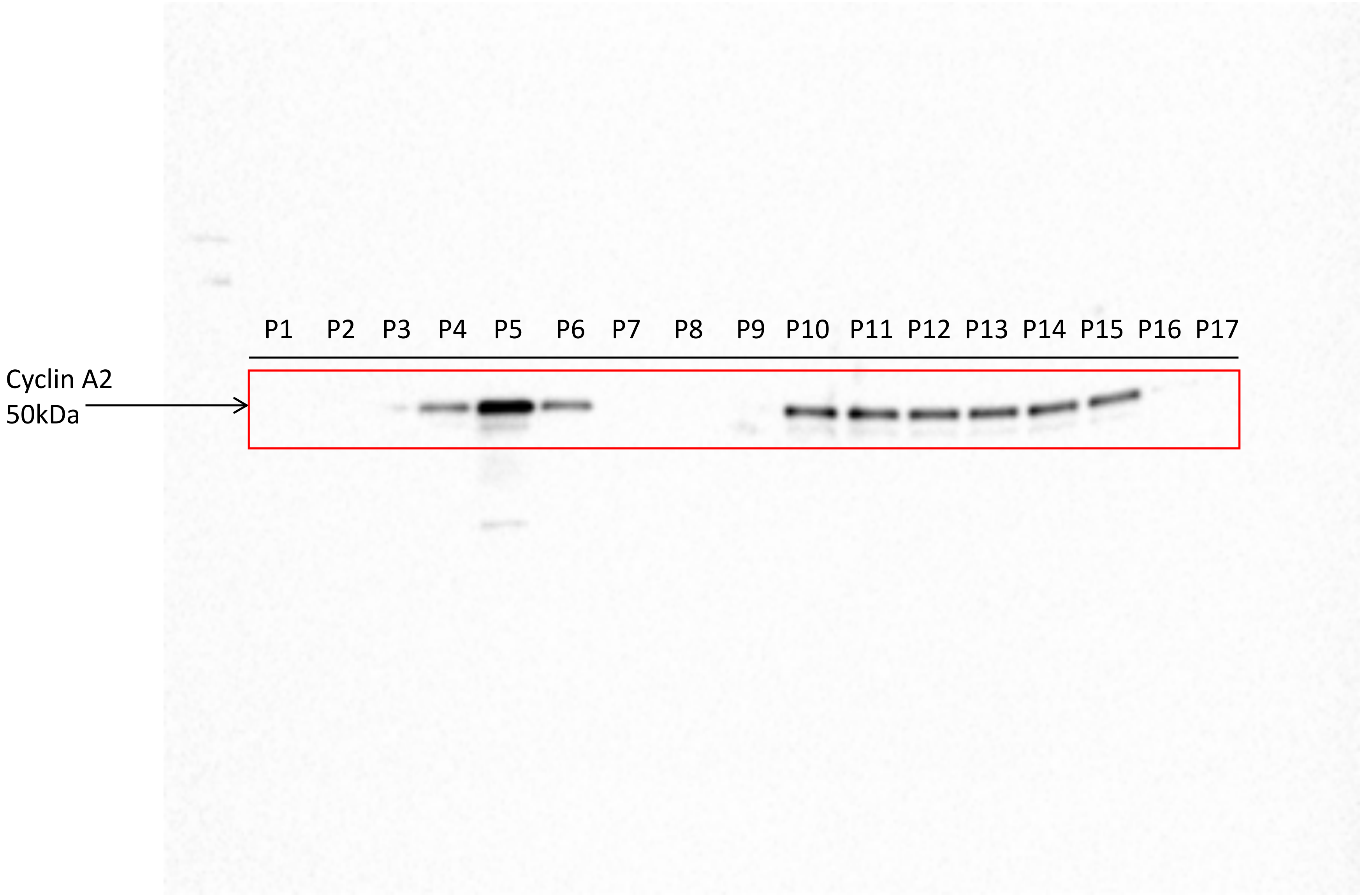

Full unedited gel for Figure 3B-LV base

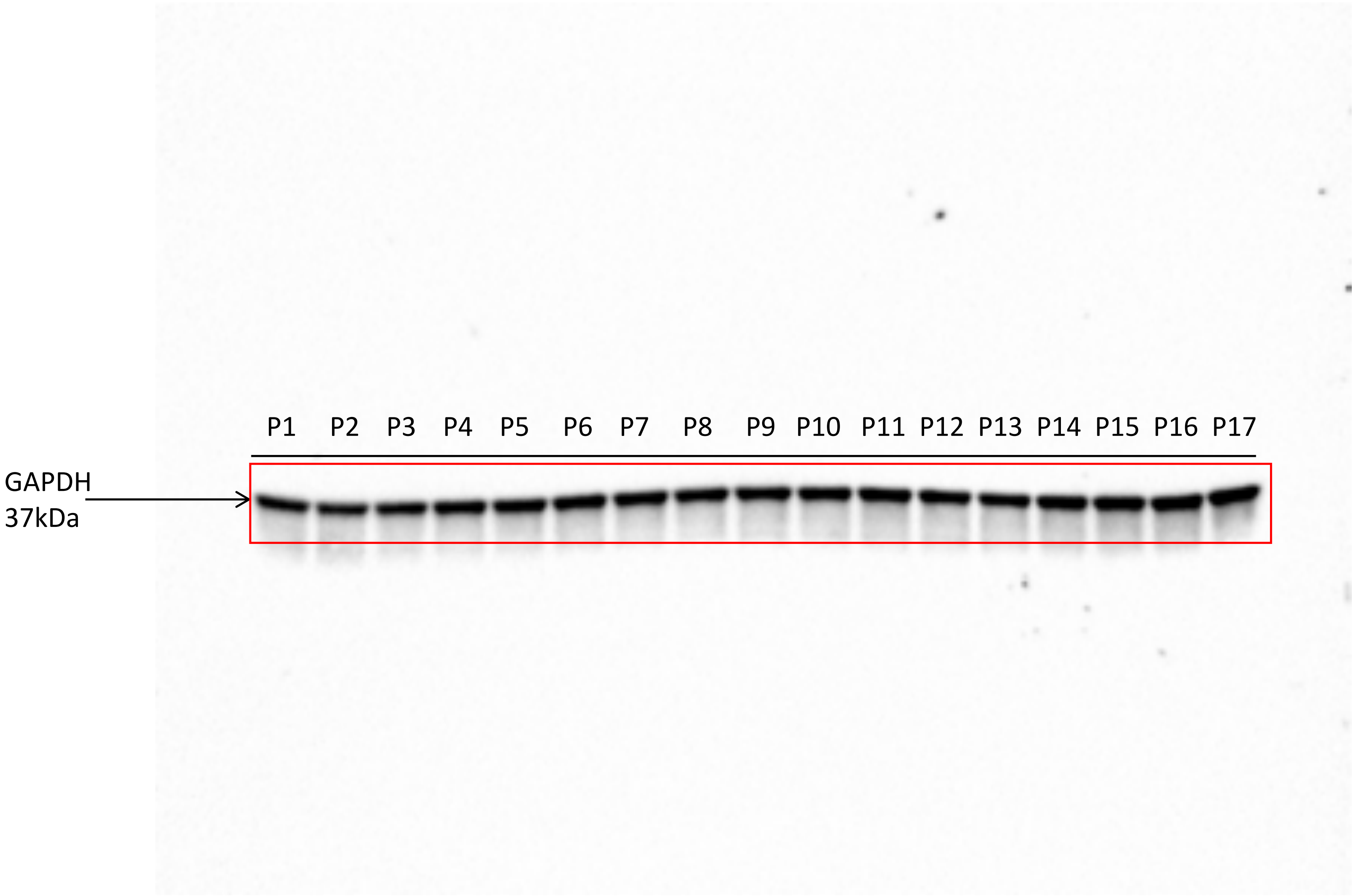

Full unedited gel for Figure 3D

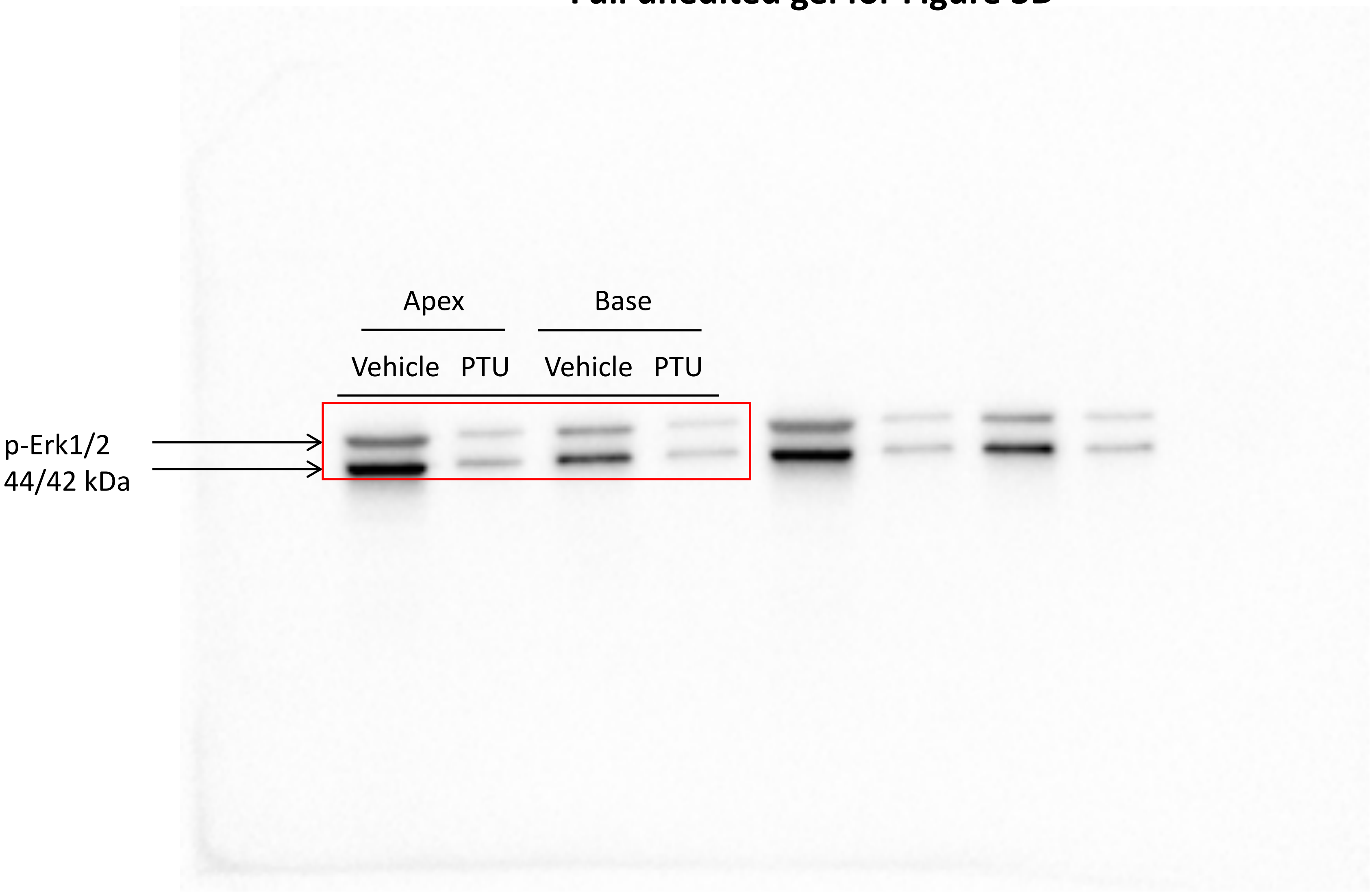

Full unedited gel for Figure 3D

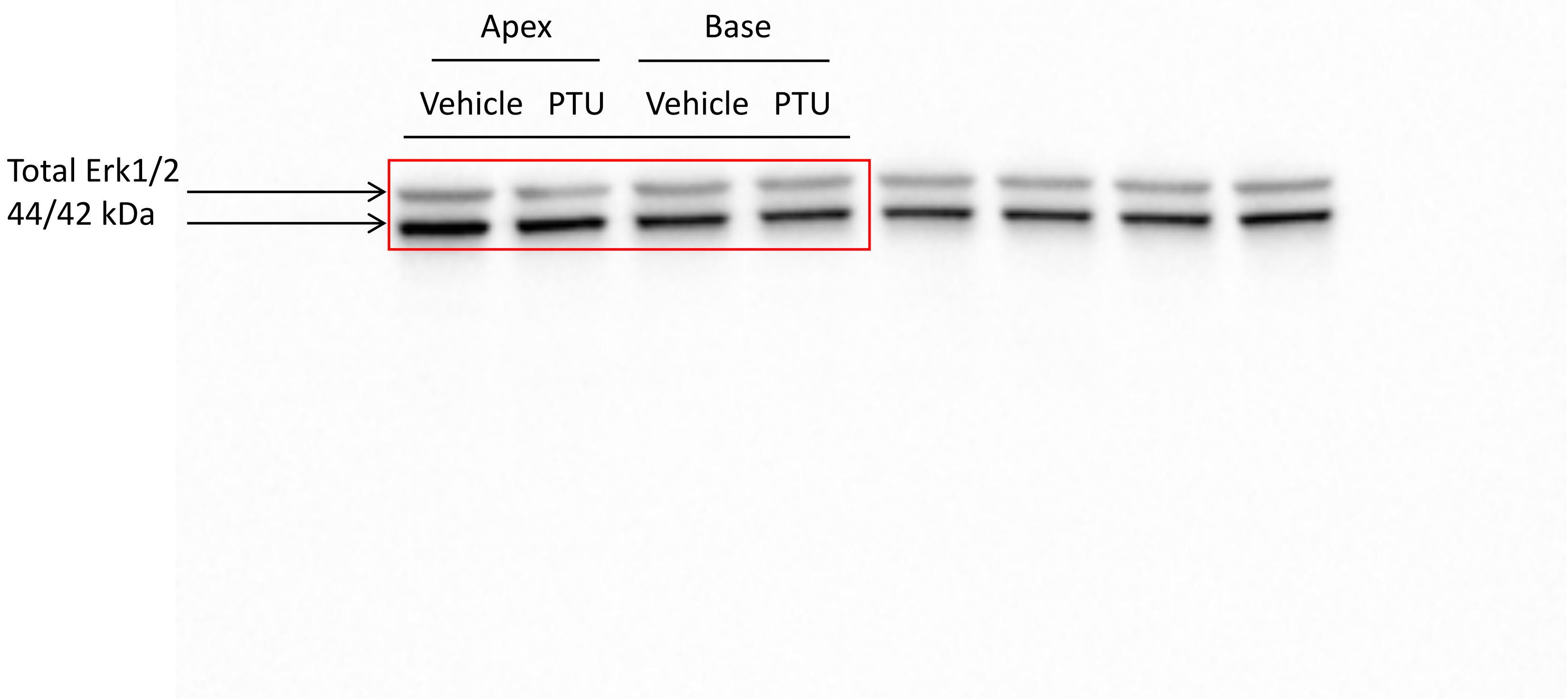

Full unedited gel for Figure 3D

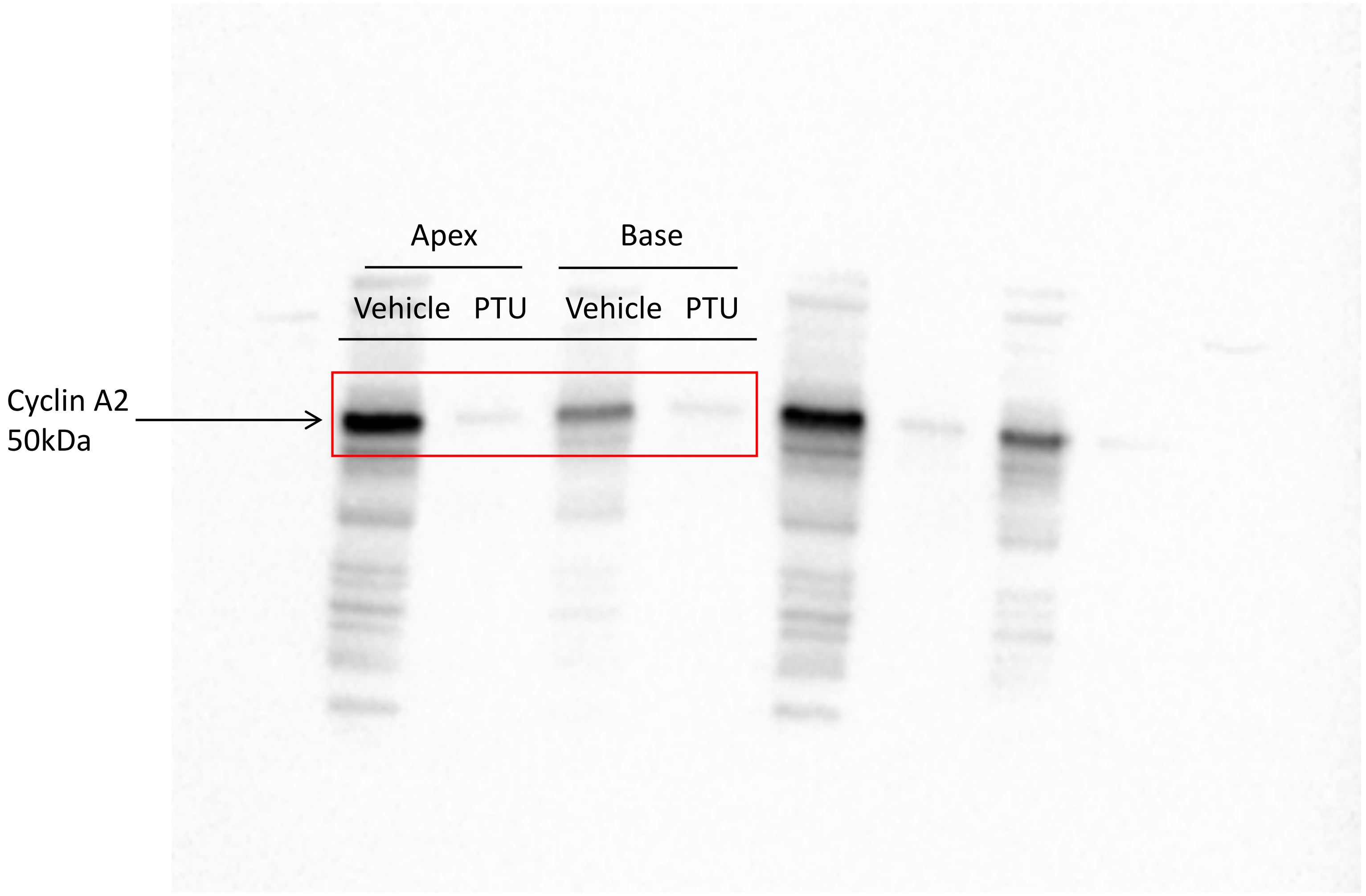

Full unedited gel for Figure 3D

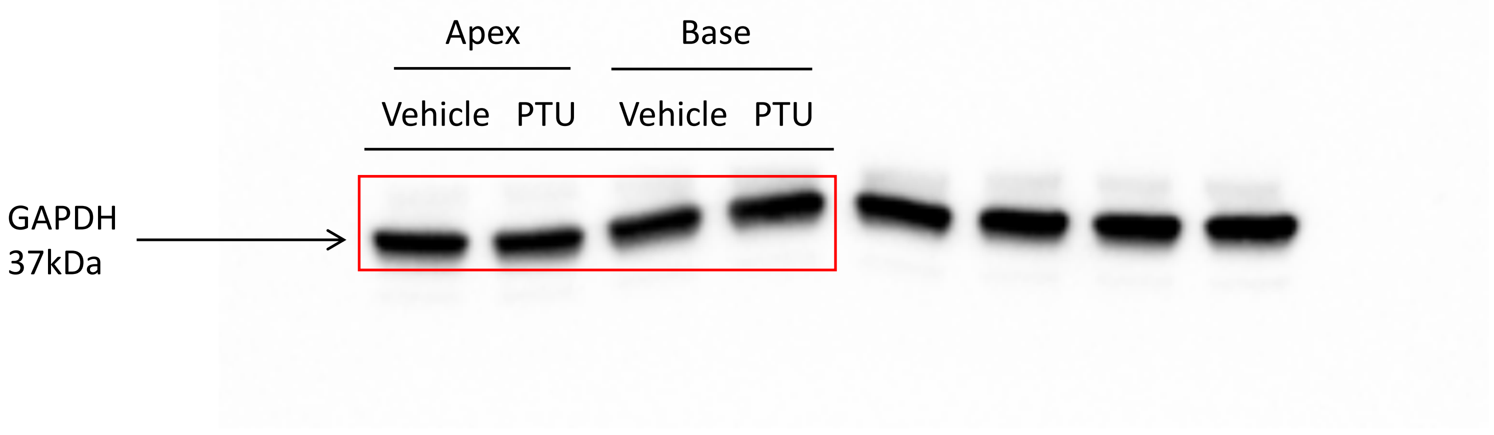

Full unedited gel for figure 4B

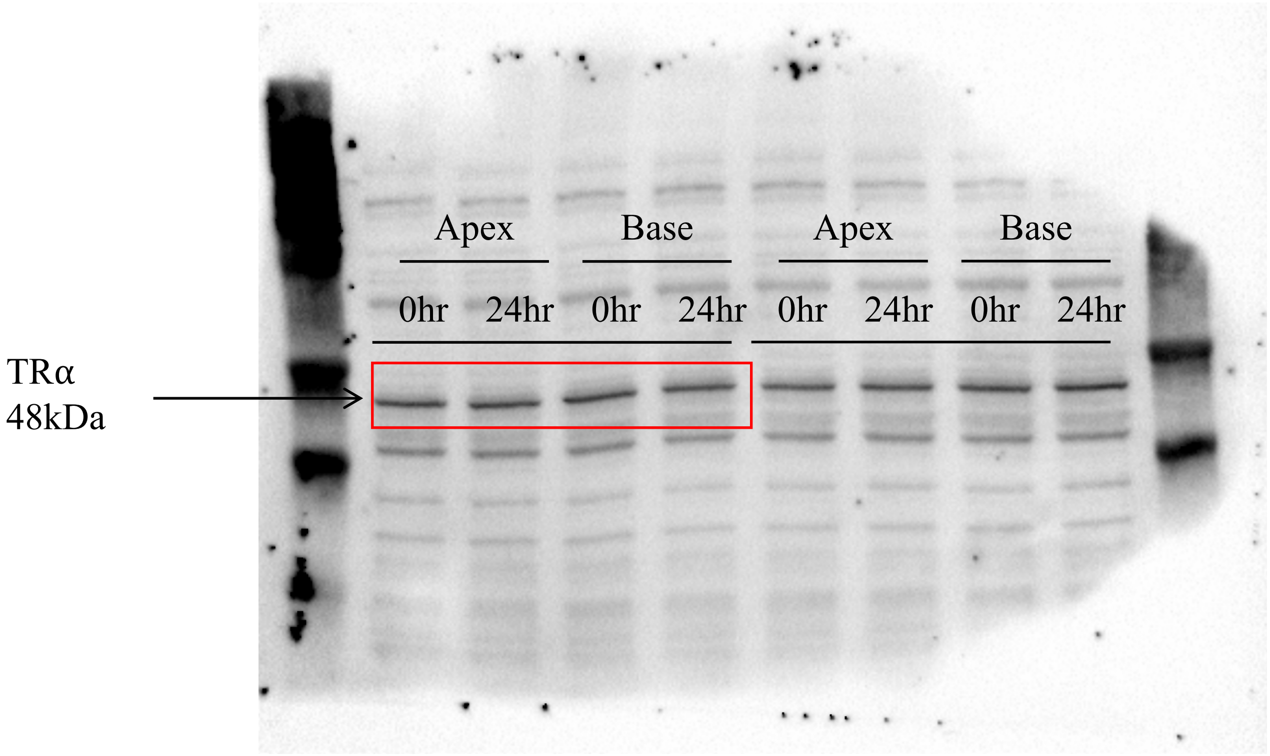

Full unedited gel for figure 4B (Less exposure)

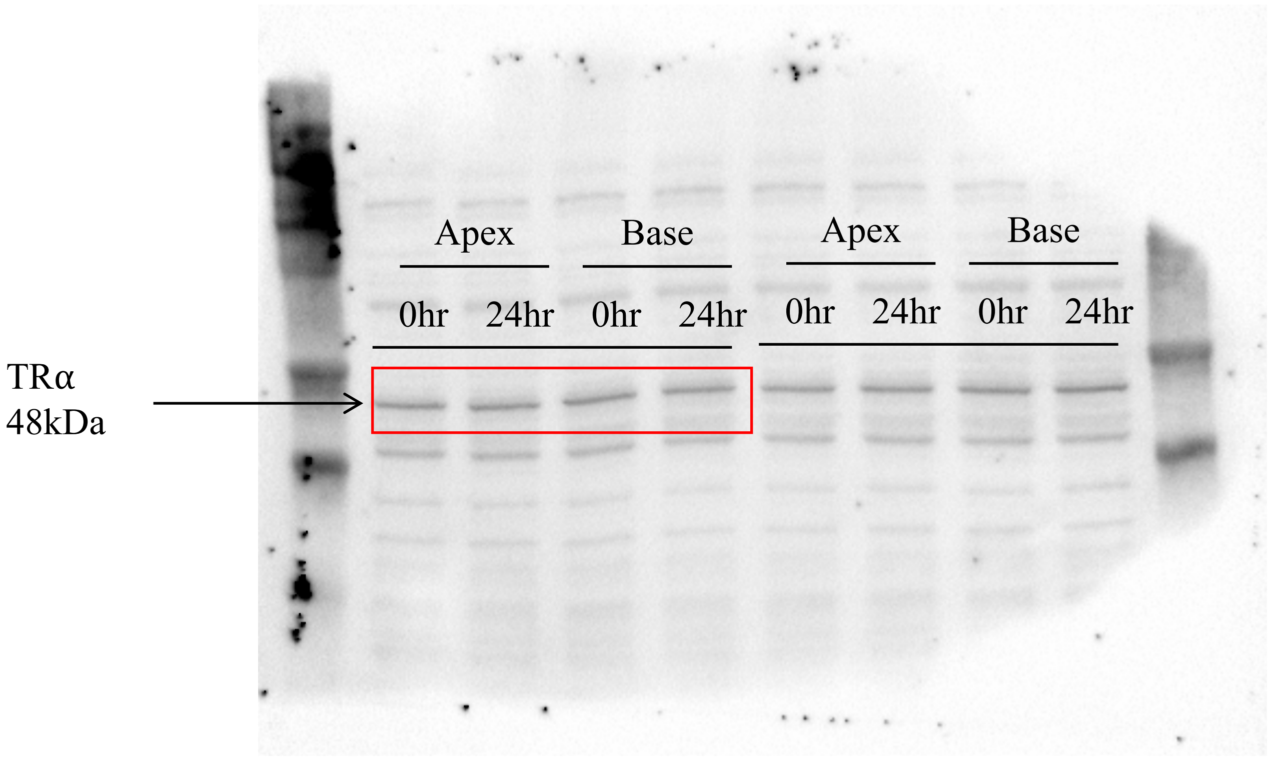

Full unedited gel for figure 4B (overexposure)

TRα
48kDa

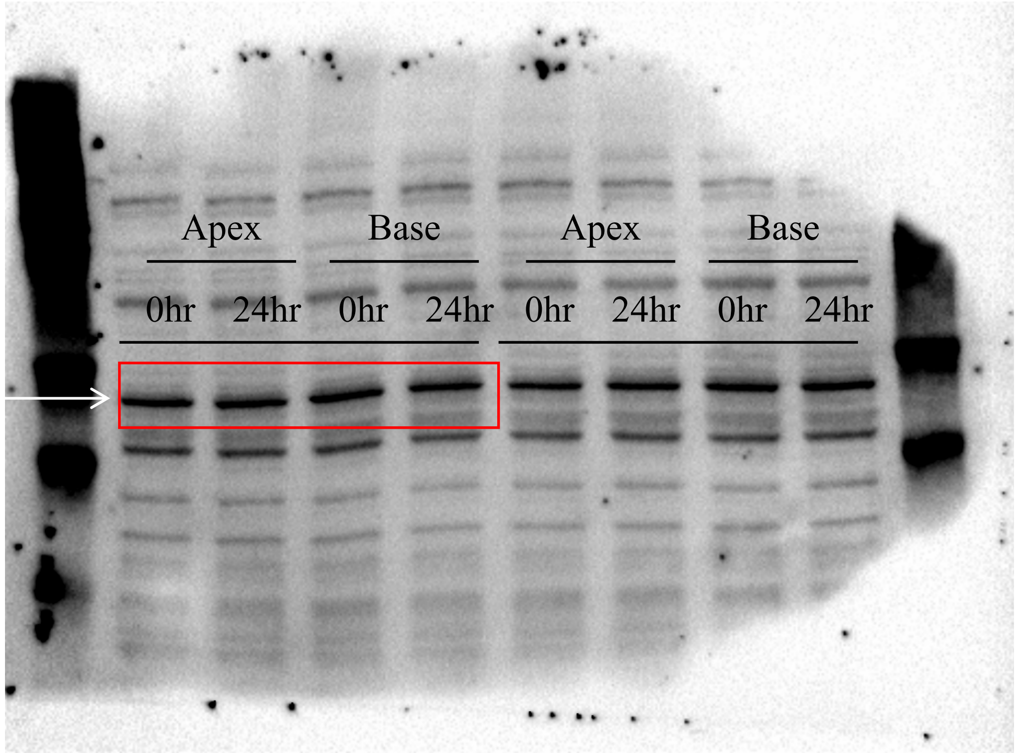

Full unedited gel for Figure 4B

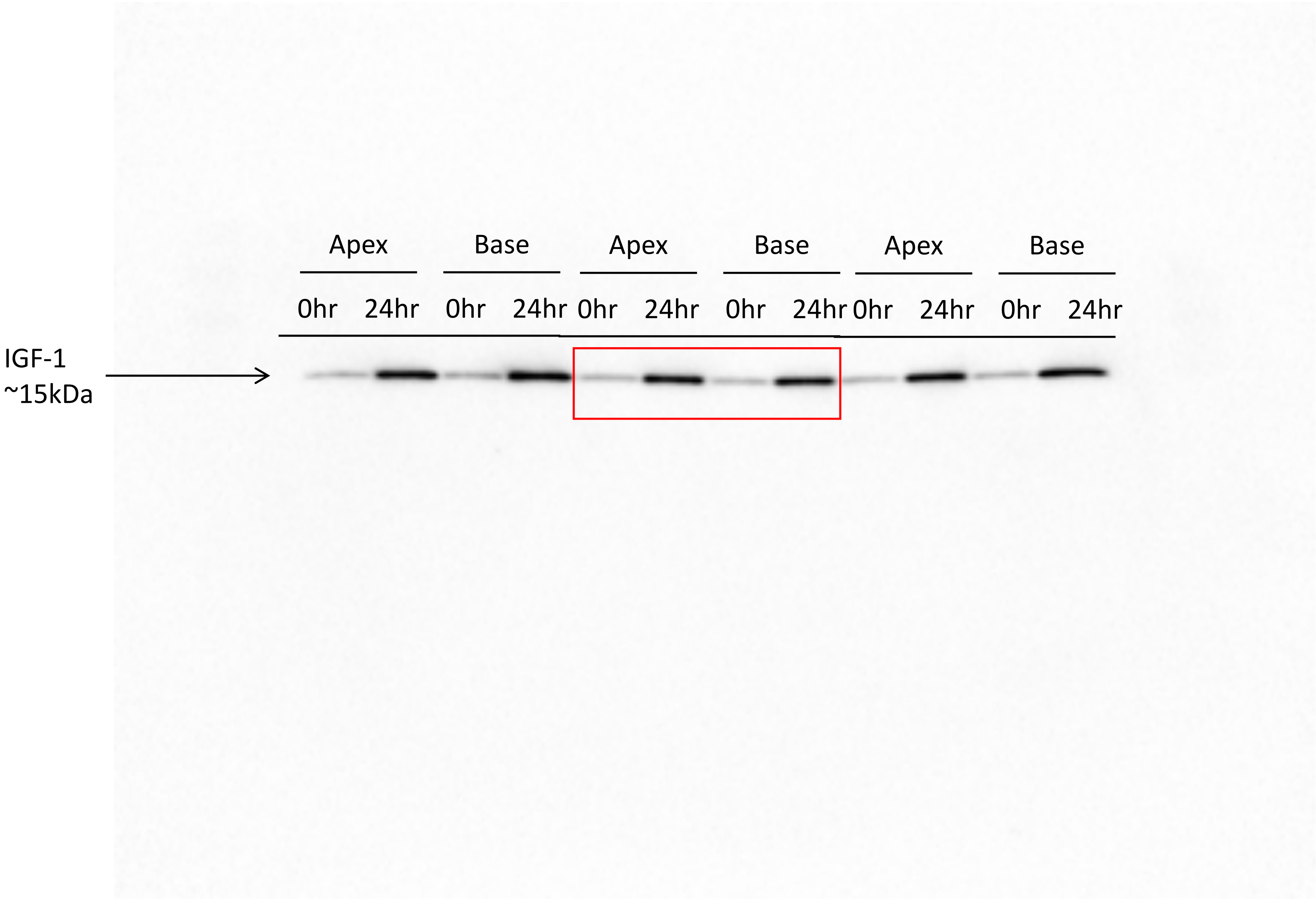

Full unedited gel for Figure 4B

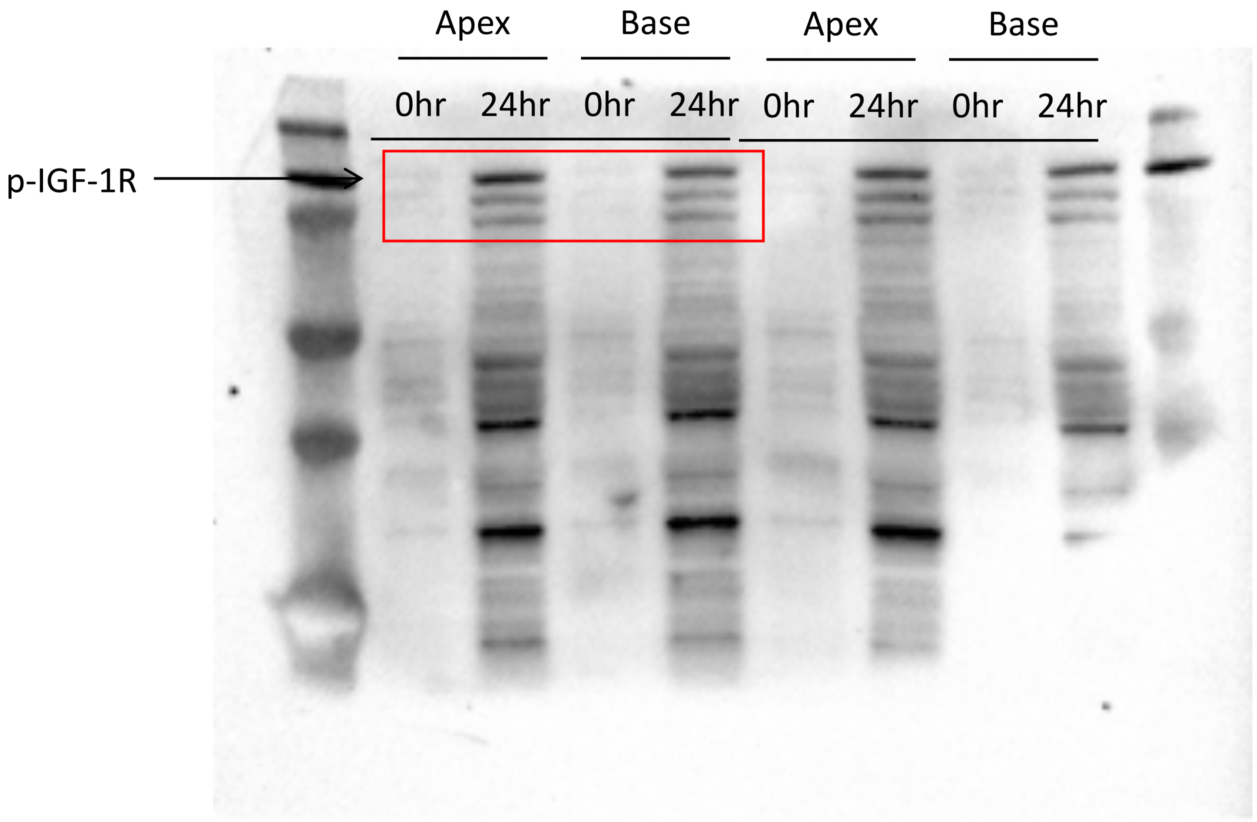

Full unedited gel for Figure 4B

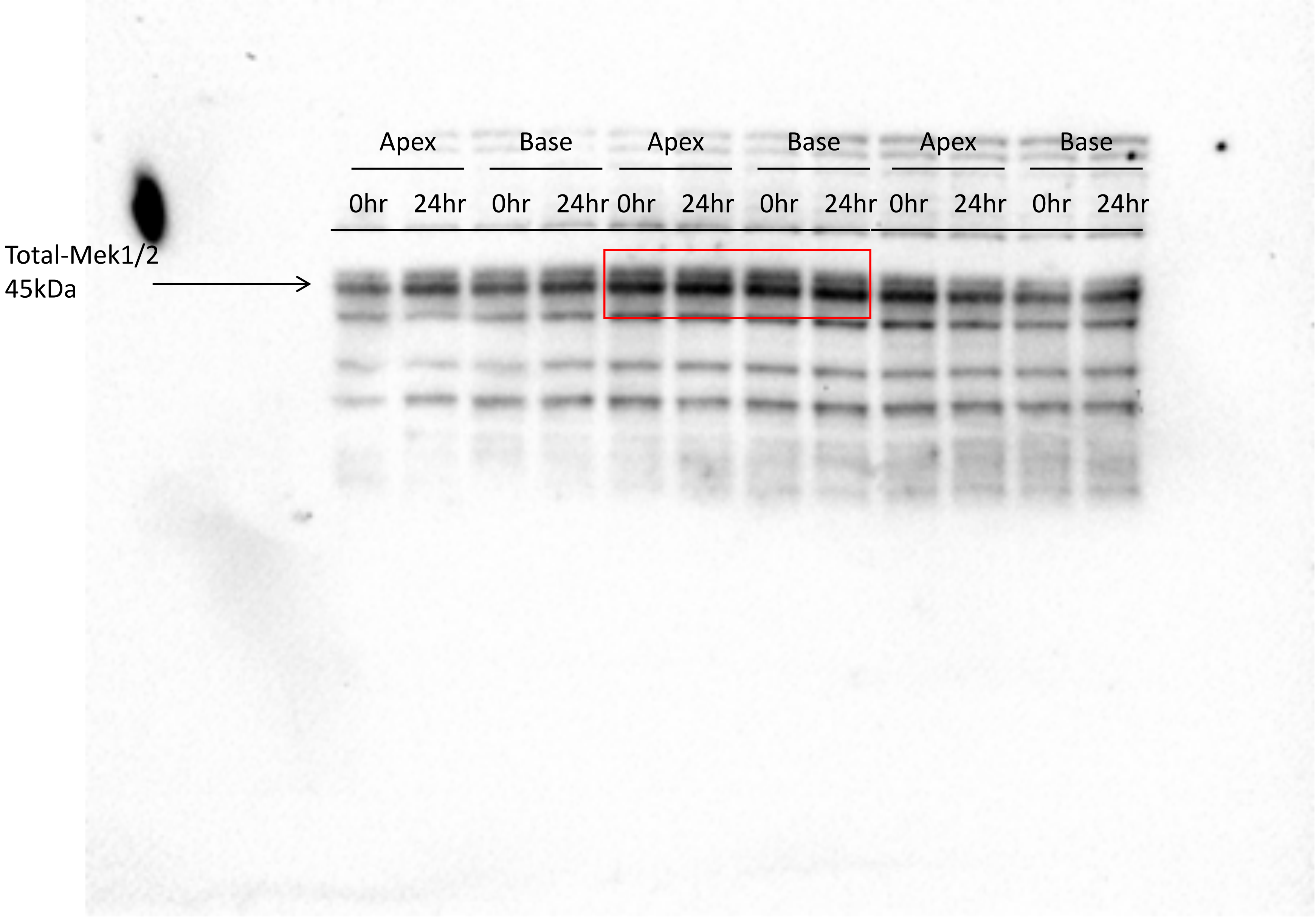

Full unedited gel for Figure 4B

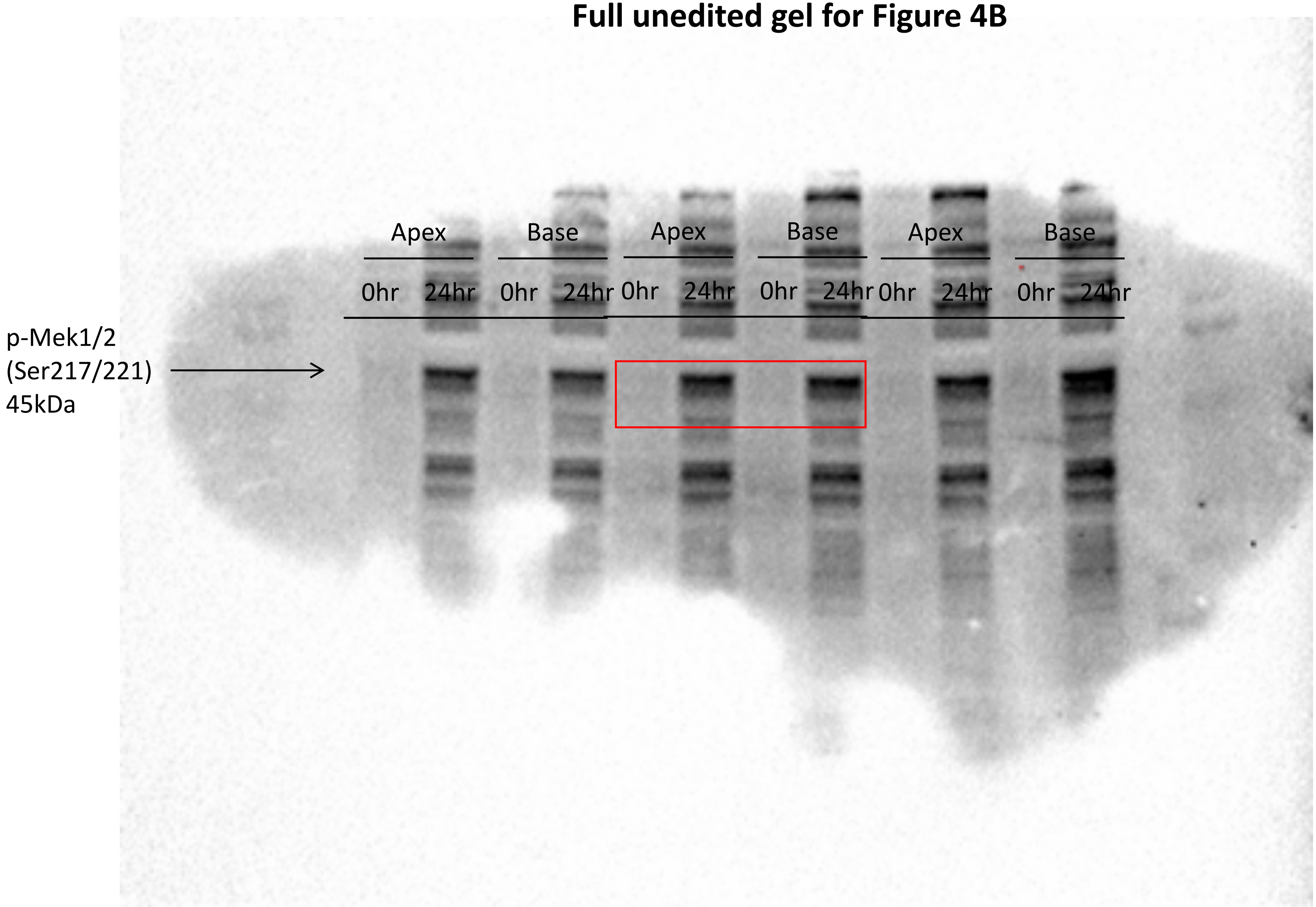

Full unedited gel for Figure 4B (over exposed)

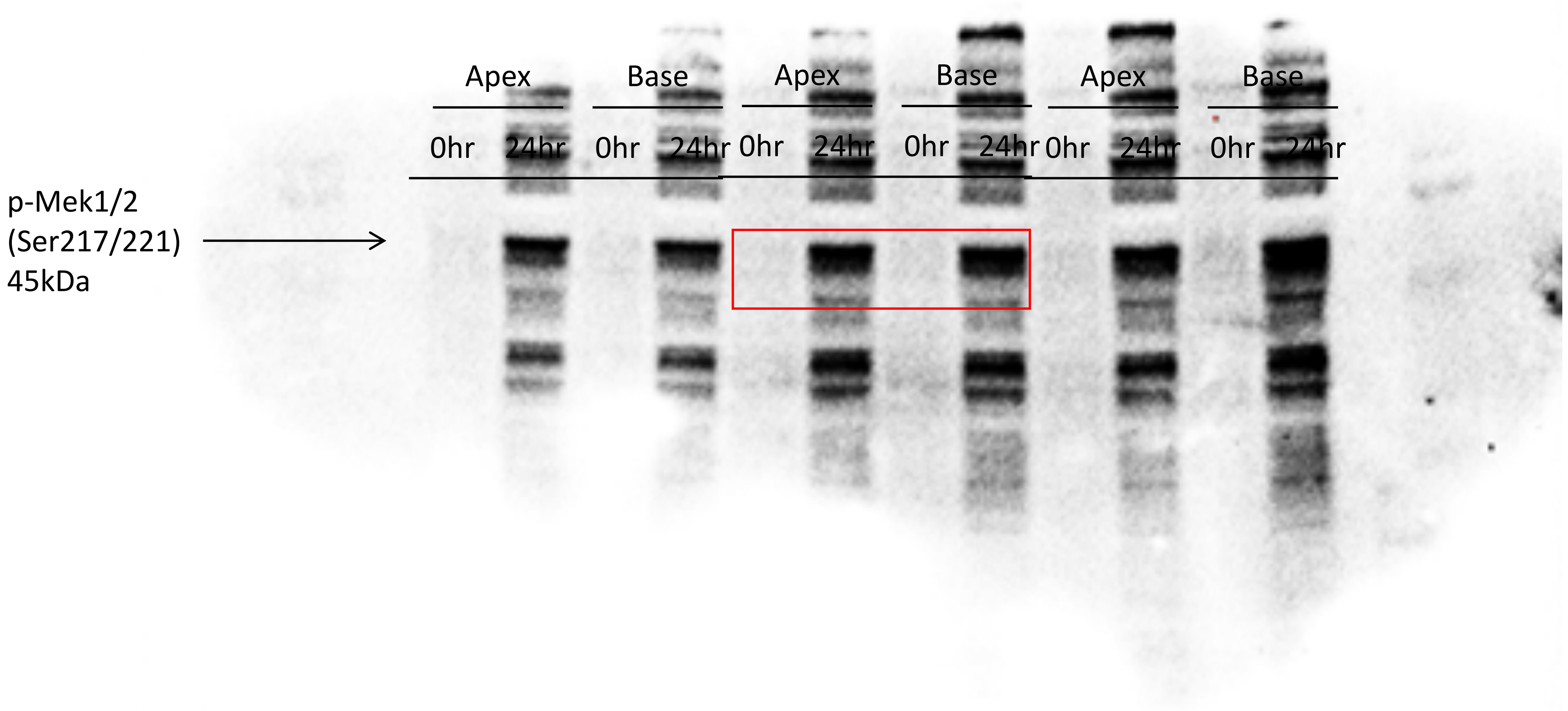

Full unedited gel for Figure 4B (under exposed)

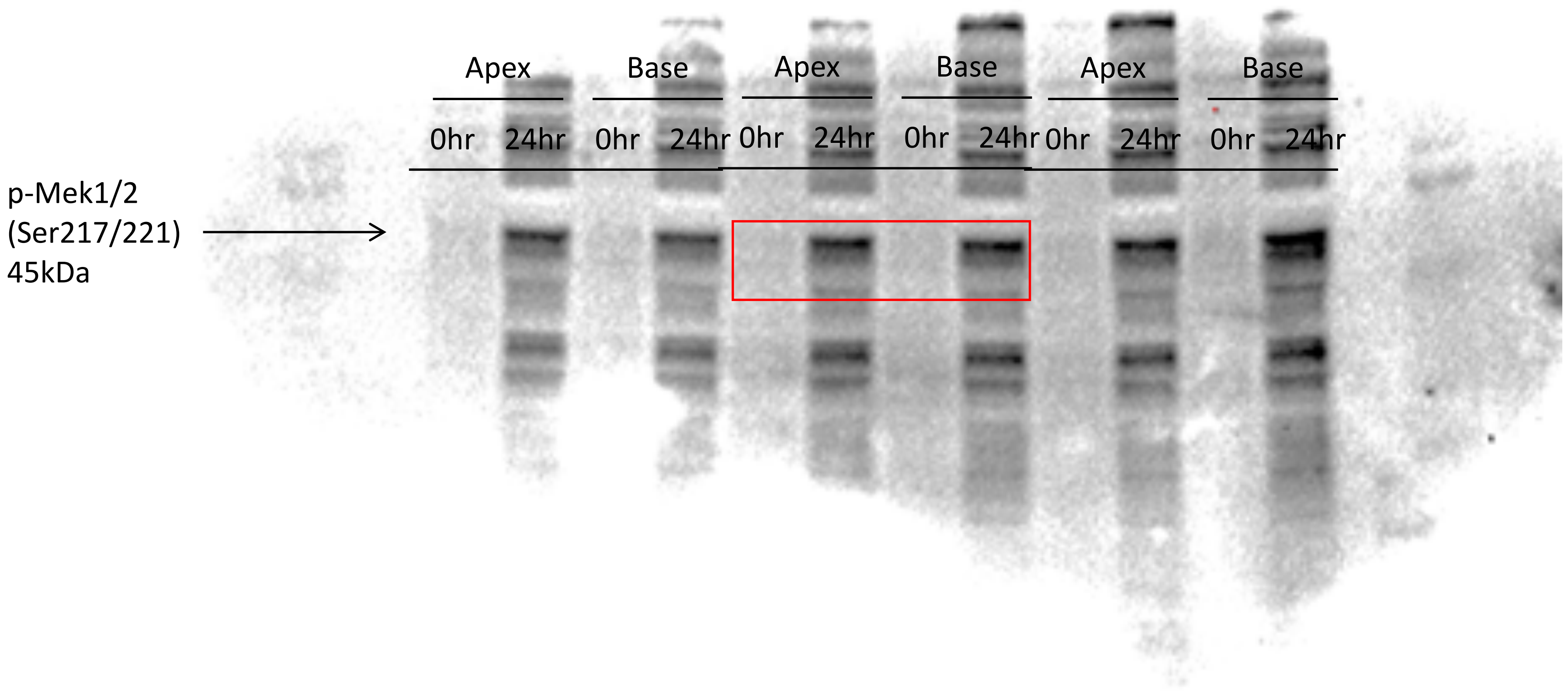

Full unedited gel for Figure 4B

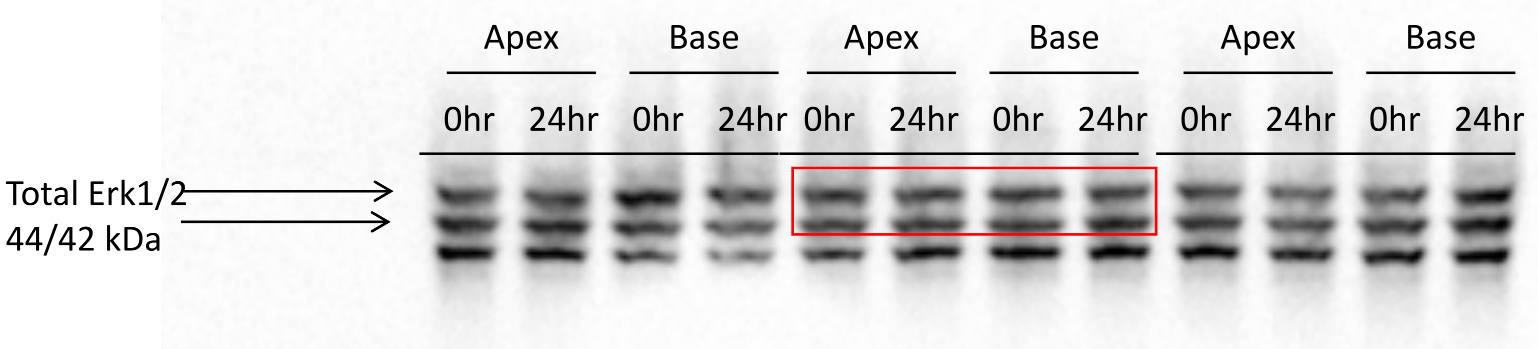

Full unedited gel for Figure 4B

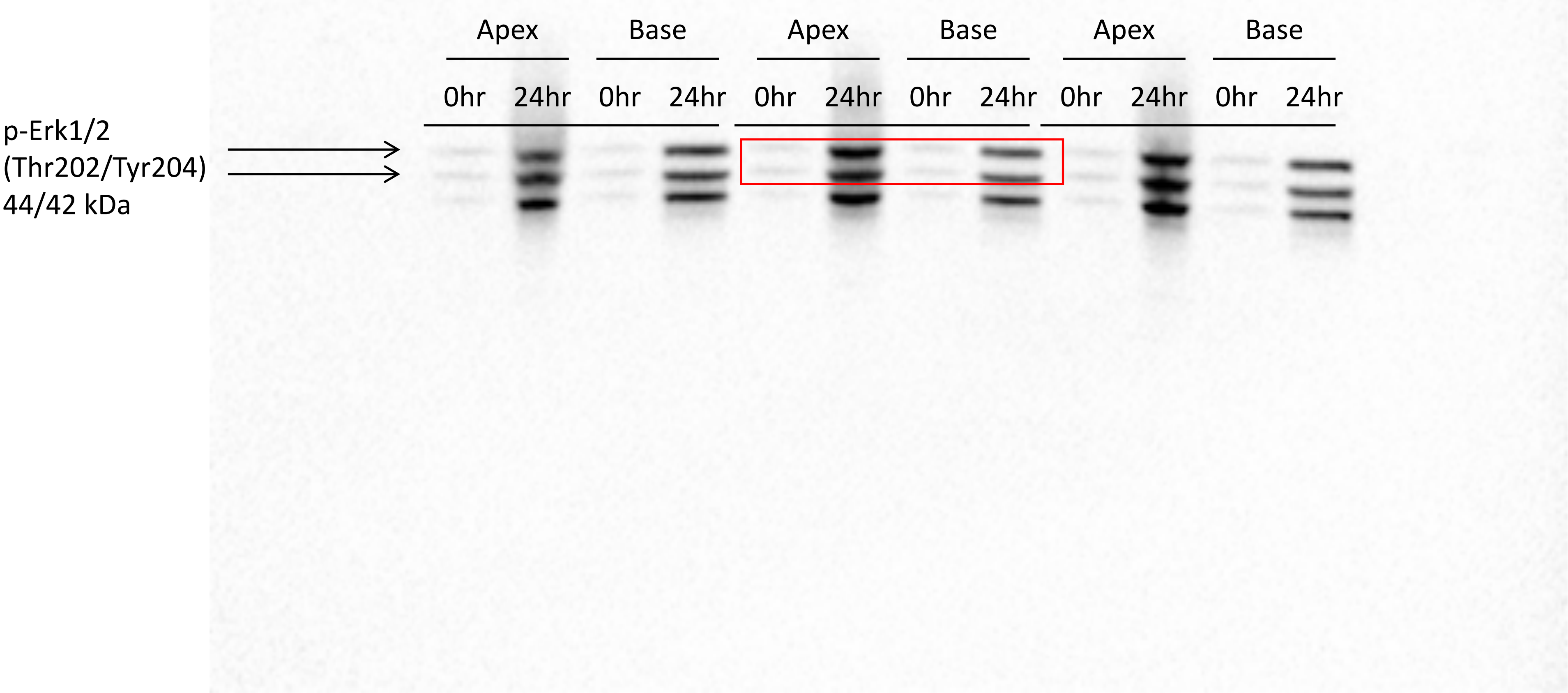

Full unedited gel for Figure 4B

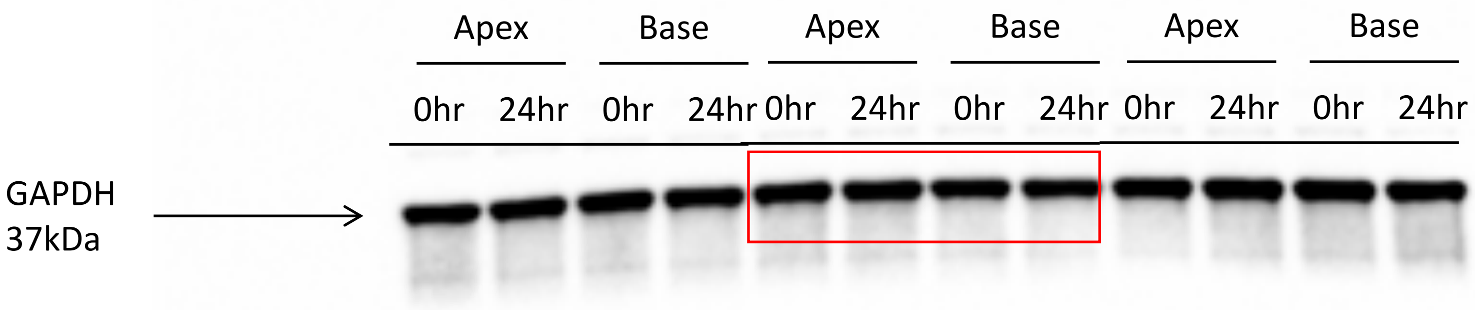

Full unedited gel for Figure 4C-CM (cytosolic fraction)

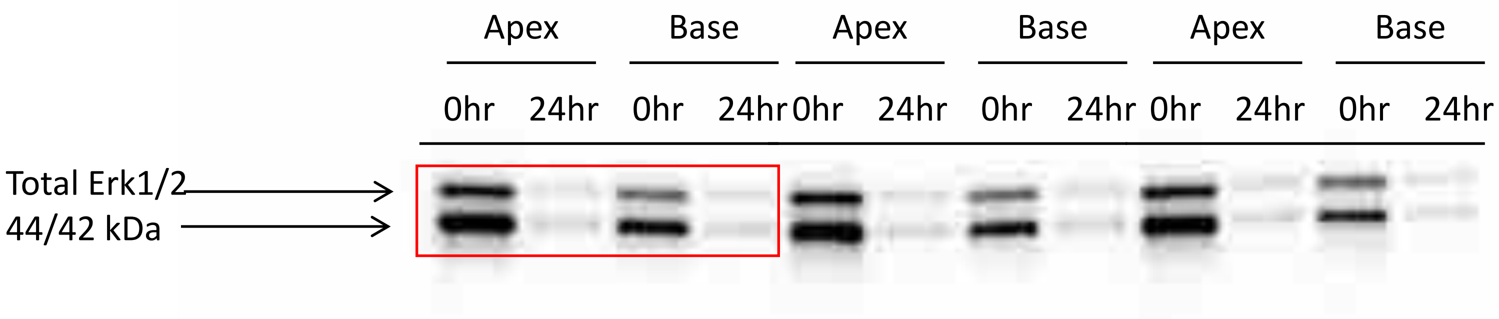

Full unedited gel for Figure 4C-(CM nuclear fraction)

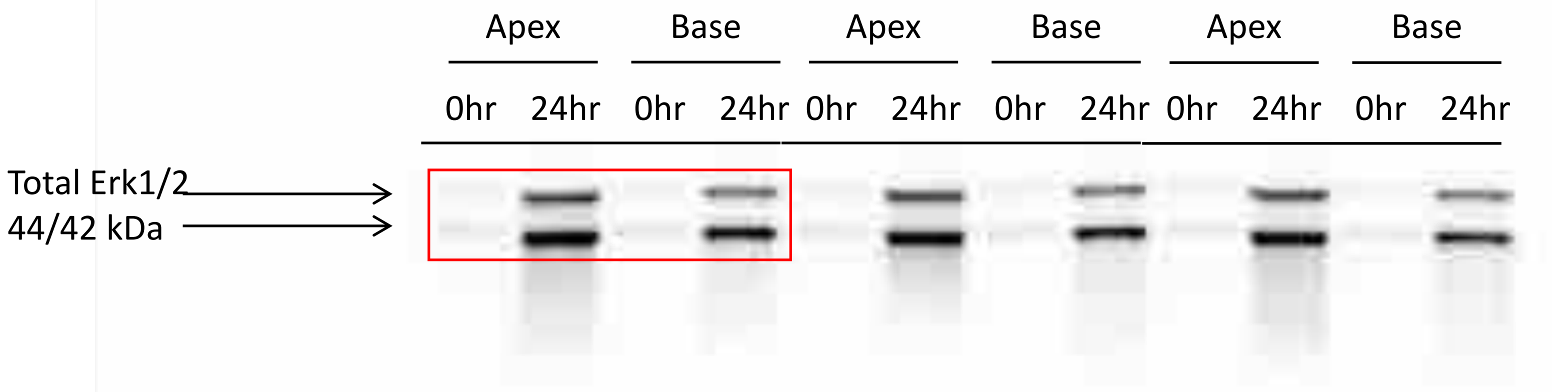

Full unedited gel for Figure 4C-(CM cytosolic fraction)

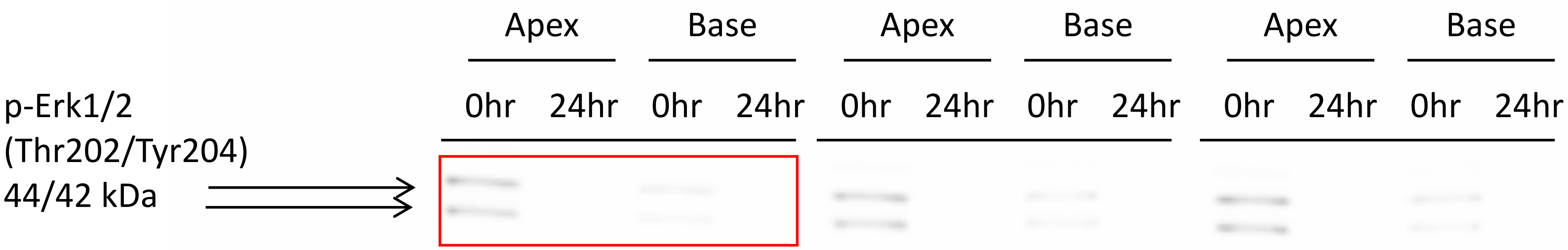

Full unedited gel for Figure 4C-(CM nuclear fraction)

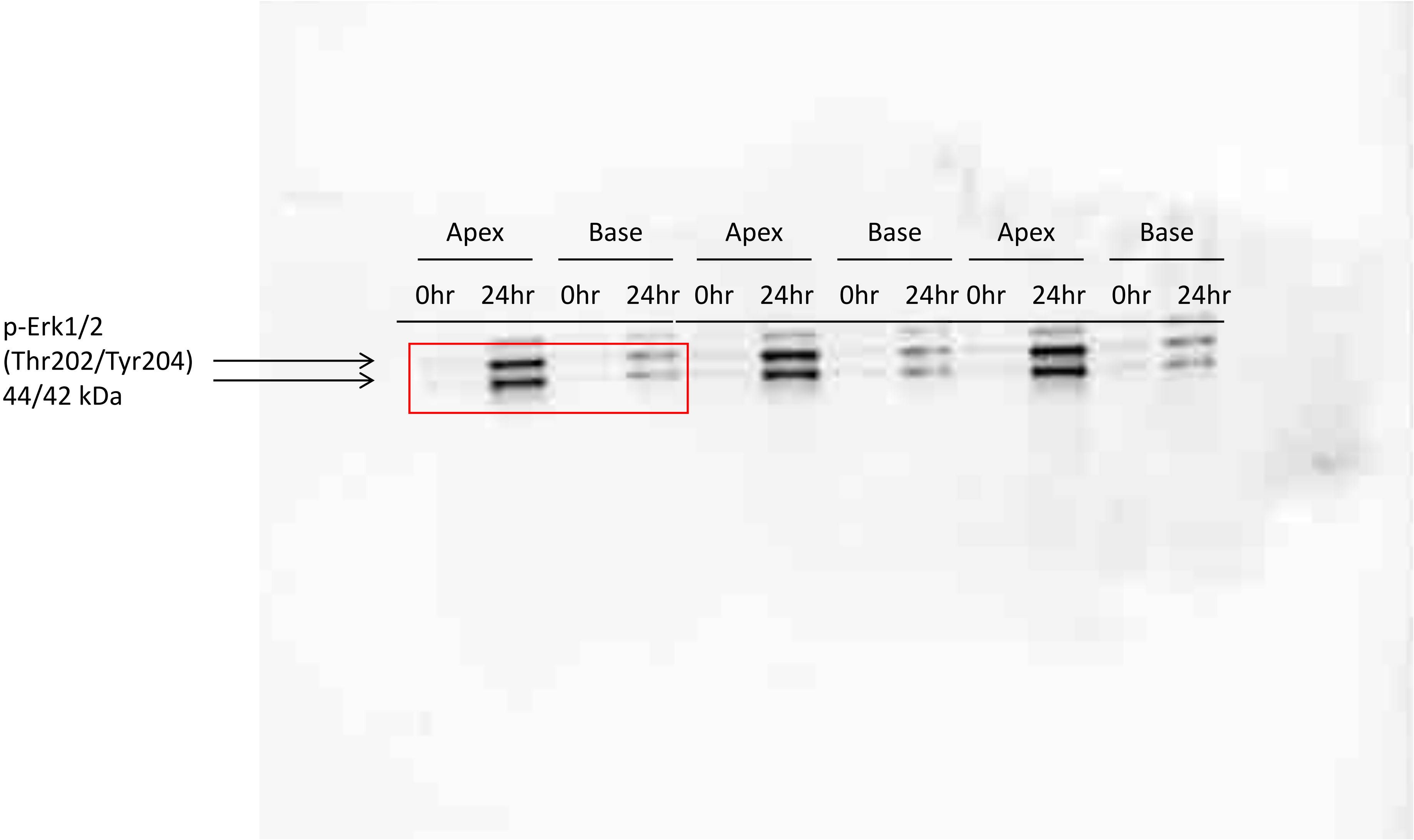

Full unedited gel for Figure 4C-(CM nuclear fraction)

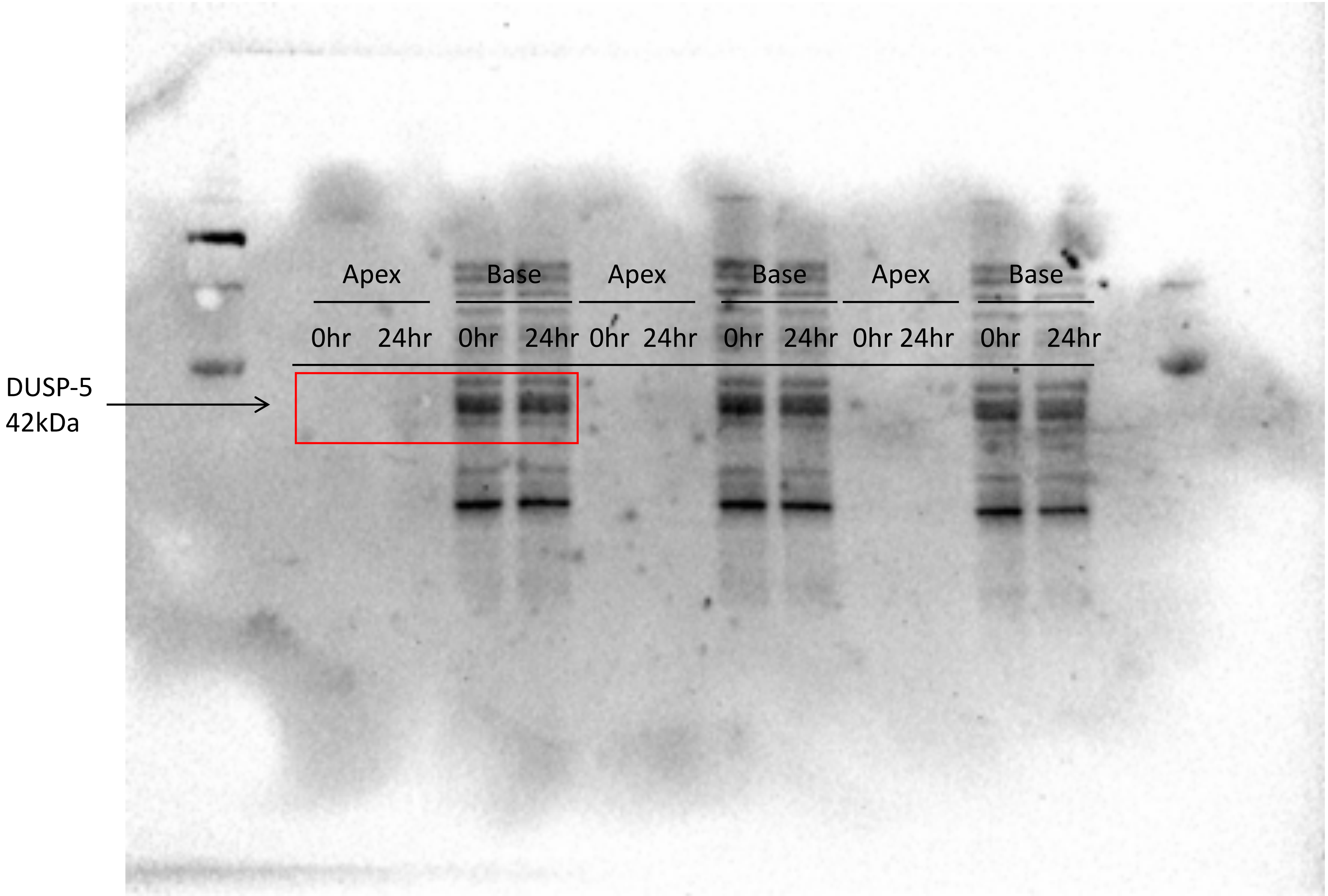

Full unedited gel for Figure 4C-(CM nuclear fraction)-over exposed

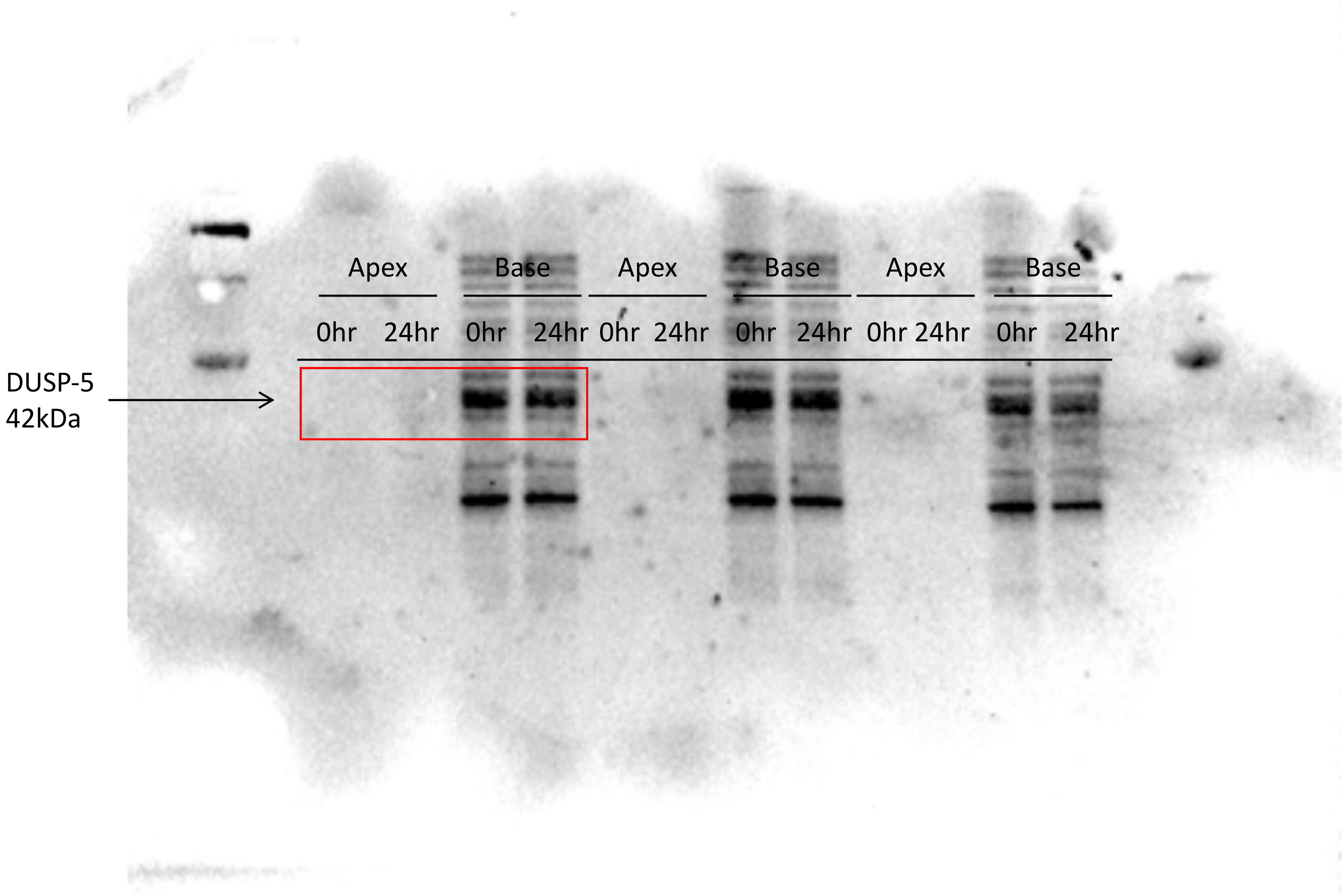

Full unedited gel for Figure 4C-(CM nuclear fraction)-under exposed

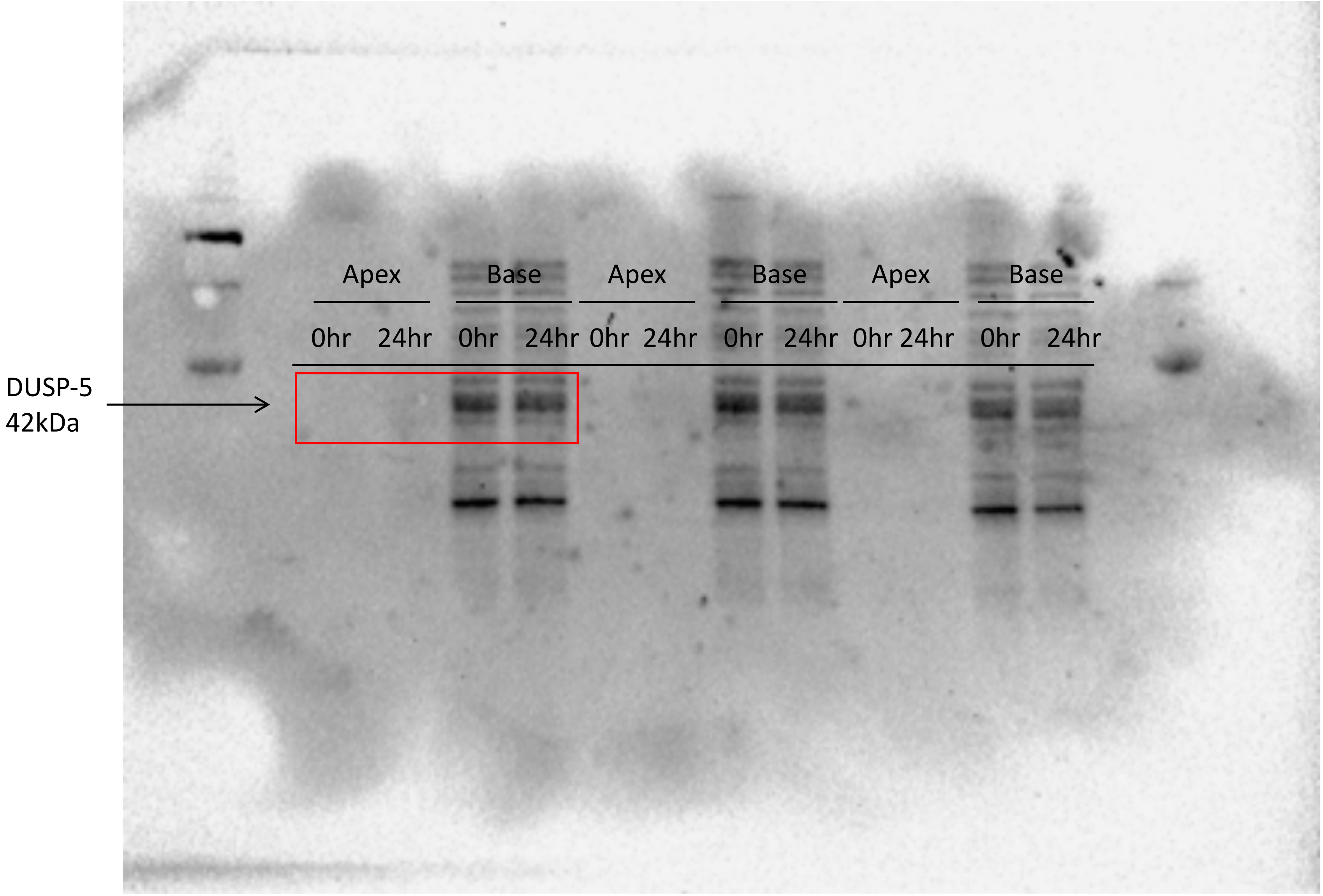

Full unedited gel for Figure 4C-(CM cytosolic fraction)

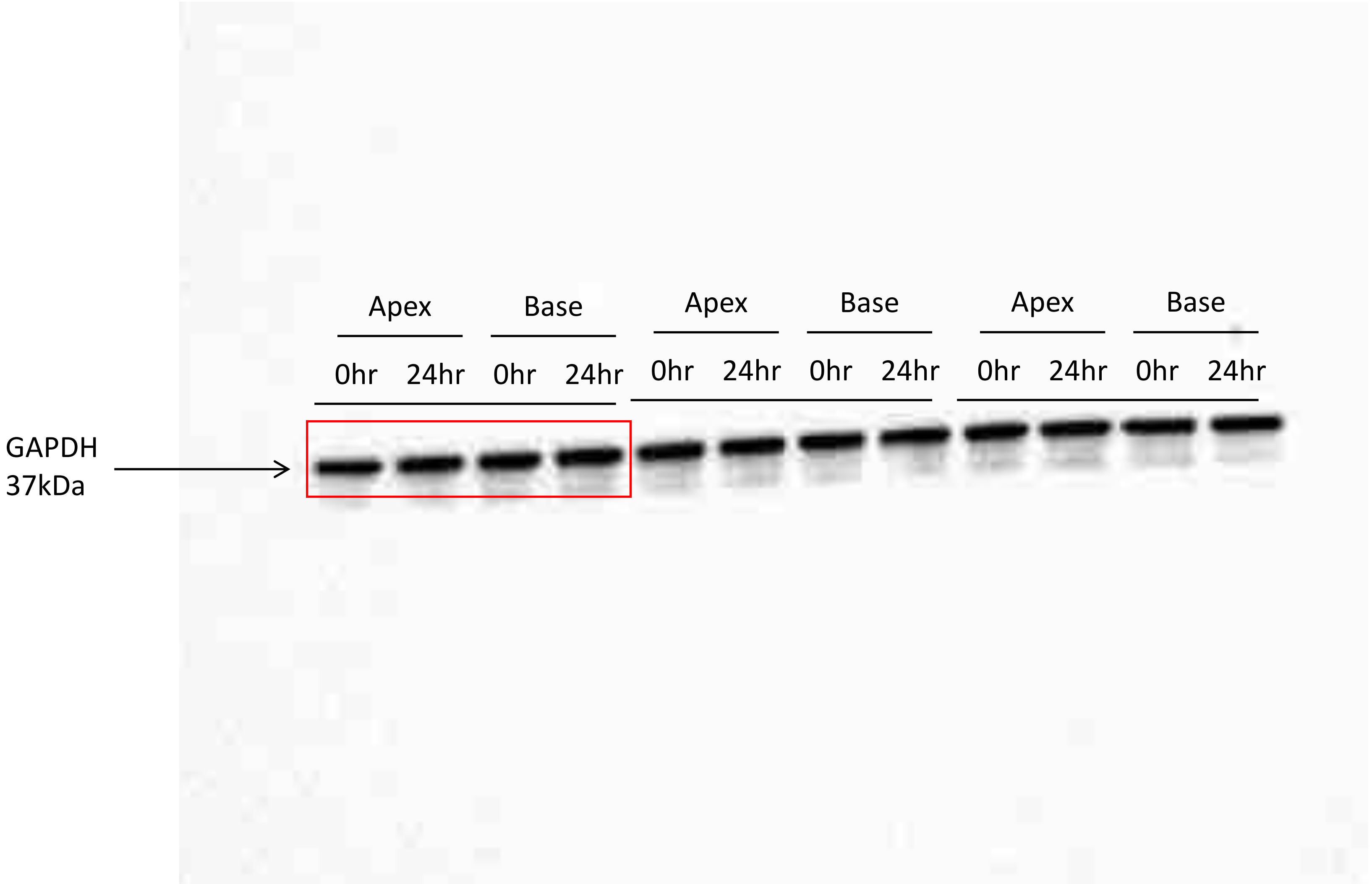

Full unedited gel for Figure 4C-(CM nuclear fraction)

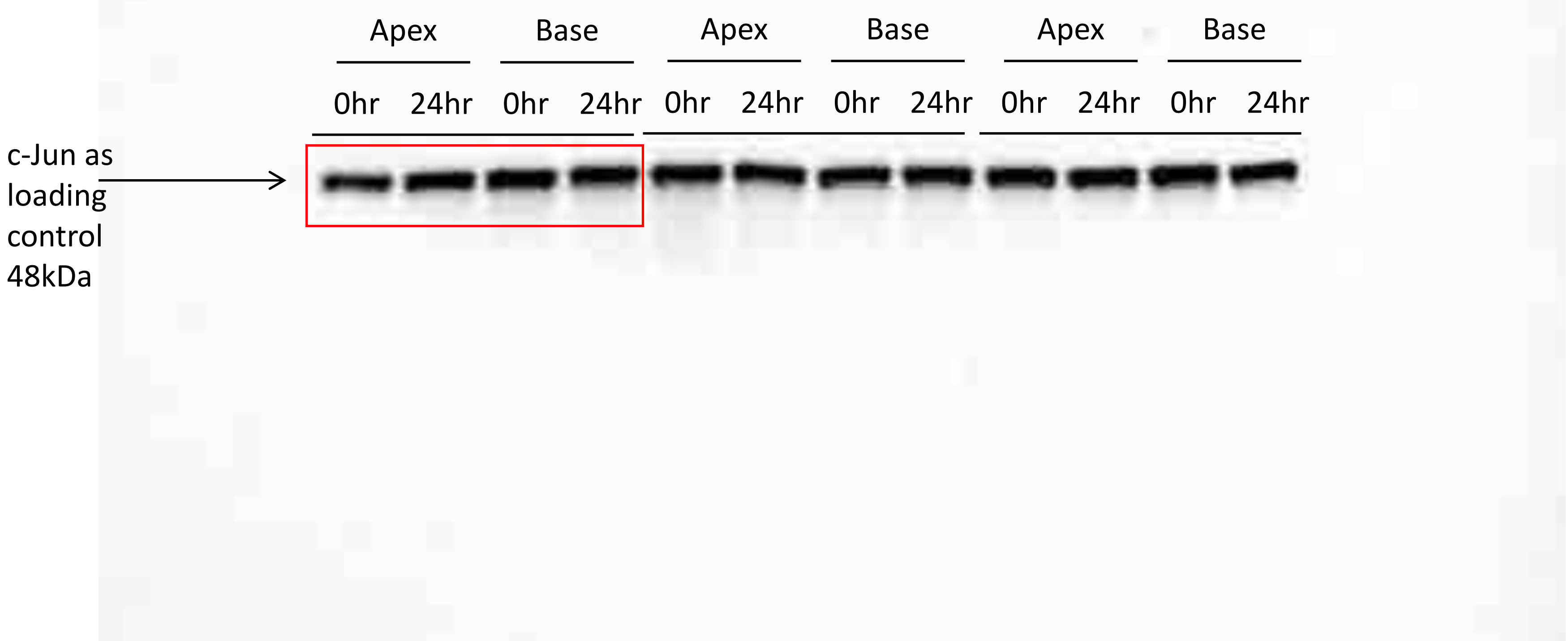

Full unedited gel for Figure 4D

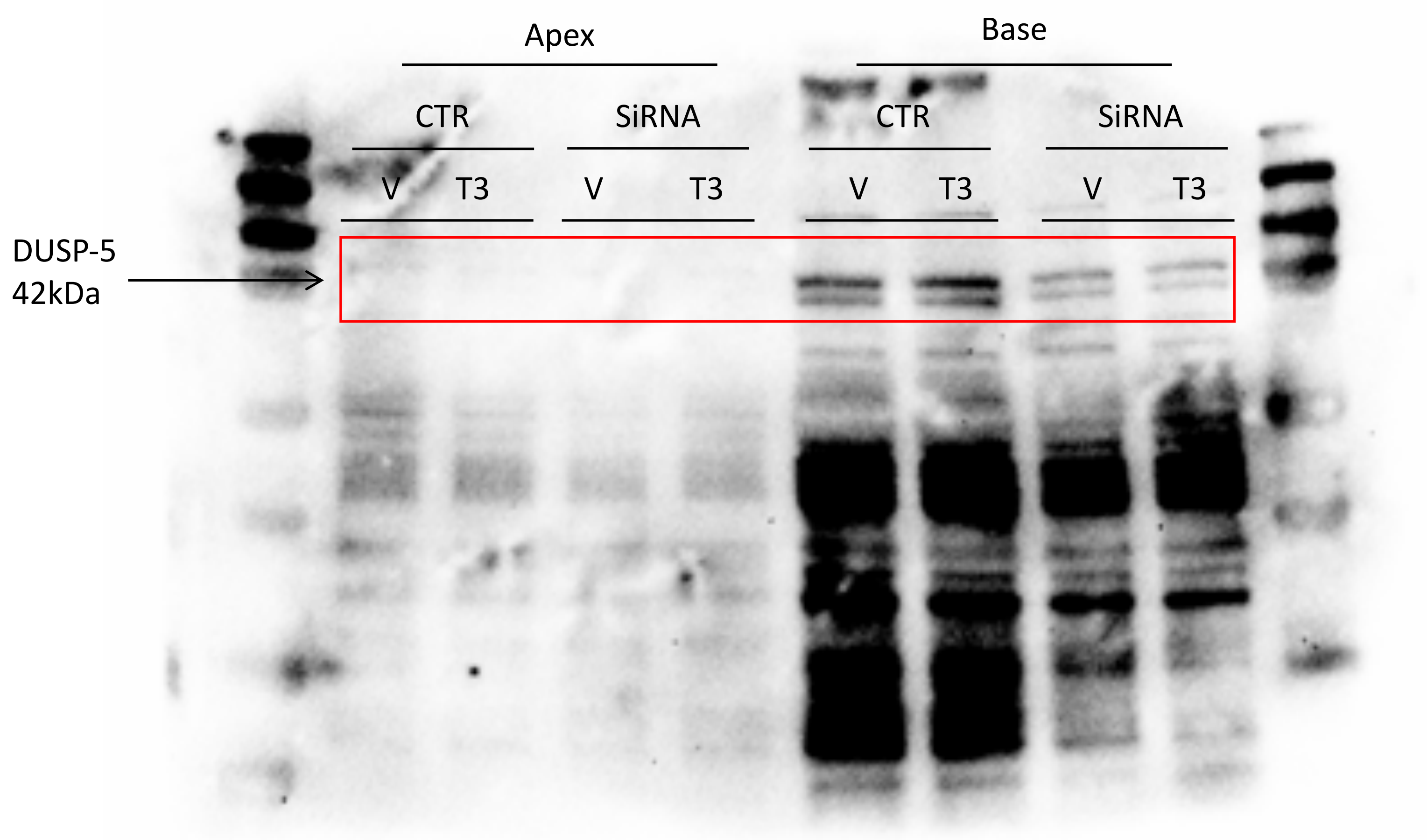

Full unedited gel for Figure 4D (over exposed)

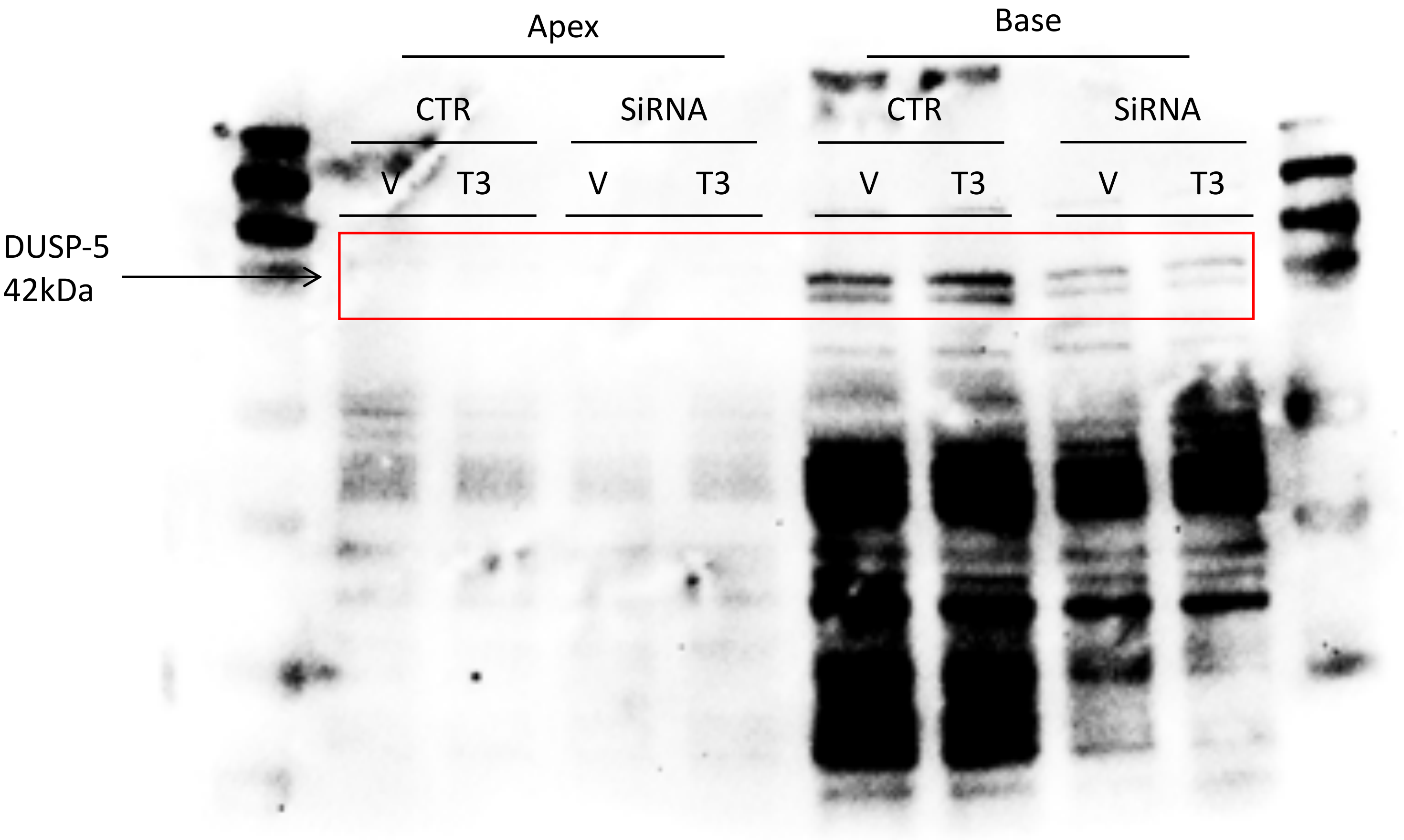

Full unedited gel for Figure 4D (under exposed)

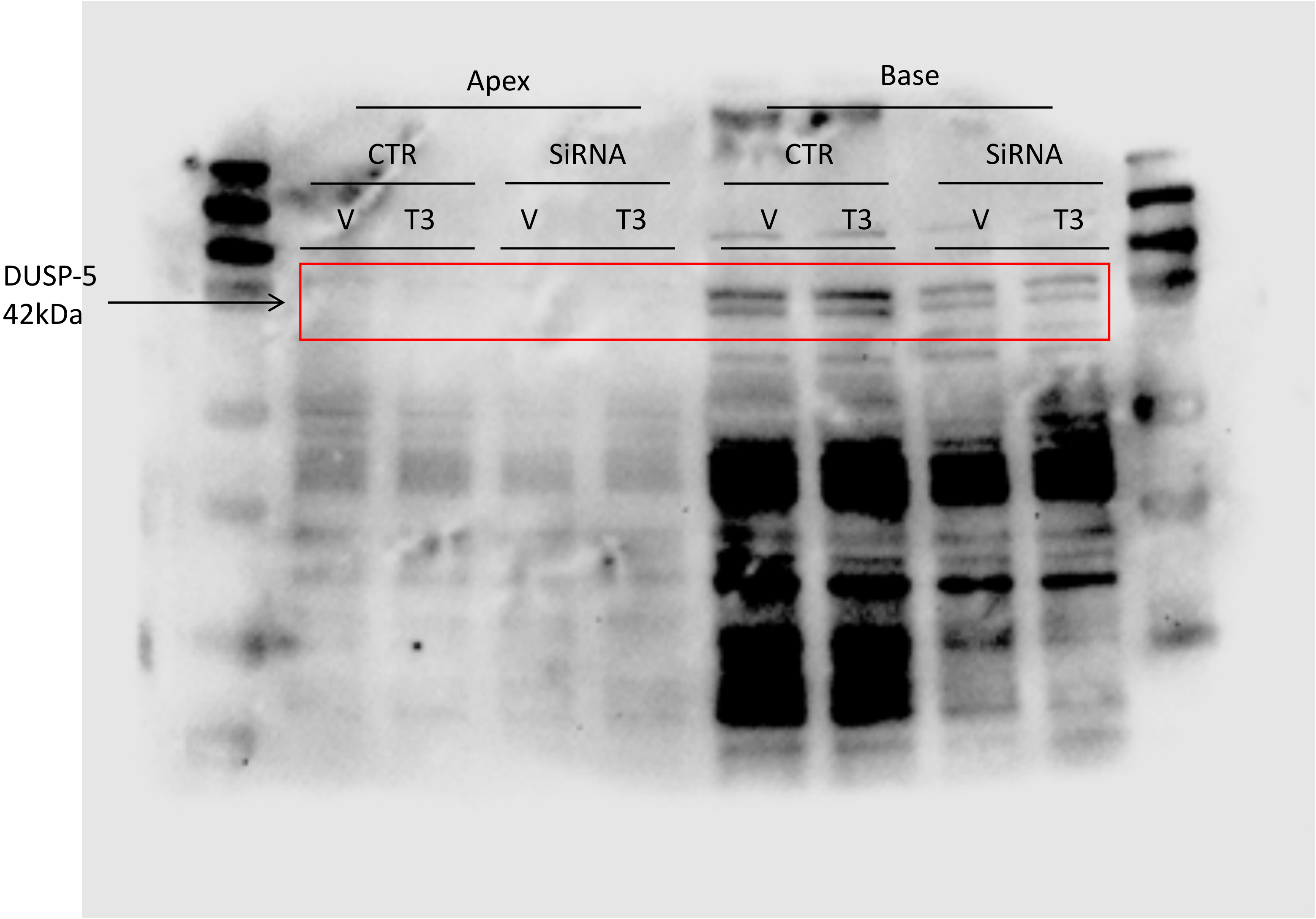

Full unedited gel for Figure 4D

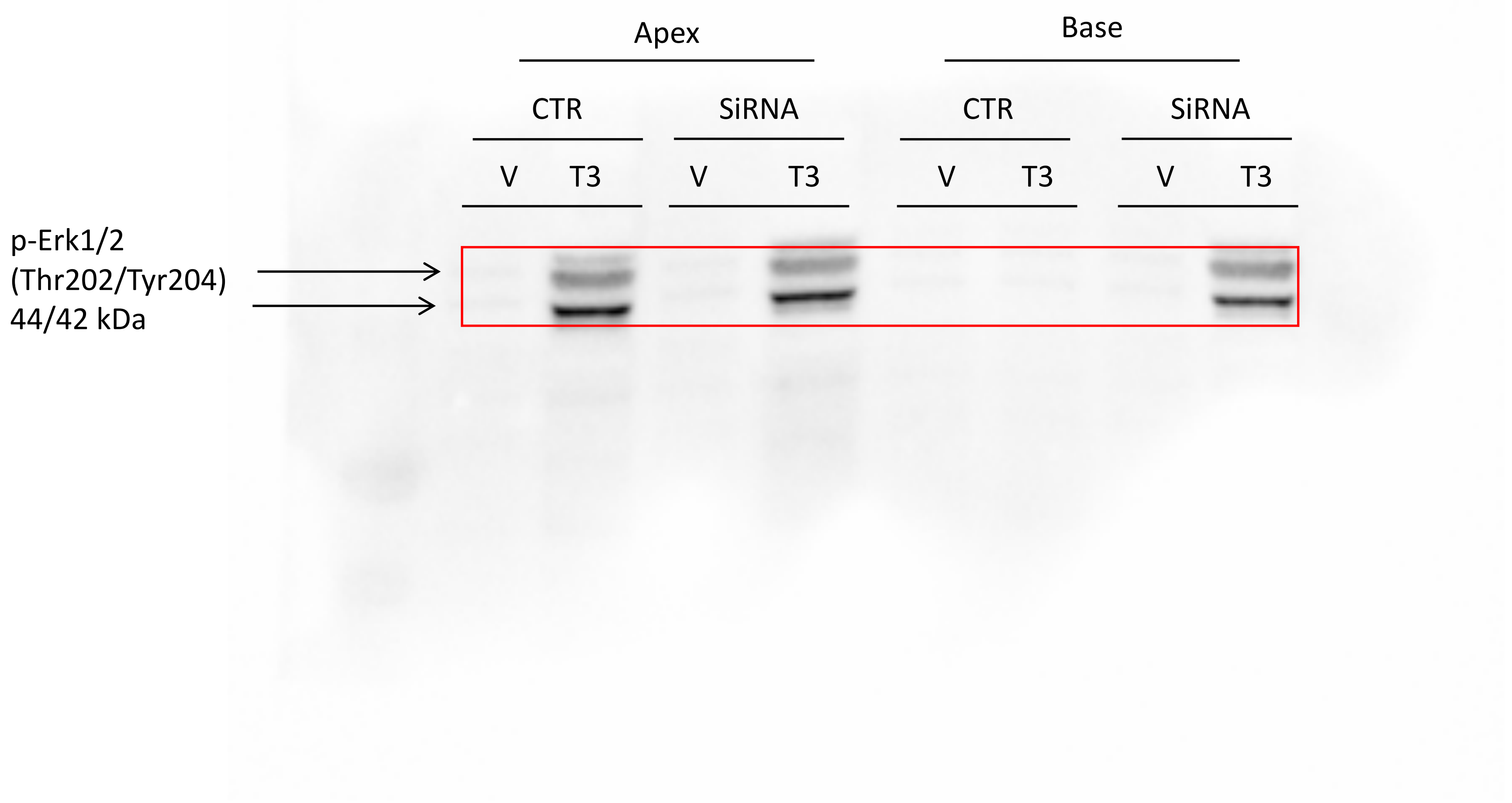

Full unedited gel for Figure 4D

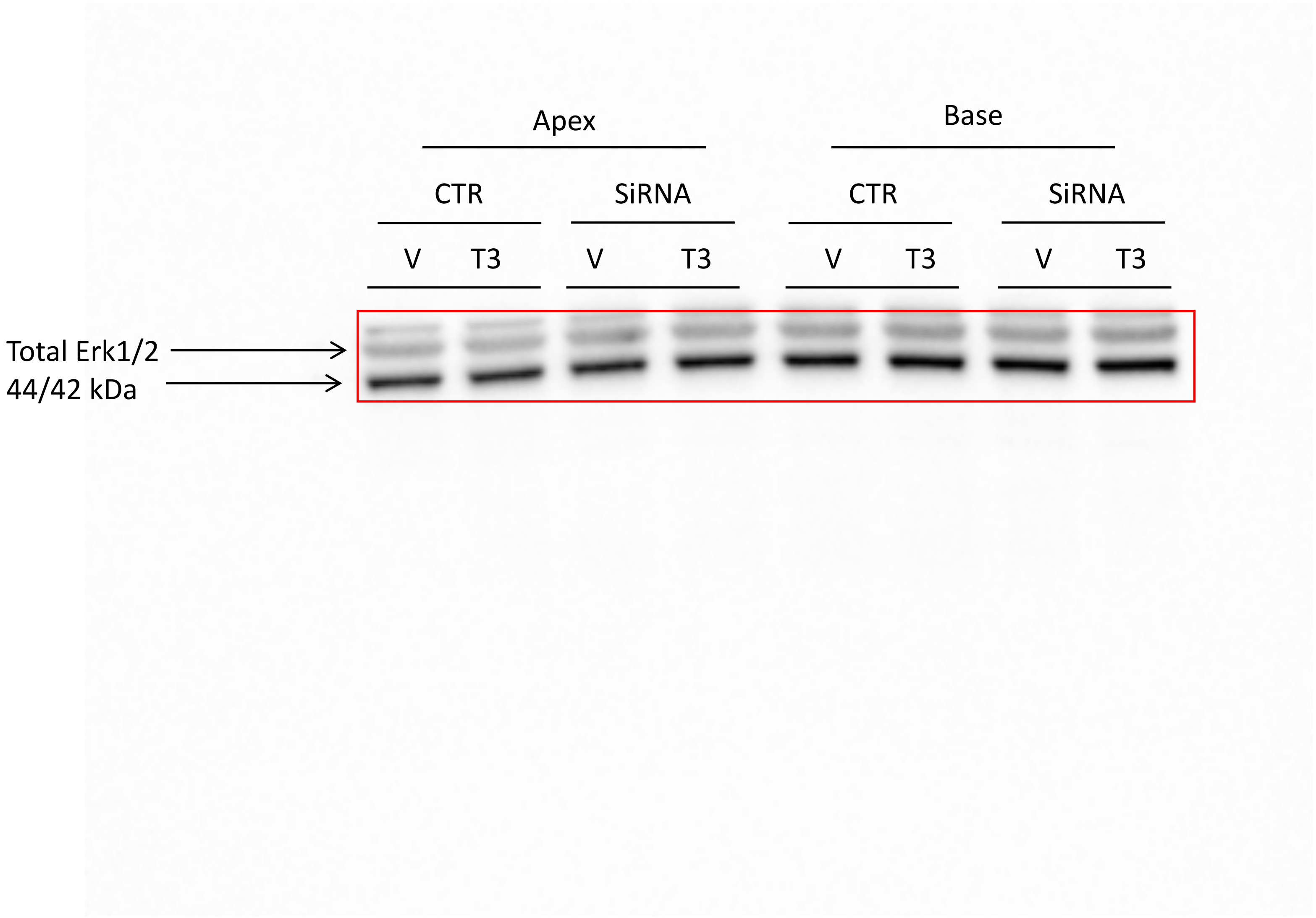

Full unedited gel for Figure 4D

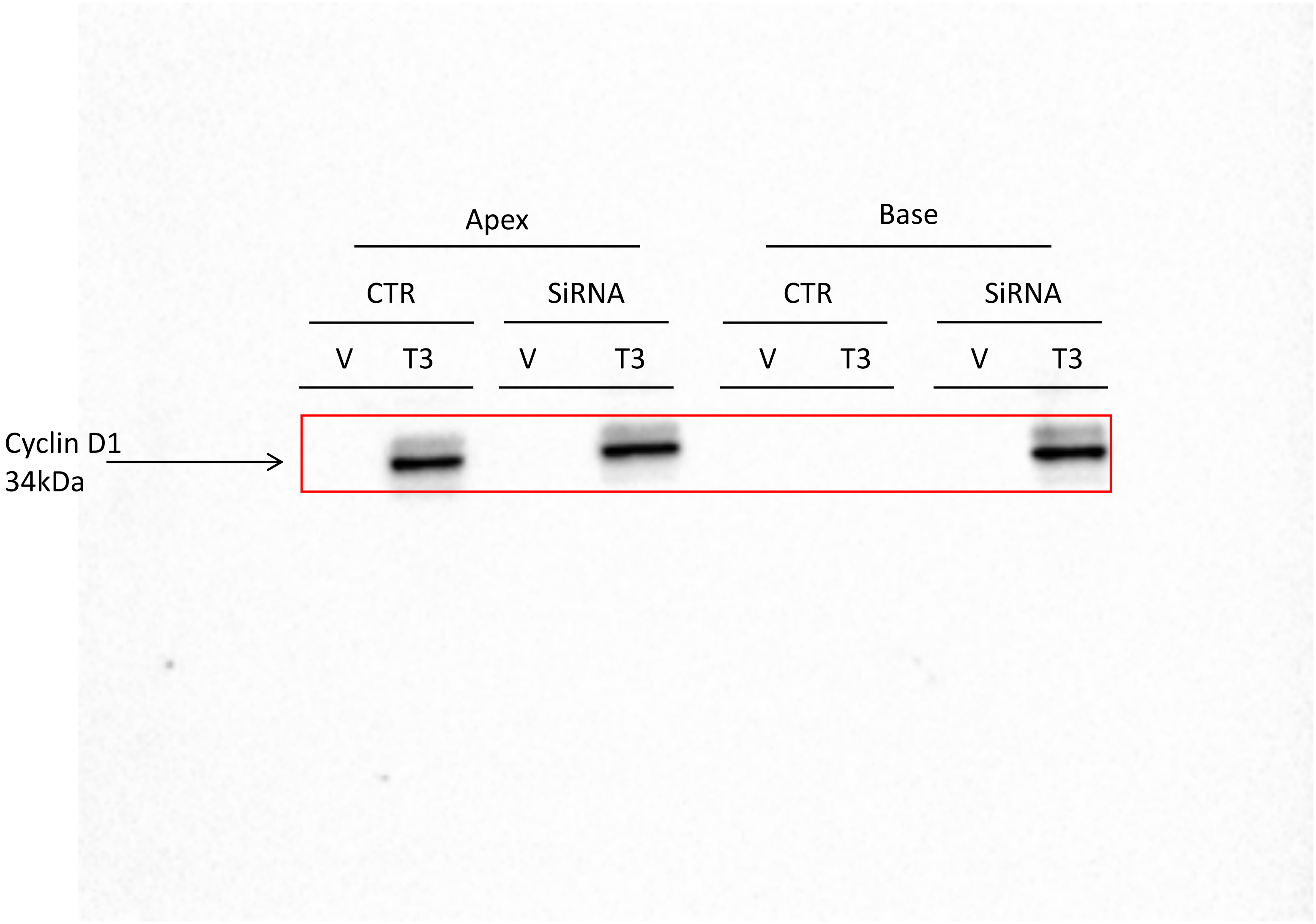

Full unedited gel for Figure 4D

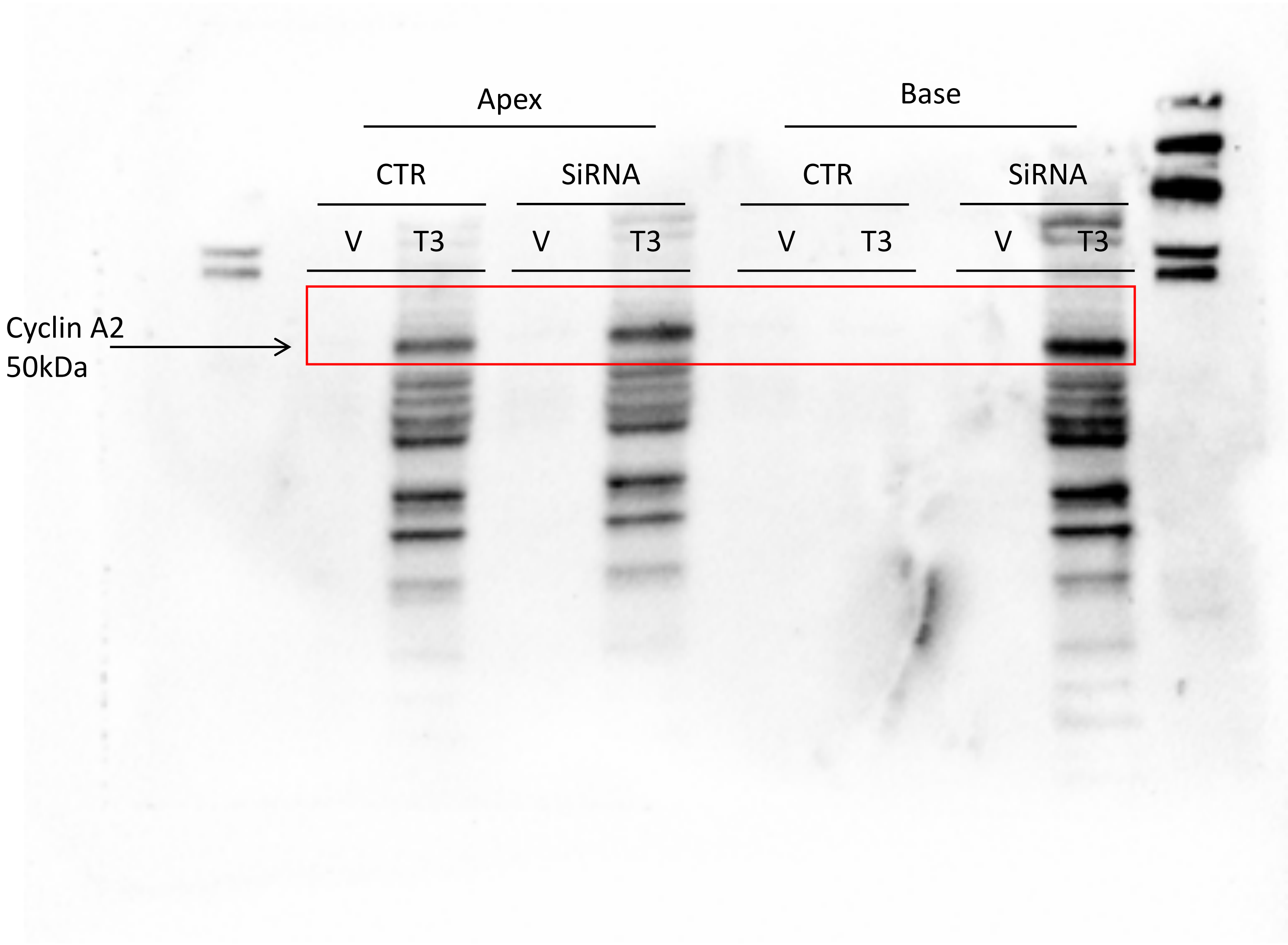

Full unedited gel for Figure 4D

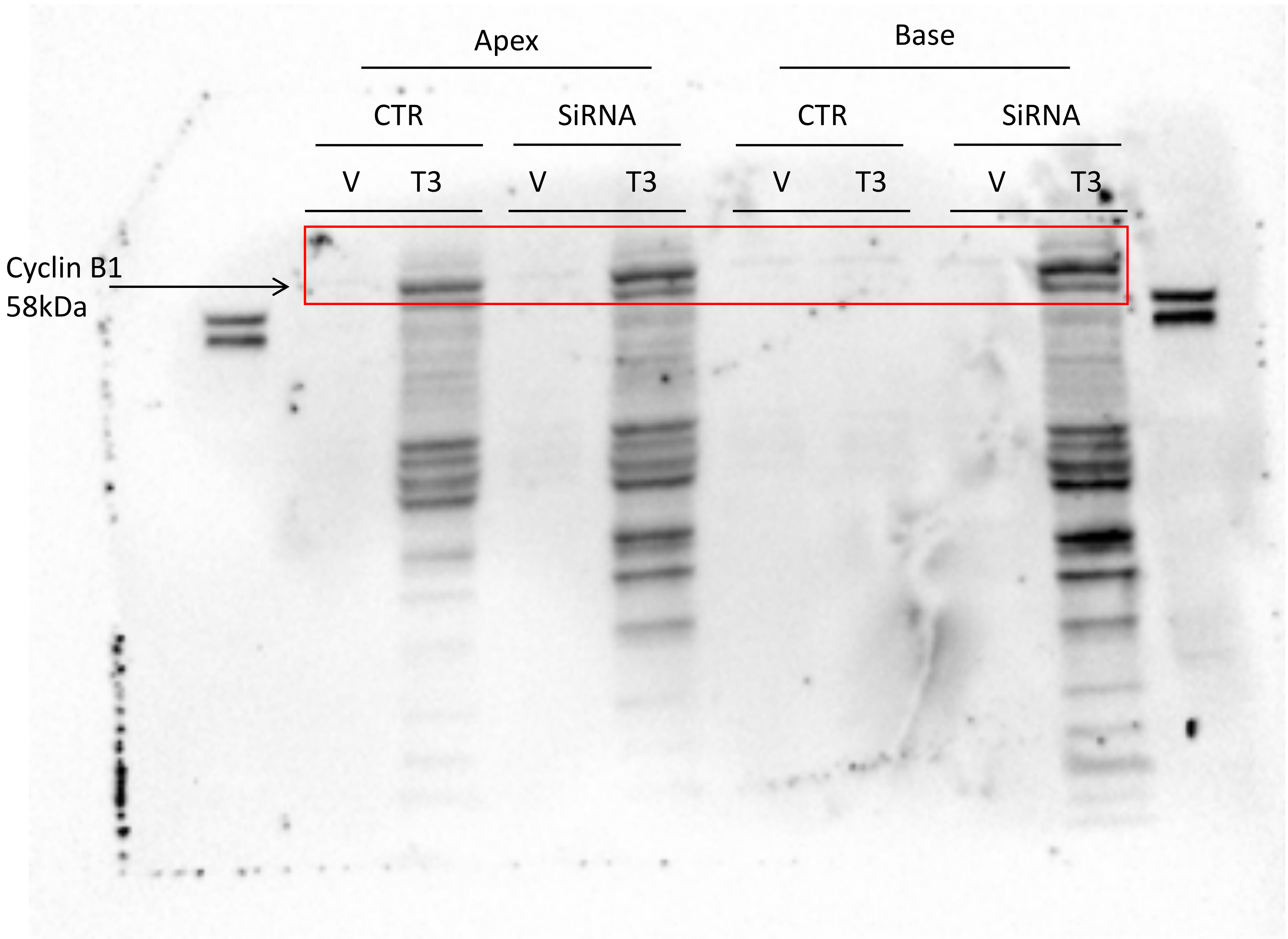

Full unedited gel for Figure 4D

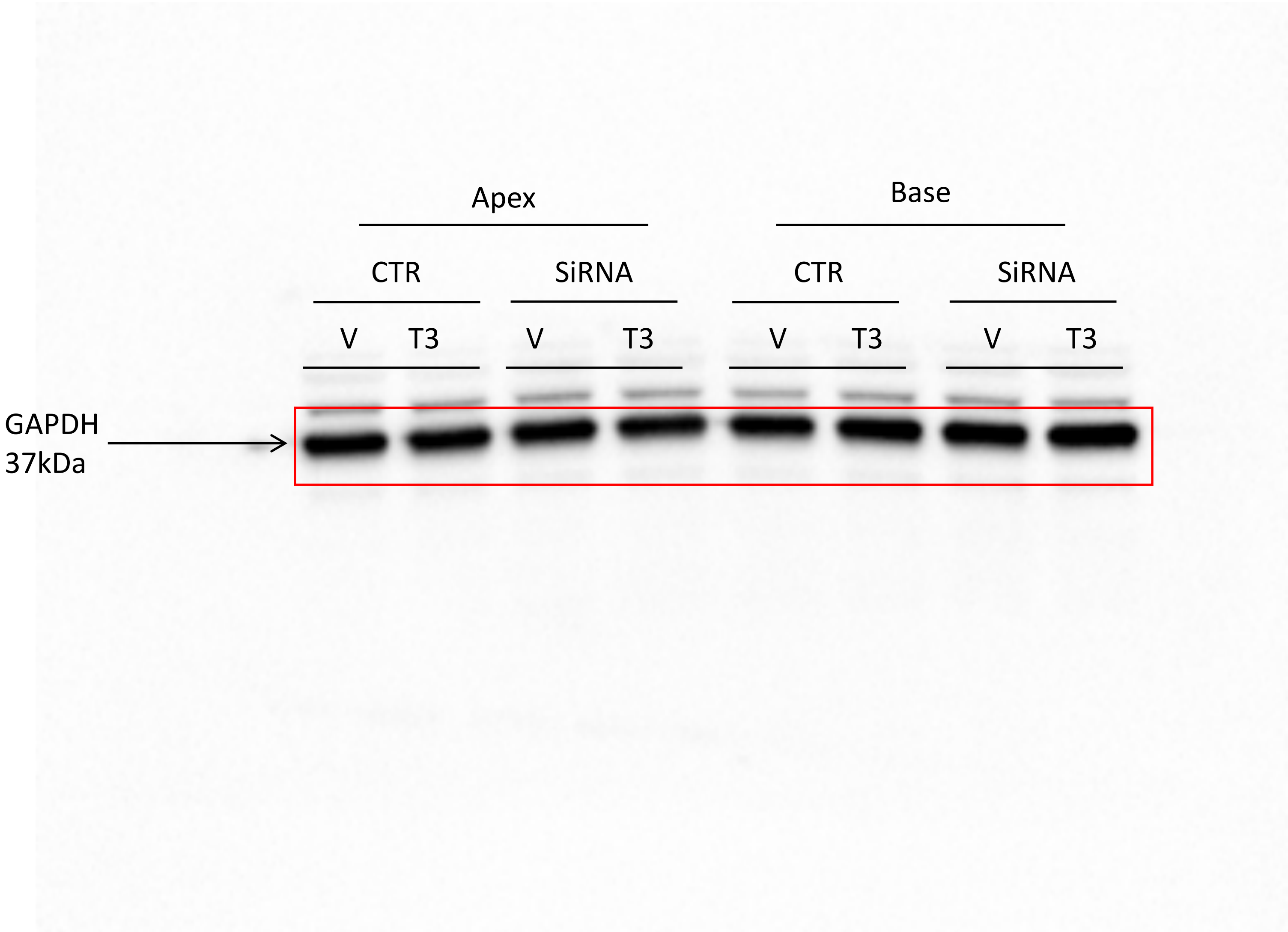

Full unedited gel for Figure 6A

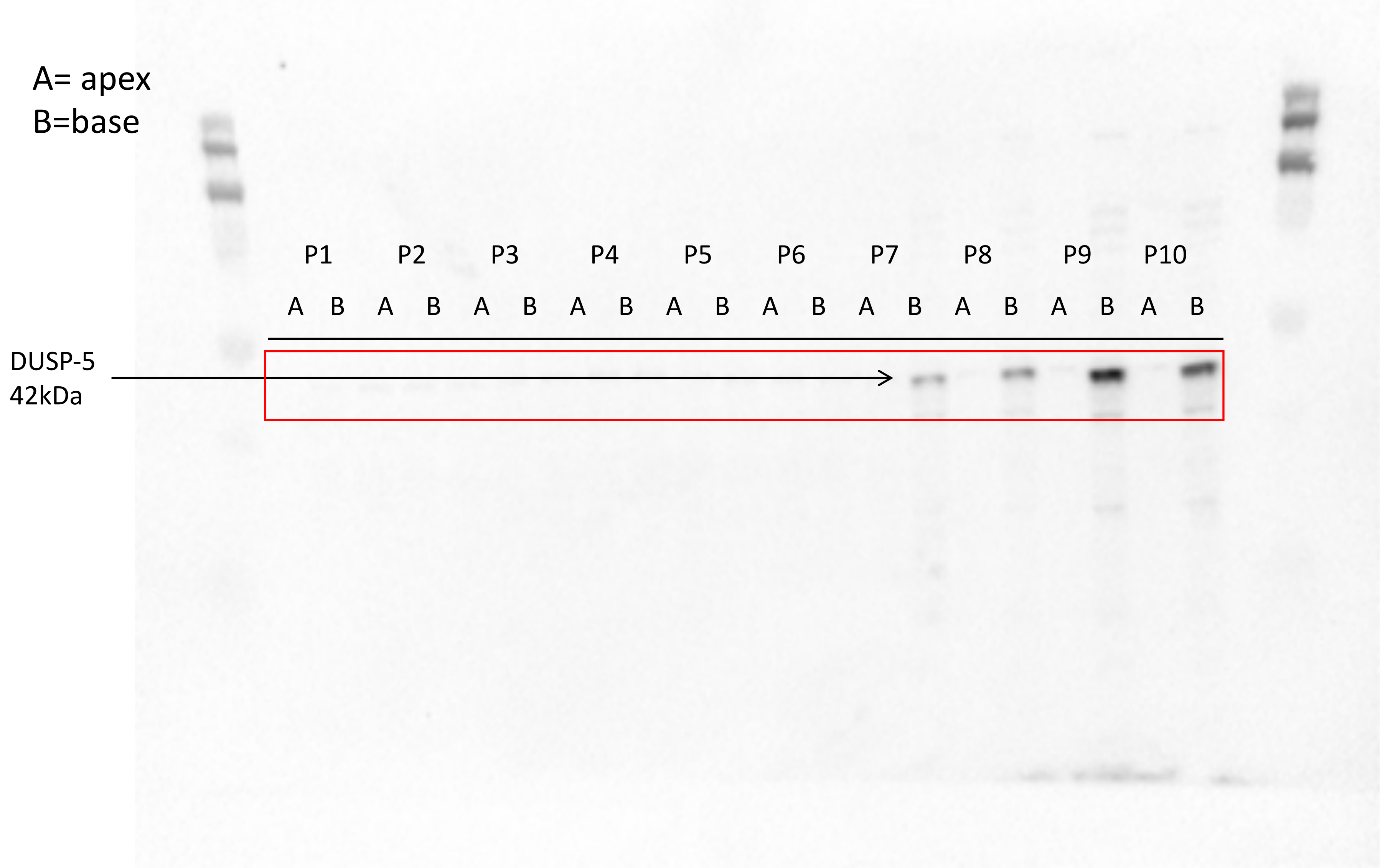

Full unedited gel for Figure 6A

A= apex
B=base

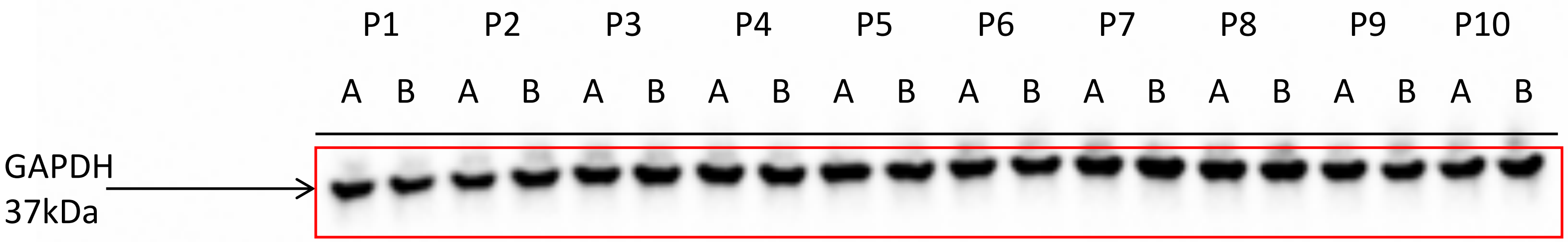

Full unedited gel for Figure 6A

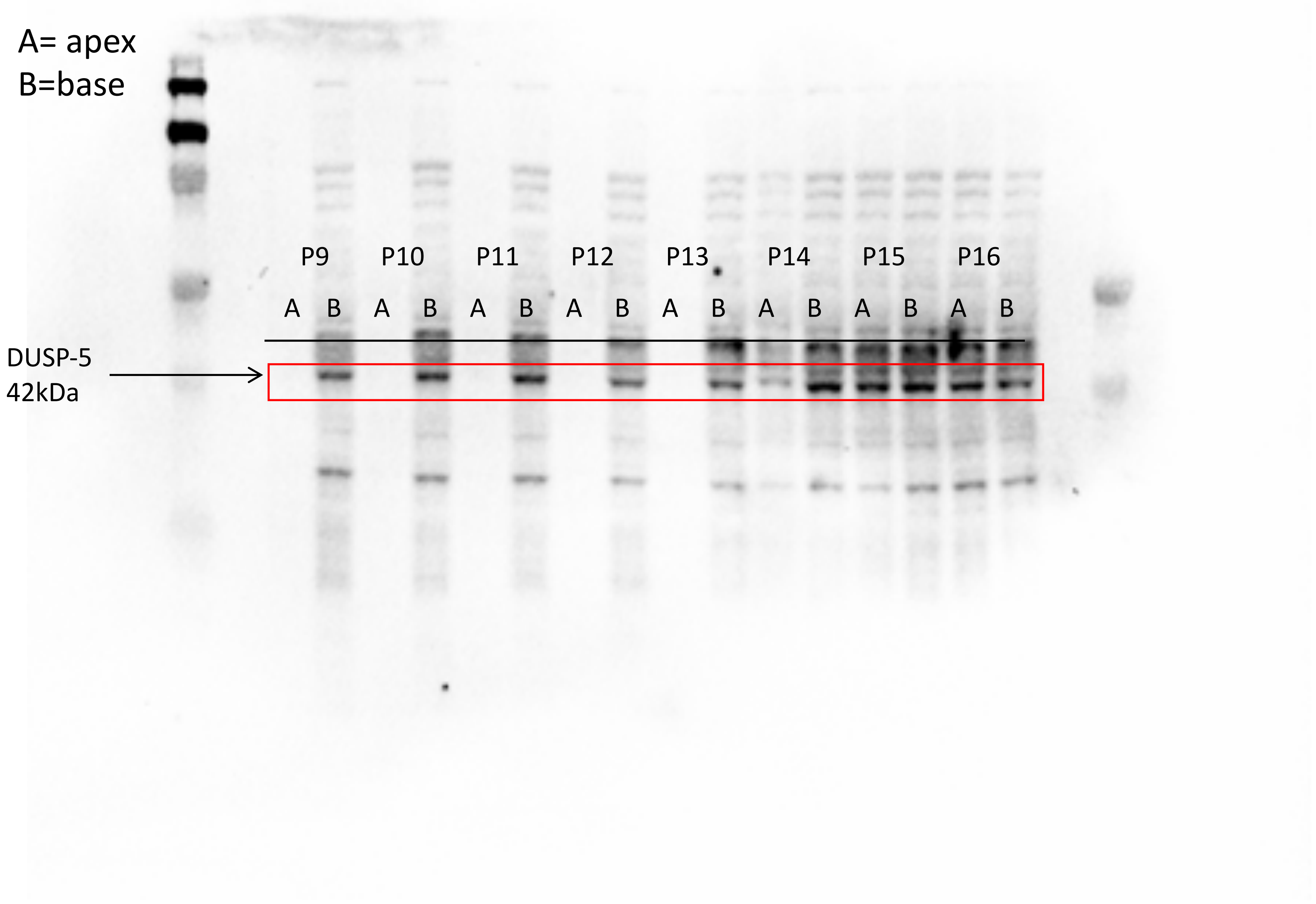

Full unedited gel for Figure 6A

A= apex
B=base

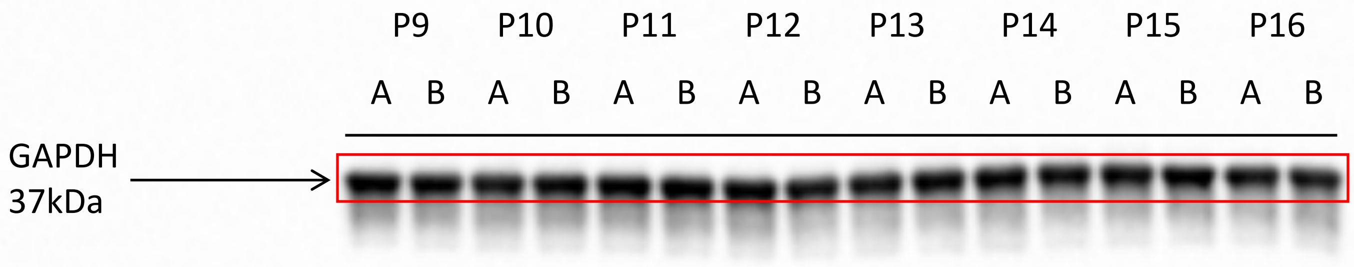

Full unedited gel for Figure 6B

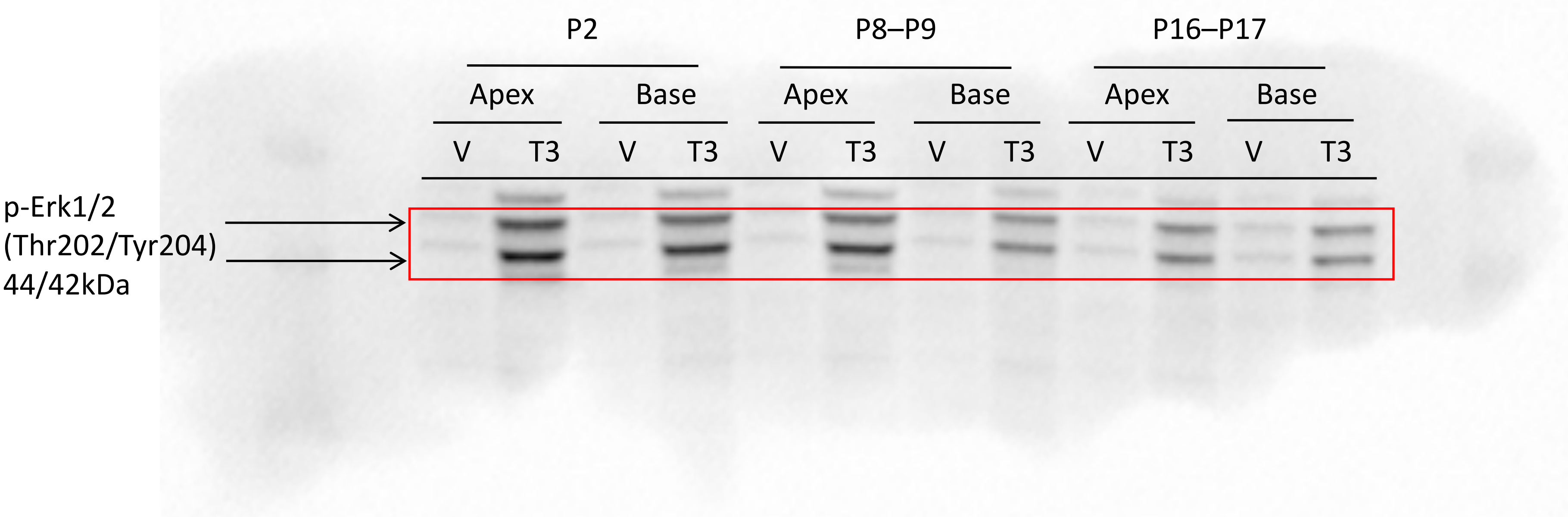

Full unedited gel for Figure 6B

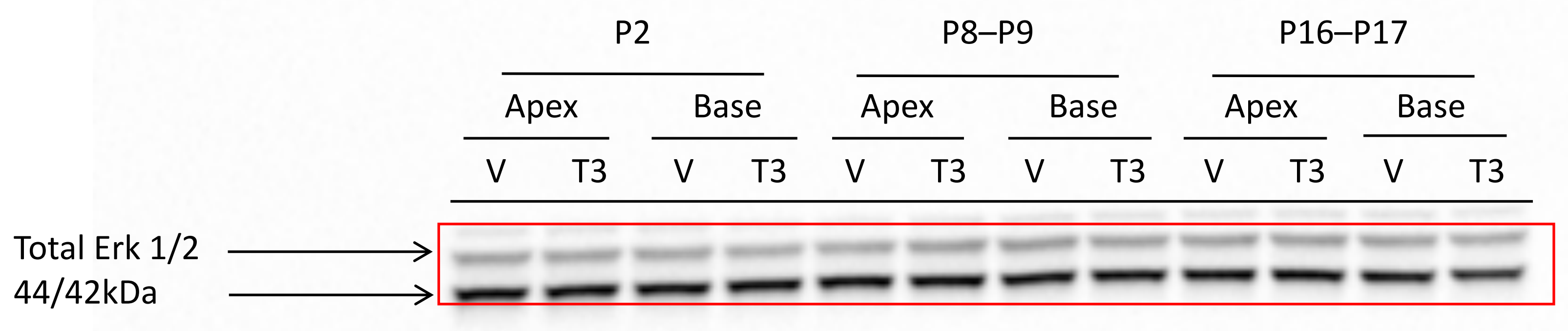

Full unedited gel for Figure 6B

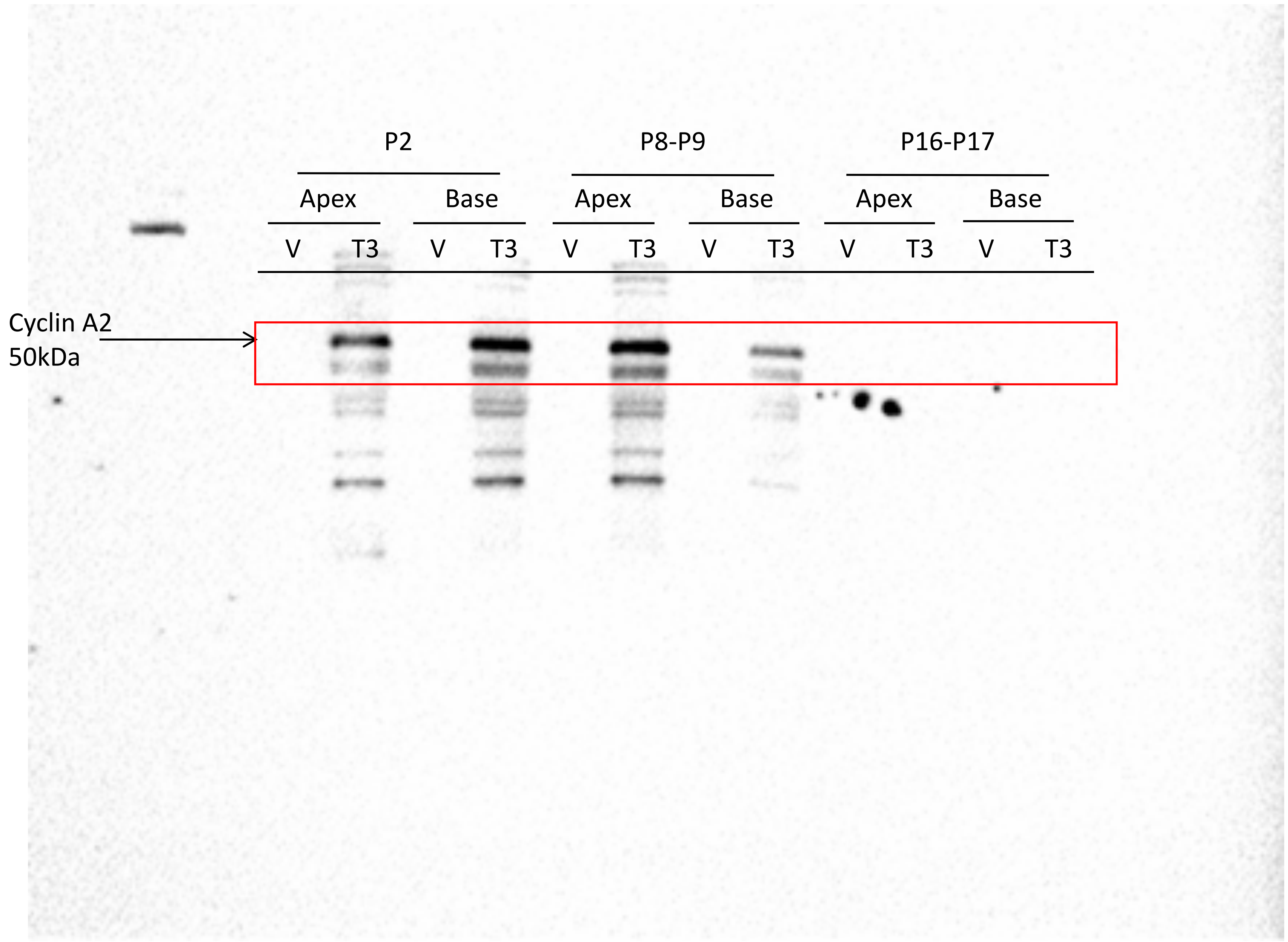

Full unedited gel for Figure 6B

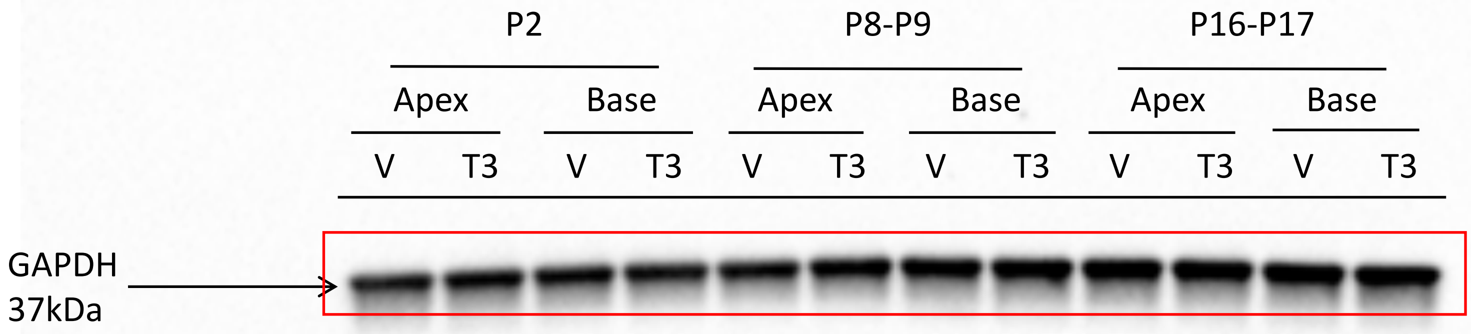

Full unedited gel for figure S1

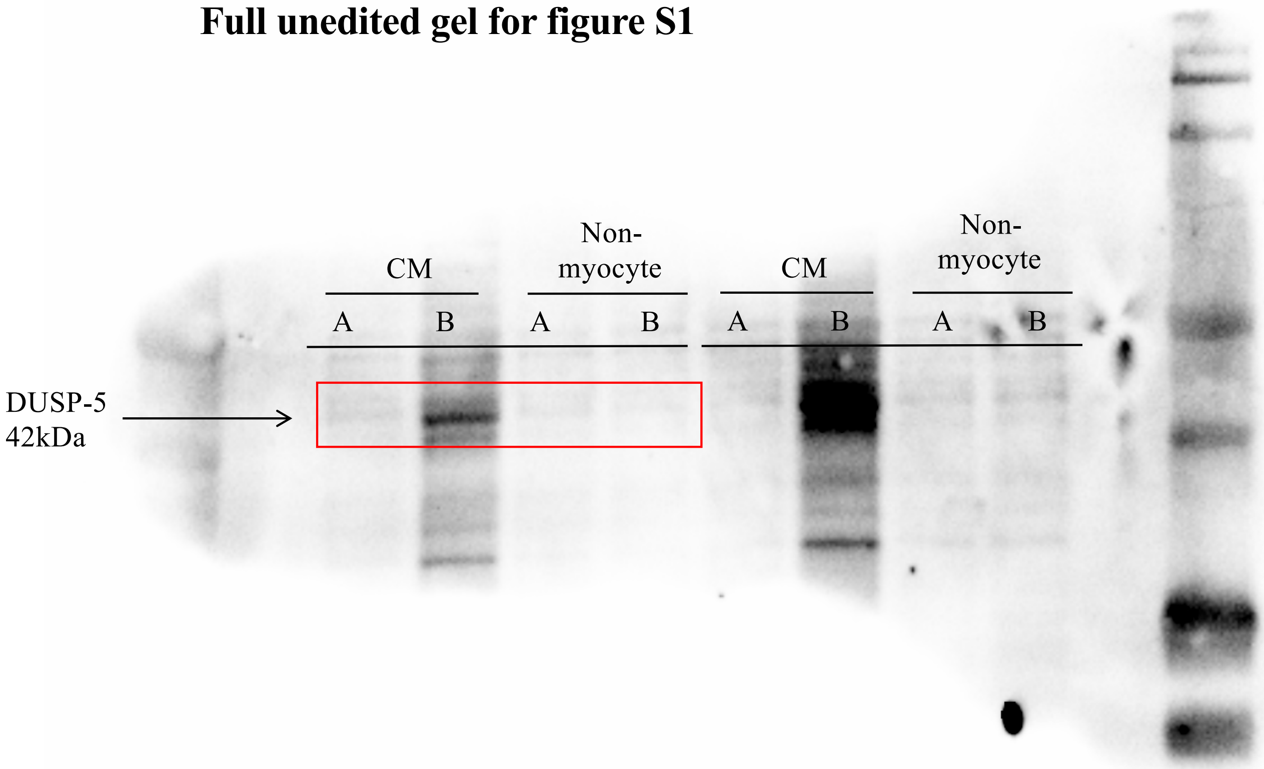

Full unedited gel for figure S1

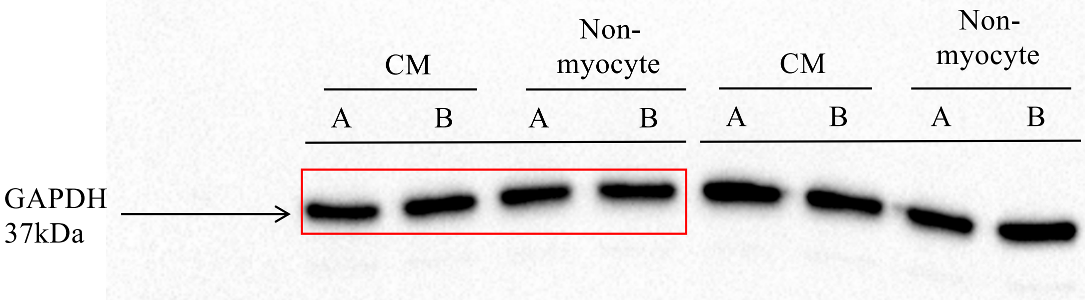

Full unedited gel for figure S2

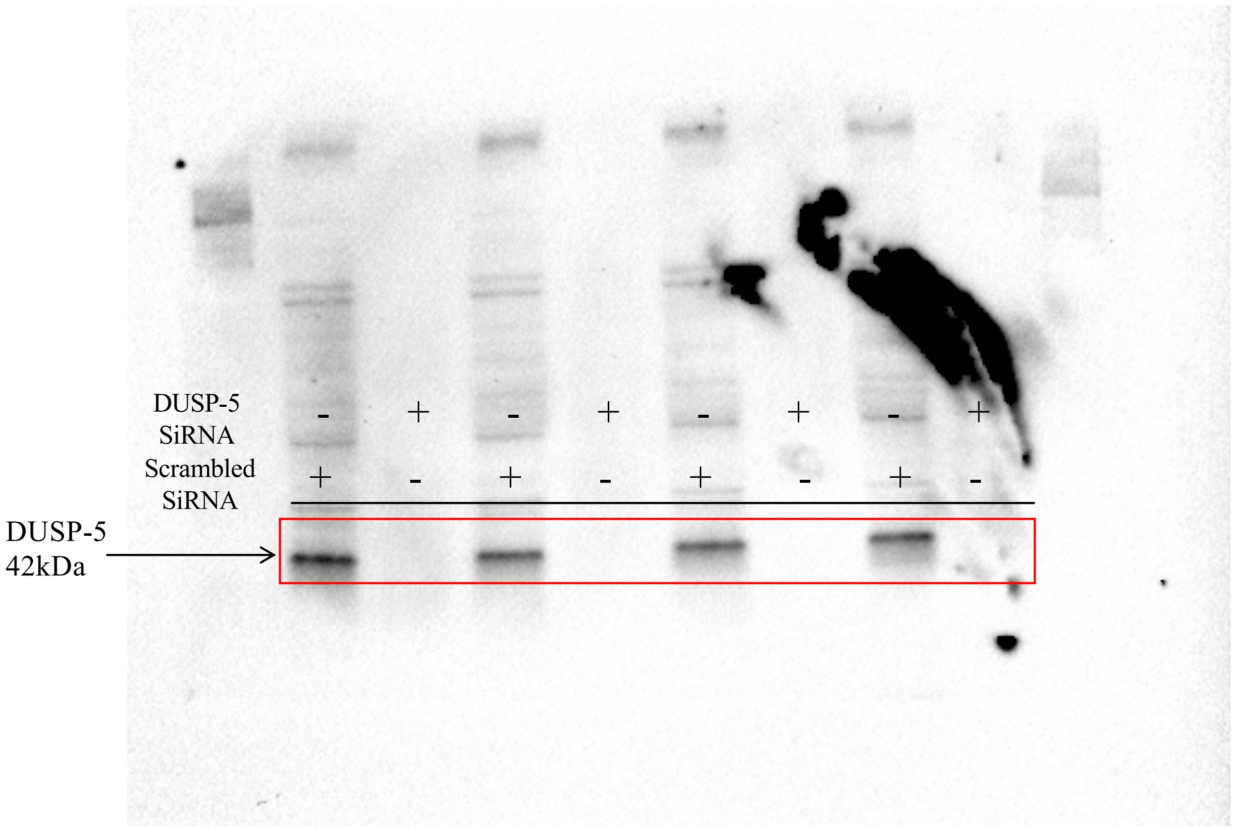

Full unedited gel for figure S2

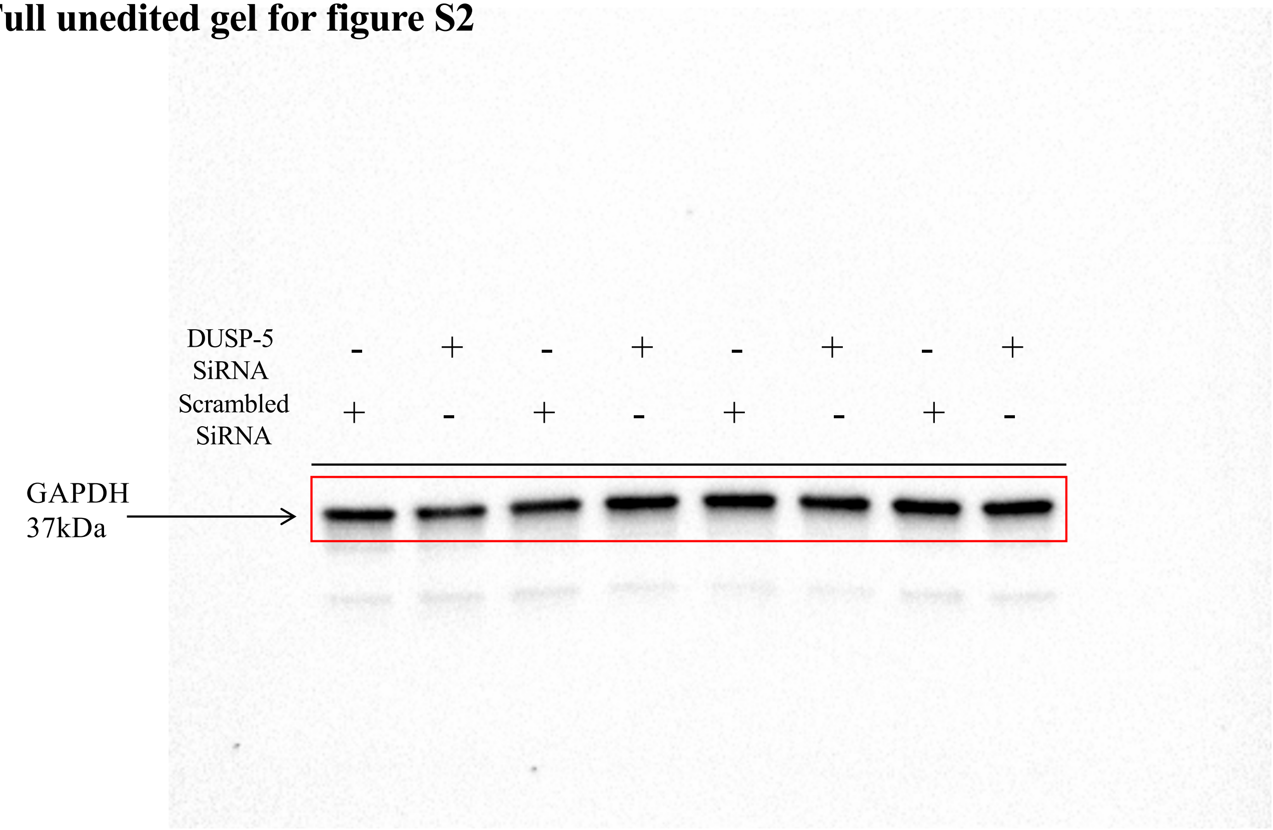

Supplement: Supplementary file 1 — Supplementary Information [file 41598_2020_78825_MOESM1_ESM.pdf]
